# Mediterranean-ketogenic vs. medium-chain triglyceride supplemented Mediterranean diet: Effects on the gut-brain-immune axis in Parkinson’s disease

**DOI:** 10.1016/j.isci.2026.117981

**Published:** 2026-10-28

**Authors:** Kira Tosefsky, Samantha L. Schaffner, Joyce S.T. Lam, Yolanda N. Wang, Shayan Keymanesh, Annie J. Kuan, Avril Metcalfe-Roach, Mihai S. Cirstea, Matthew A. Sacheli, Tessa F. Morelli, Matthew Yap, Olivia Brigden, Madison Gill, Tamara R. Cohen, Sophie Stukas, B. Brett Finlay, Silke Appel-Cresswell

**Affiliations:** 1 Pacific Parkinson’s Research Centre, Djavad Mowafaghian Centre for Brain Health, University of British Columbia, Vancouver, BC V6T 1Z3, Canada; 2 MD/PhD Program, Faculty of Medicine, University of British Columbia, Vancouver, BC, Canada; 3 Faculty of Land and Food Systems, Food, Nutrition and Health, University of British Columbia, Vancouver, BC, Canada; 4 Faculty of Medicine, Department of Medicine (Neurology), University of British Columbia, Vancouver, BC, Canada; 5 Faculty of Science, Department of Microbiology and Immunology, University of British Columbia, Vancouver, BC, Canada; 6 Michael Smith Laboratories, University of British Columbia, Vancouver, BC, Canada; 7 Department of Pathology and Laboratory Medicine, University of British Columbia, Djavad Mowafaghian Centre for Brain Health, Vancouver, BC, Canada; 8 Faculty of Medicine, Department of Biochemistry and Molecular Biology, University of British Columbia, Vancouver, BC, Canada

**Keywords:** Parkinson’s disease, gut microbiome, inflammatory biomarkers, ketogenic diet, Mediterranean diet, gut-brain axis

## Abstract

Ketogenic diets have been proposed as potential neuroprotective interventions for Parkinson’s disease (PD), but concerns remain regarding their effects on the gut microbiome and gut-brain-immune axis. We conducted a randomized crossover trial comparing 8-week Mediterranean-ketogenic (MeDi-KD) versus medium-chain triglyceride-supplemented Mediterranean (MeDi-MCT) diets, separated by an 8-week washout (NCT05469997). Primary outcomes included changes in gut microbiome taxonomic and functional profiles, fecal short-chain fatty acid (SCFA) production, and biomarkers of intestinal permeability and inflammation. Neither intervention significantly altered microbiome diversity or functional gene abundance, established features of PD-associated dysbiosis, nor fecal or plasma biomarkers of intestinal permeability (zonulin) or inflammation (calprotectin). The MeDi-KD modestly reduced total fecal SCFAs, while both interventions increased the relative abundance of *Roseburia hominis*, and the MeDi-MCT was associated with decreases in plasma IL-1β and IL-8. No severe adverse events were reported, supporting further investigation of MeDi-KD strategies in larger, longer-duration PD trials.

## Highlights

• Dietary and supplement-based Mediterranean-ketogenic diets appear safe in patients with PD
• Modest anti-inflammatory effect of MCT-supplemented Mediterranean diet in PD
• Neither Mediterranean-ketogenic approach markedly alters the gut microbiome in PD

## Teaser

Diet; Interventions; Microbial genomics

## Introduction

Parkinson’s disease (PD) is the second most common neurodegenerative disease and is projected to affect 25 million globally by 2050.^1^ PD motor symptoms are primarily attributed to neurotoxic alpha-synuclein accumulation and dopaminergic neuron loss in the substantia nigra pars compacta,^2^^,^^3^ the latter of which serves as the target of mainstay PD pharmacotherapies. However, dopaminergic medications have limited efficacy in addressing the many non-motor symptoms of PD and have failed to demonstrate a disease-modifying effect. As such, increasing attention has been paid to adjunctive non-pharmacologic approaches targeting pathogenic processes thought to occur upstream of dopaminergic neurodegeneration, including disturbances of the microbiota-gut-brain axis, and neuronal bioenergetic failure.^4^

Gastrointestinal symptoms are among the most common non-motor manifestations of PD, often occurring years to decades before motor symptom onset. These observations, coupled with evidence of gut dysbiosis, gut inflammation and enteric nervous system alpha-synuclein deposits in people with PD (PwP), have led to suggestions of a gastrointestinal origin of PD in at least a subset of patients.^5^ Gut dysbiosis in PD is characterized by a decrease in producers of short-chain fatty acids (SCFAs), which feed intestinal epithelial cells, and a concomitant increase in mucin degraders such as *Akkermansia muciniphila.*^6^^,^^7^ In the setting of PD, these shifts in gut microbiome composition are thought to compromise gut barrier integrity, leading to the penetration of lipopolysaccharides (LPS) and release of proinflammatory cytokines into the bloodstream.^7^^,^^8^^,^^9^^,^^10^^,^^11^ Of the SCFAs, butyrate has the best-established role in regulating mucosal barrier integrity and gut inflammation^12^^,^^13^ with evidence for its ability to alleviate LPS-induced neuroinflammation and brain oxido-nitrosative stress.^14^ Microbiome-targeted therapies in the form of probiotics,^15^ fecal matter transplantation,^16^ and fiber-rich Mediterranean diets^17^^,^^18^ may help to correct this sequence and have shown promise for alleviating non-motor,^17^^,^^18^ and possibly motor,^19^^,^^20^ symptoms of PD.

Whereas the Mediterranean diet is believed to promote gut health, ketogenic diets (KDs) have alternatively been proposed for their putative neuroprotective effects in PD. Ketone bodies (KBs) can serve as an alternative fuel source to glucose in bioenergetically compromised neurons,^21^^,^^22^^,^^23^ specifically by bypassing disease-associated deficits in complex I function.^24^^,^^25^^,^^26^ KBs have also been shown to reduce oxidative stress^27^ and may correct PD-associated dysfunction in mitochondrial biogenesis pathways, such as that regulated by PGC1-α.^28^^,^^29^^,^^30^^,^^31^^,^^32^^,^^33^^,^^34^ Supply of KBs to the brain may be increased through adherence to a high-fat, adequate-protein, low-carbohydrate KD, or through consumption of ketogenic supplements such as medium-chain triglyceride (MCT) oil.^35^ However, concerns have been raised that KDs may exacerbate PD-associated gut dysbiosis and intestinal permeability by decreasing bacterial SCFA synthesis and increasing the relative abundance of *A. muciniphila.*^36^^,^^37^

Preliminary work in non-PD populations suggests that a combination of ketogenic and Mediterranean dietary principles may serve to neutralize the putative adverse effects of KDs on the gut microbiome.^38^ To test this hypothesis in PwP, we designed a 6-month phase II crossover study of two 8-week Mediterranean-ketogenic interventions: a high-fat, low-carbohydrate Mediterranean-ketogenic diet (MeDi-KD) and a standard Mediterranean diet supplemented with MCT oil (MeDi-MCT), separated by an 8-week washout. As primary outcomes, we investigated the effects of both interventions on gut microbiome taxonomic and functional profiles, SCFA production, biomarkers of intestinal barrier integrity, and intestinal and systemic inflammation. In this report, we further relate these outcomes to secondary measures of participant adherence, bowel habits, and clinical symptoms.

## Results

### Baseline characteristics were balanced between randomization groups

Table 1 provides an overview of all clinical assessments and questionnaires administered to research participants. Baseline characteristics of the 52 randomized participants have been reported previously^39^ and are found in Table 2. Median age and disease duration were 68 (IQR: 63, 74) and 7 (IQR: 4, 10) years, respectively, with 16 (31%) participants being female.

**Table 1:** Clinical assessments and questionnaires

Questionnaire	Purpose
Movement disorder society-unified Parkinson’s disease rating scale (MDS-UPDRS)	PD severity (Goetz et al.^44^)
Fatigue severity scale (FSS)	measuring fatigue in patients with PD (Herlofson and Larsen,^164^^,^^165^)
Starkstein apathy scale (SAS)	measuring apathy in patients with PD (Starkstein et al.)^48^
Beck depression inventory II (BDI-II)	assessing symptoms of depression (Beck, Steer and Brown^166^)
Parkinson anxiety scale (PAS)	assessing anxiety in patients with PD (Leentjens et al.^46^)
Parkinson’s disease questionnaire-39 (PDQ-39)	assessing quality of life in PD patients (Jenkinson et al.^167^)
Physical activity scale for individuals with physical disabilities (PASIPD)	assessing the performance of physical activities (Washburn et al.^168^)
Bristol stool scale	assessing stool consistency (Lewis and Heaton^169^)
Rome III classification	constipation and irritable bowel symptoms (Drossman and Dumitrascu^170^)
Patient global impression of change (PGIC)	assessing perception of change due to the interventions (Fischer et al.^171^)
World Health Organization quality of life questionnaire (WHO QoL)	general quality of life (Hendred and Foster^172^)
The Canadian version of the diet history questionnaire II (DHQ-II)	dietary habits prior to beginning of the study (Canadian Diet History Questionnaire II^173^)
Montreal cognitive assessment (MoCA)	assessment of cognitive function (Nasreddine et al.^174^)
Parkinson’s disease cognitive rating scale (PD-CRS)	assessment of cognitive function (Pagonabarraga et al.^175^)
Parkinson’s disease cognitive functional rating scale (PD-CFRS)	assessment of functional impact of cognitive impairment (Kulisevsky et al.^176^)

**Table 2:** Baseline characteristics stratified by initial intervention

Characteristic	Overall *N* = 52^a^	MeDi-KD *N* = 27^a^	MeDi-MCT *N* = 25^a^	*p* value^b^
Disease duration	7 (4, 10)	8 (4, 11)	7 (4, 10)	0.5
Age	68 (63, 74)	71 (63, 75)	67 (62, 69)	0.10
Sex				0.4
Female	16 (31%)	7 (26%)	9 (36%)	
Male	36 (69%)	20 (74%)	16 (64%)	
Ethnicity				0.2
Black/African	1 (2.0%)	0 (0%)	1 (4.2%)	
Caucasian	41 (84%)	22 (88%)	19 (79%)	
East Asian	4 (8.2%)	3 (12%)	1 (4.2%)	
South Asian	3 (6.1%)	0 (0%)	3 (13%)	
Years education	18 (16, 20)	17 (16, 20)	18 (17, 20)	0.6
BMI	25.8 (22.3, 28.7)	26.7 (22.6, 28.5)	24.9 (22.3, 28.7)	0.6
Levodopa equivalent dose (mg/day)^c^	625 (375, 950)	663 (550, 910)	500 (10, 1,130)	0.3
Hoehn and Yahr Stage				0.5
0	0 (0%)	0 (0%)	0 (0%)	
I	7 (13%)	5 (19%)	2 (8.0%)	
II	41 (79%)	20 (74%)	21 (84%)	
III	4 (7.7%)	2 (7.4%)	2 (8.0%)	
Employed	10 (20%)	5 (20%)	5 (21%)	>0.9
Partnered	37 (76%)	18 (72%)	19 (79%)	0.6
Married	34 (67%)	17 (65%)	17 (68%)	0.8
MEDAS score	5.00 (4.00, 6.00)	5.00 (4.00, 6.00)	6.00 (4.00, 7.00)	0.6
MDS-UPDRS Part I	10.5 (6.5, 14.0)	11.0 (8.0, 14.0)	10.0 (6.0, 13.0)	0.5
MDS-UPDRS Part II	8.0 (5.5, 12.0)	8.0 (6.0, 11.0)	9.0 (4.0, 13.0)	0.7
MDS-UPDRS Part III	26 (18, 36)	25 (16, 34)	26 (19, 37)	0.5
MDS-UPDRS Part IV	4.0 (0.0, 7.0)	4.0 (0.0, 7.0)	4.0 (0.0, 7.0)	0.9
MoCA total score	27.00 (25.00, 29.00)	27.00 (26.00, 29.00)	26.50 (25.00, 29.00)	0.5
PD-CRS total score	91 (71, 106)	92 (72, 106)	87 (70, 105)	0.8
Constipation (Rome III criteria)	12 (23%)	5 (19%)	7 (28%)	0.4
MDS-UPDRS constipation item (1.11)				>0.9
Normal	23 (45%)	11 (41%)	12 (50%)	
Slight	20 (39%)	11 (41%)	9 (38%)	
Mild	7 (14%)	4 (15%)	3 (13%)	
Moderate	0 (0%)	0 (0%)	0 (0%)	
Severe	1 (2.0%)	1 (3.7%)	0 (0%)	
Fatigue (FSS ≥ 37)	25 (53%)	13 (54%)	12 (52%)	0.9
Anxiety (PAS > 13)	19 (40%)	10 (42%)	9 (39%)	0.9
Depression (BDI-II > 13)	13 (27%)	7 (28%)	6 (26%)	0.9
Apathy (SAS ≥ 14)	20 (43%)	10 (42%)	10 (45%)	0.6

a Median (Q1, Q3); *n* (%); analyses were performed using available-case data.

b Wilcoxon rank-sum test; Pearson’s chi-squared test; Fisher’s exact test.

c As calculated using the equation proposed by Tomlinson et al.^177^

Forty-one (79%) participants were Hoehn and Yahr Stage 2, with median MDS-UPDRS Parts I–IV scores of 10.5 (IQR: 6.5, 14), 8.0 (IQR: 5.5, 12), 26 (IQR: 18, 36), and 4 (IQR: 0, 7.0), respectively. The median levodopa equivalent dose was 625 (IQR: 375, 950) mg/day, and the median BMI was 25.8 (IQR: 22.3, 28.7). While median MoCA and PD-CRS total scores fell within the normal ranges, 14 (27%) of participants screened positive for PD-mild cognitive impairment (PD-MCI) using the MoCA (total score: 21–25),^40^ while 21 (40%) demonstrated impaired cognition using the PD-CRS (total score ≤ 85 points).^41^ Twelve (23%) of the cohort experienced clinically significant constipation prior to starting the trial according to the ROME III module criteria,^42^^,^^43^ and 28 (55%) self-reported constipation symptoms in response to MDS-UPDRS Item 1.11.^44^ Clinically significant fatigue (fatigue severity scale [FSS] ≥ 37),^45^ anxiety (Parkinson anxiety scale [PAS] > 13),^46^ depression (Beck depression inventory II [BDI-II] > 13),^47^ and apathy (Starkstein apathy scale [SAS] ≥ 14)^48^^,^^49^ were present in 25 (53%), 19 (40%), 13 (27%), and 20 (43%) participants, respectively. Baseline characteristics did not differ significantly between the two randomization groups.

### Intervention adherence and bowel habits did not vary significantly between interventions

As described previously, participants reported high rates of adherence to both interventions, with 92% and 73% reporting that they followed the diets every day or deviated from meal plans at most several times a month for the MeDi-MCT and MeDi-KD, respectively.^39^ Frequency of bowel movements (OR = 1.03; 95% CI: 0.82–1.28), Bristol scores (OR = 1.16; 95% CI: 0.47–2.85) and frequency of laxative use (OR = 1.03; 95% CI: 0.80–1.33) did not differ significantly between the two diets, nor did they show consistent temporal trends within each intervention phase (Figures S1A–S1C). There were no significant intervention-related changes in self-reported constipation symptoms using MDS-UPDRS Item 1.11 with either the MeDi-KD (r = 0.00, *p* = 0.76) or the MeDi-MCT (r = 0.00, *p* = 0.96). However, of the symptom domains assessed by the Patient Global Impression of Change (PGIC) scale, the most frequently reported improvements were in bowel function (Table S2), which were associated with improvements in both patient-reported overall wellbeing (*p* = 0.009), as well as quality of life as measured by the PDQ-39 (*p* = 0.022). Forty-four percent of participants reported improvements in bowel symptoms on the MeDi-KD versus 54% on the MeDi-MCT according to the PGIC, as compared with 24% and 22% reporting worsening of bowel symptoms with each intervention, respectively (*p* = 0.7).

### Modest reductions in plasma IL-1β and IL-8 were seen following the MeDi-MCT

Experimental plasma sample inter-well CVs were under 15% for all analytes, and all samples were over the limit of detection. Mean baseline and post-intervention levels of each peripheral inflammatory biomarker assayed are showcased in Table S3. We observed a statistically significant decrease in plasma IL-1β (*p* = 0.014; q = 0.043) from a baseline of 0.15 (SD: 0.10) to 0.12 (SD: 0.10) pg/mL, and in IL-8 (*p* = 0.003; q = 0.019) from a baseline of 6.17 (SD: 6.00) to 5.26 (SD: 6.98) pg/mL following the MeDi-MCT intervention (Figure 1). However, neither of these within-intervention changes exceeded the respective RCVs of 0.15 pg/mL for IL-1β and 5.25 pg/mL for IL-8. The MeDi-KD intervention did not have a significant effect on any of the measured inflammatory biomarker levels (*p* > 0.05; Table S4), and there were no significant effects of intervention sequence.

**Figure 1:**
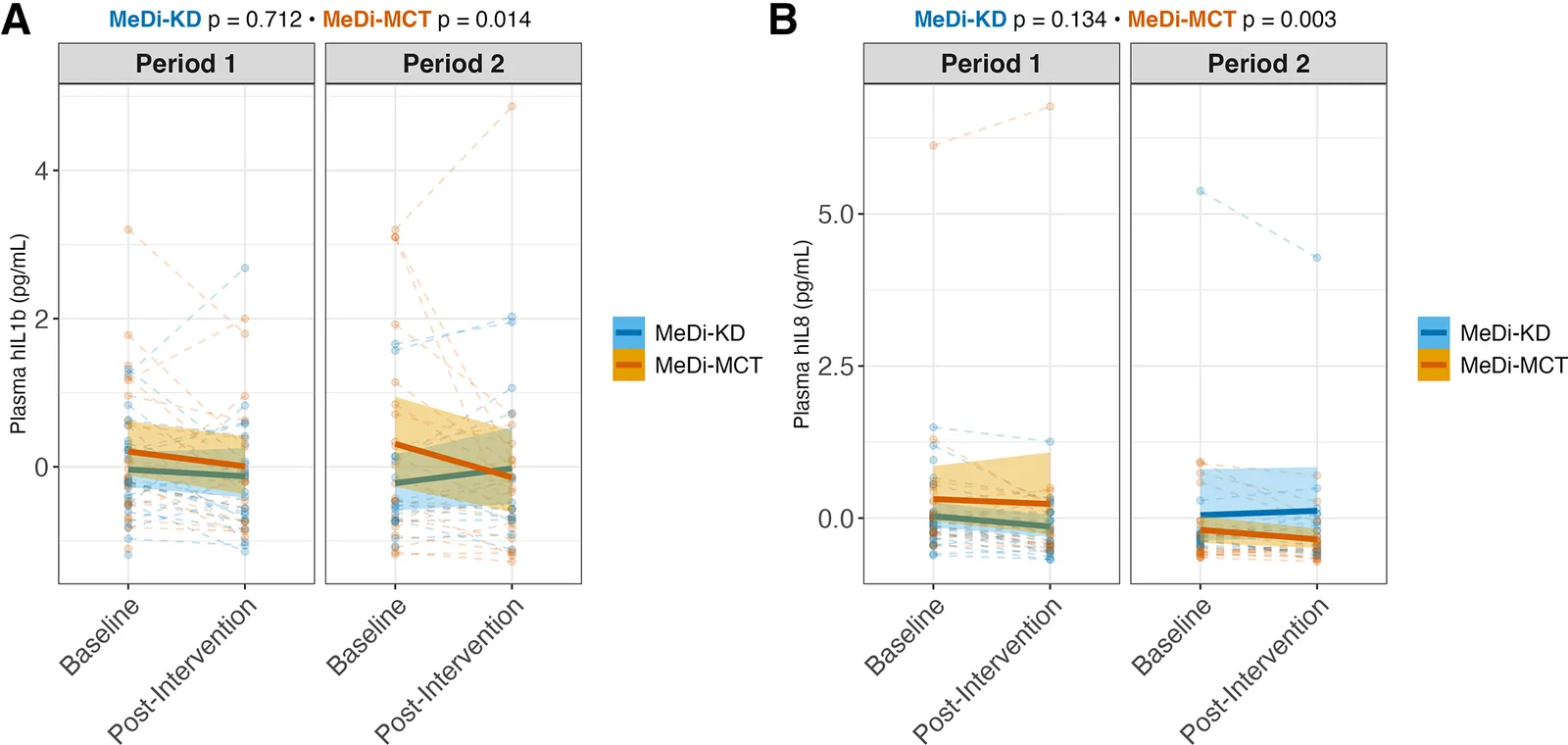
Modest decreases in a susbet of plasma inflammatory biomarkers observed in response to the MeDi-MCT intervention (A and B) Plasma levels of IL-1β (A) and IL-8 (B) decreased in response to the MeDi-MCT, but not the MeDi-KD, intervention. Dashed lines represent individual participant trajectories. Solid points denote geometric means for each intervention at baseline and post-intervention, with error bars indicating 95% confidence intervals. Biomarker values are plotted on a log_10_ scale due to right-skewed distributions. Reported *p* values correspond to within-intervention baseline–to–post comparisons (Wilcoxon signed-rank test).

### Overall gut microbiome composition was unchanged across interventions and timepoints

At baseline, bacterial communities clustered into a single community type at the genus level.^50^ As shown in Figure 2, the genus *Bacteroides* was most dominant across interventions and timepoints. *Phocaeicola* and *Faecalibacterium* also represented key genera within the core gut microbiota of our cohort samples.

**Figure 2:**
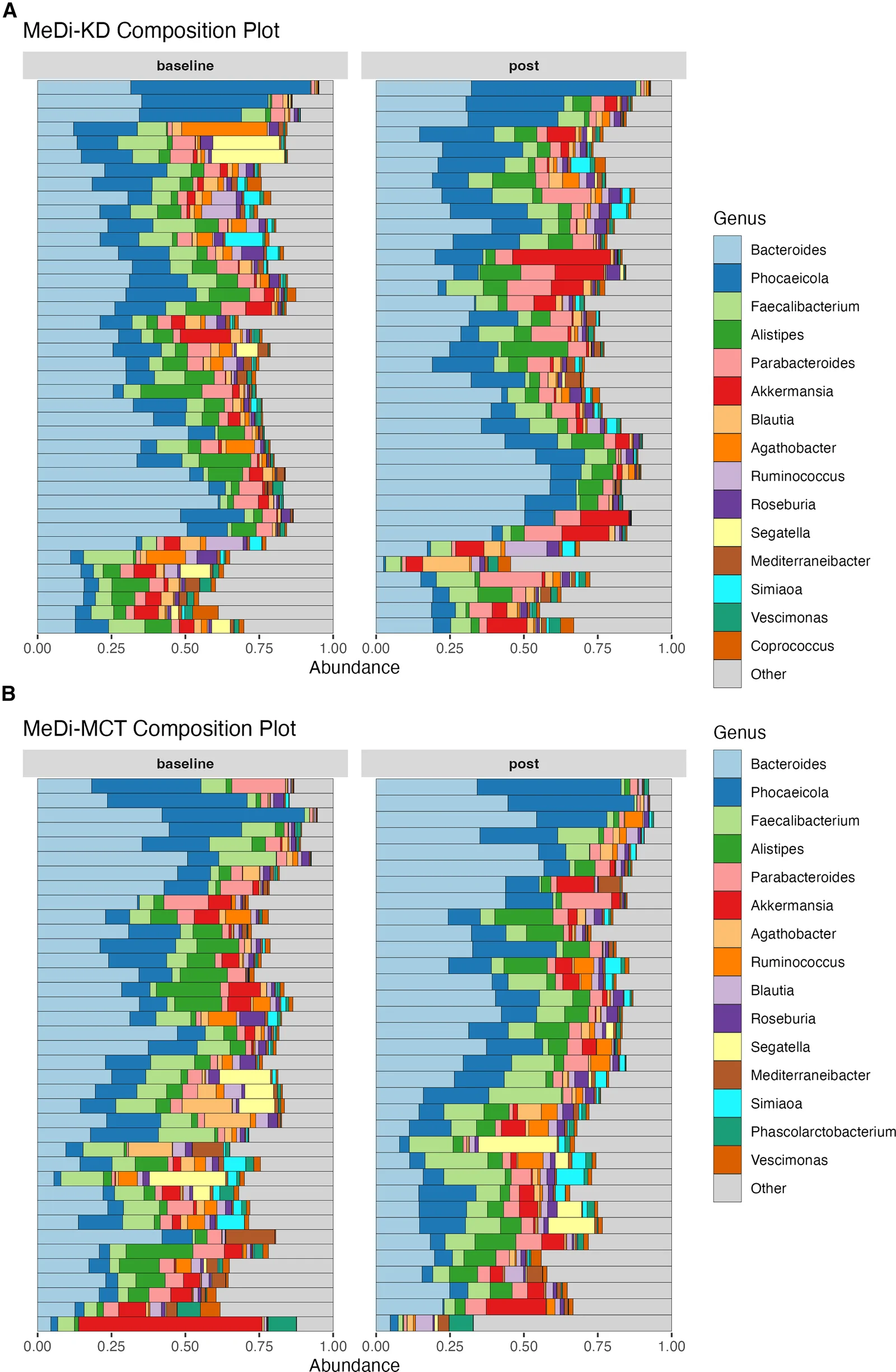
Relative abundance of the top 15 most abundant genera before and after each intervention illustrates limited shifts in community composition between timepoints within individual participants, and a lack of consistent diet-induced shifts across participants

### Neither the MeDi-KD nor MeDi-MCT produced significant changes in microbiome alpha diversity

No significant changes in alpha diversity metrics were observed in response to either diet (*p* > 0.05) (Figure 3; Table S5). At baseline, plasma IL-6 levels were inversely associated with observed richness (*p* = 0.016), Chao1 index (*p* = 0.022), and Shannon Index (*p* = 0.034). Serum total cholesterol levels were inversely associated with Chao1 (*p* = 0.048), Shannon (*p* = 0.023), and Simpson (*p* = 0.029) indices, as were serum LDL cholesterol levels (Chao1: *p* = 0.027; Shannon: *p* = 0.012; Simpson: *p* = 0.011). Baseline stool zonulin levels were inversely associated with observed richness (*p* = 0.035) and Chao1 index (*p* = 0.020). Percent energy intake from saturated fats at baseline was negatively associated with Shannon (*p* = 0.031) and Simpson (*p* = 0.006) indices (Tables S6A–S6E).

**Figure 3:**
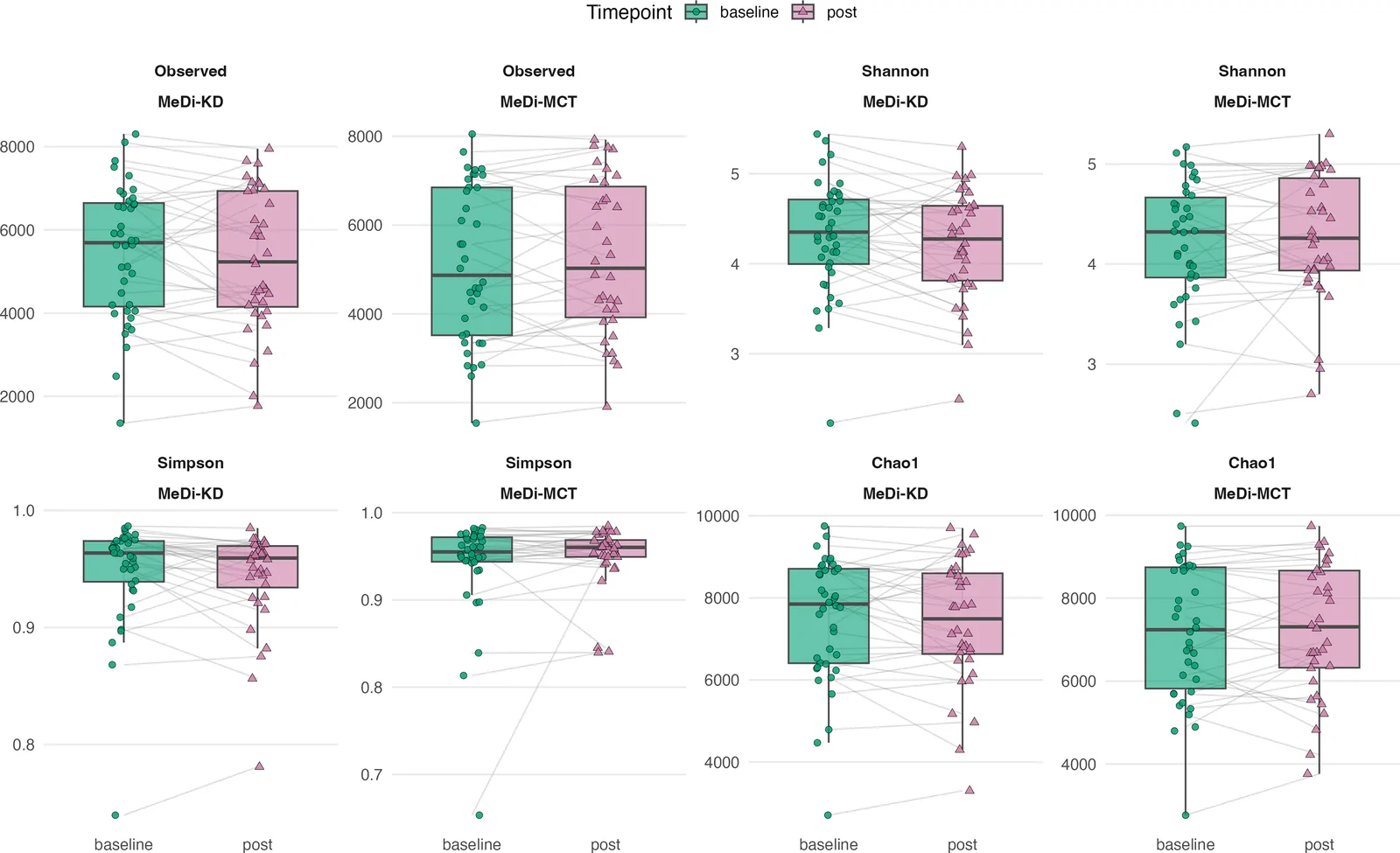
Observed species richness, Shannon, Simpson, and Chao1 Indices remained stable between baseline and follow-up timepoints for both interventions Boxes show median and IQR; whiskers extend to 1.5× IQR.

### Overall community composition remained stable across interventions and timepoints

There were no significant effects of intervention (R^2^ = 0.006; *p* = 0.35), timepoint (R^2^ = 0.005; *p* = 0.50), or their interaction (R^2^ = 0.006; *p* = 0.11) on beta-diversity as assessed by PERMANOVA (Figure 4), nor were any significant changes in average community composition observed between baseline measurements (R^2^ = 0.010; *p* = 0.95). Beta diversity did not differ between sample types (R^2^ = 0.008; *p* = 0.16).

**Figure 4:**
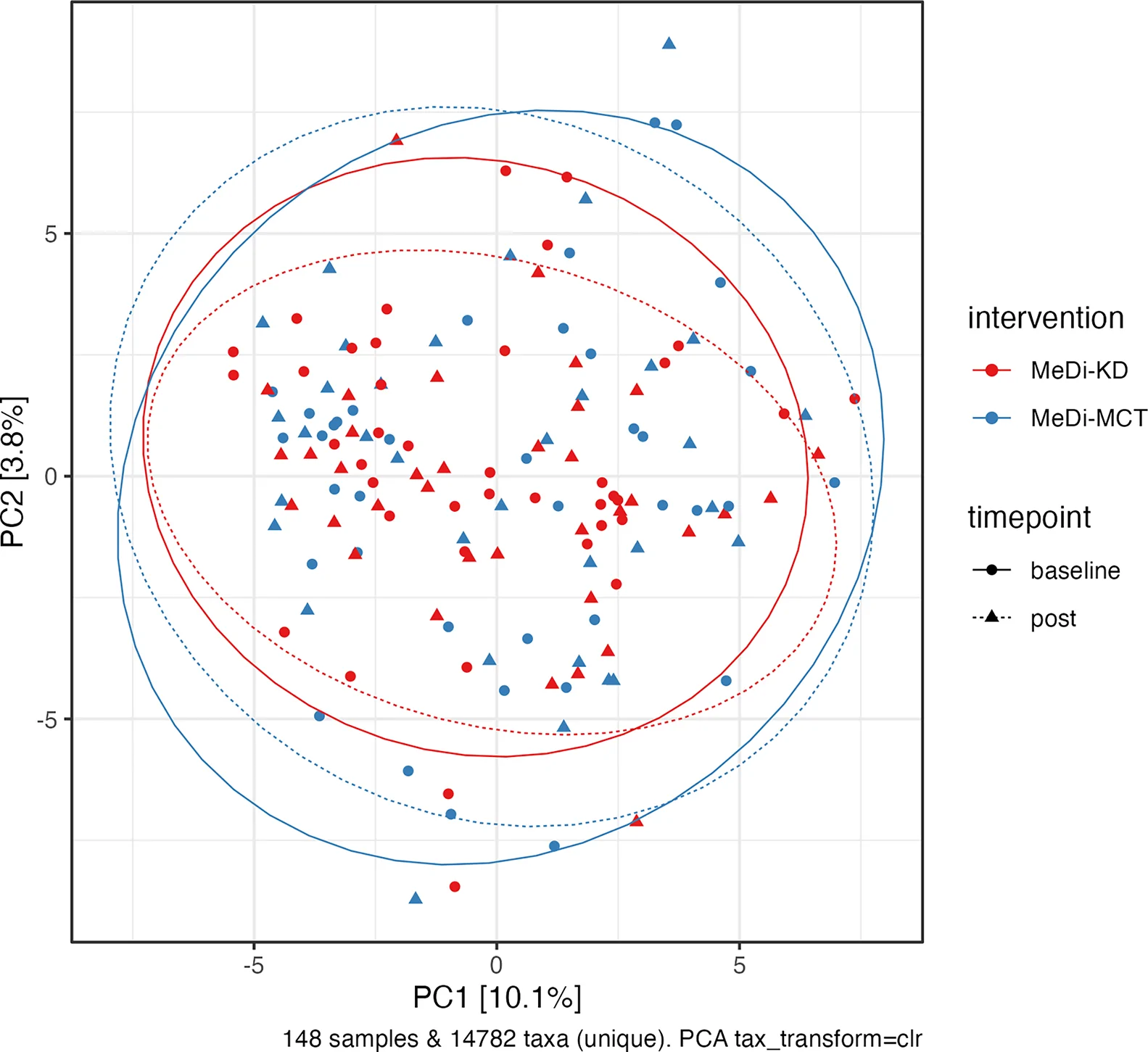
Ordination plot demonstrating no marked shifts in community composition between or within interventions

### Microbiome resistance to intervention-induced change was predicted by higher baseline abundance of Bacteriodaceae but was not related to clinical effects

Microbiome volatility was calculated using repeated sampling before and after each intervention and between baselines. No significant differences in volatility were observed between interventions (*p* = 0.07, Figure 5). Volatility between baselines was significantly greater than within-intervention volatility for both the MeDi-KD (*p* = 0.030) and MeDi-MCT (*p* < 0.001) among participants who provided fecal samples at all four timepoints (*n* = 28) (Figure S2). No significant associations were found between the degree of community composition change and weight loss during the MeDi-KD, nor MeDi-MCT-related changes in MDS-UPDRS Part II or IV, IL-8 or IL-1β (*p* > 0.05).^39^ Inverse associations were observed between the relative abundance of the phylum Bacteroidota (q < 0.001)*,* family Bacteroidaceae (q < 0.001), and constituent genera, *Bacteroides* (q < 0.001) and *Phocaeicola* (q < 0.001), at baseline and the subsequent degree of intervention-related change in microbiome composition.

**Figure 5:**
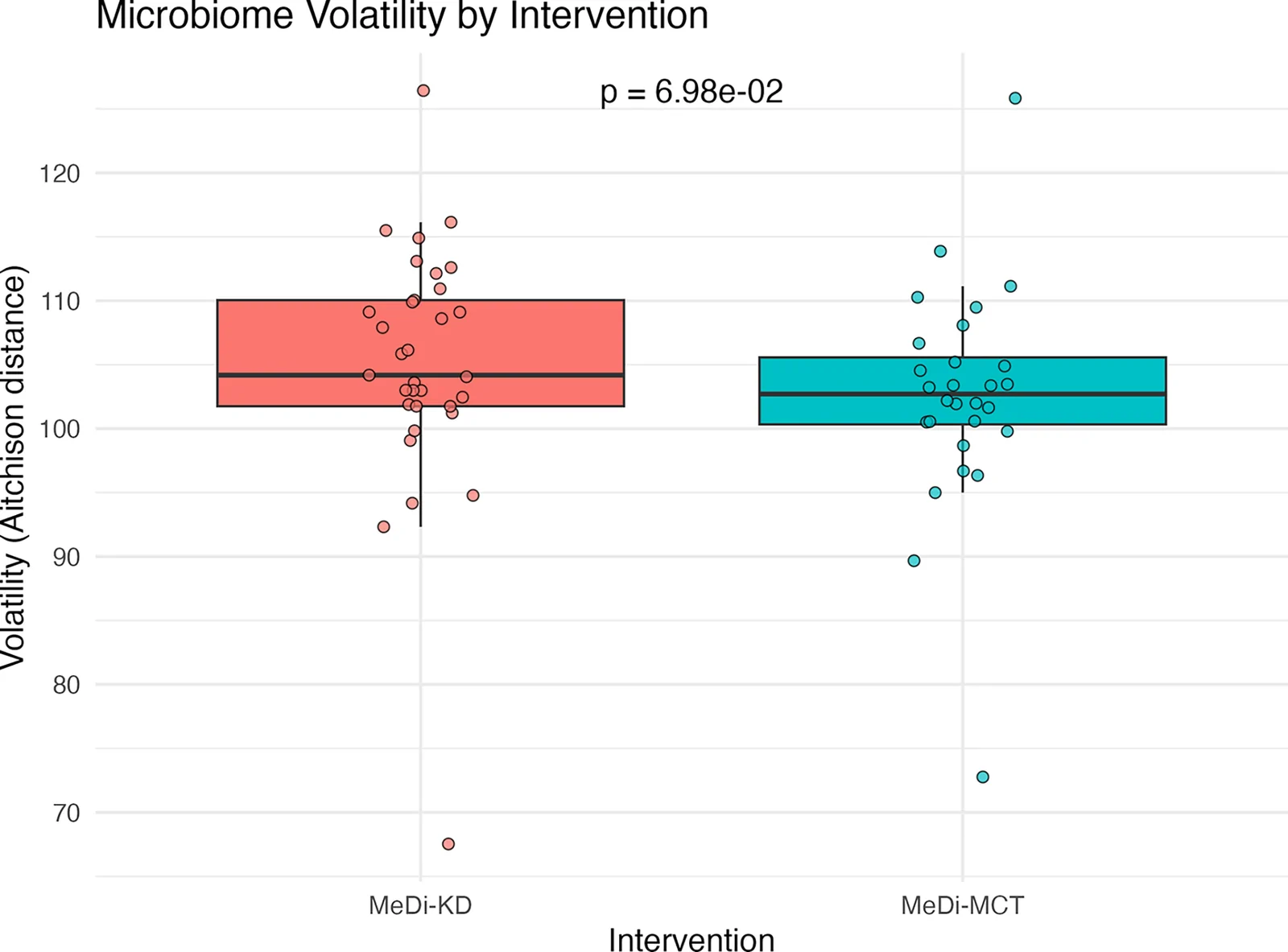
Volatility analysis identified no significant differences in degree of community composition change between the two interventions. The *p* value derives from the paired Wilcoxon signed-rank test comparing within-participant microbiome volatility during the MeDi-KD versus MeDi-MCT intervention (*n* = 33). The boxes show median and IQR; whiskers extend to 1.5× IQR.

### Both the MeDi-MCT and MeDi-KD resulted in significant increases in *Roseburia hominis* relative abundance

While no between-intervention differences in species, genus, family, or phylum abundances were observed, abundance of *Roseburia hominis* increased relative to baseline across the two interventions in paired analysis (q = 0.023) (Figure 6). However, the relative abundance of *R. hominis* was not associated with diet-related changes in fecal SCFAs, circulating IL-1β or IL-8 levels, MDS-UPDRS Parts II/IV scores, or changes in body mass (q > 0.05).

**Figure 6:**
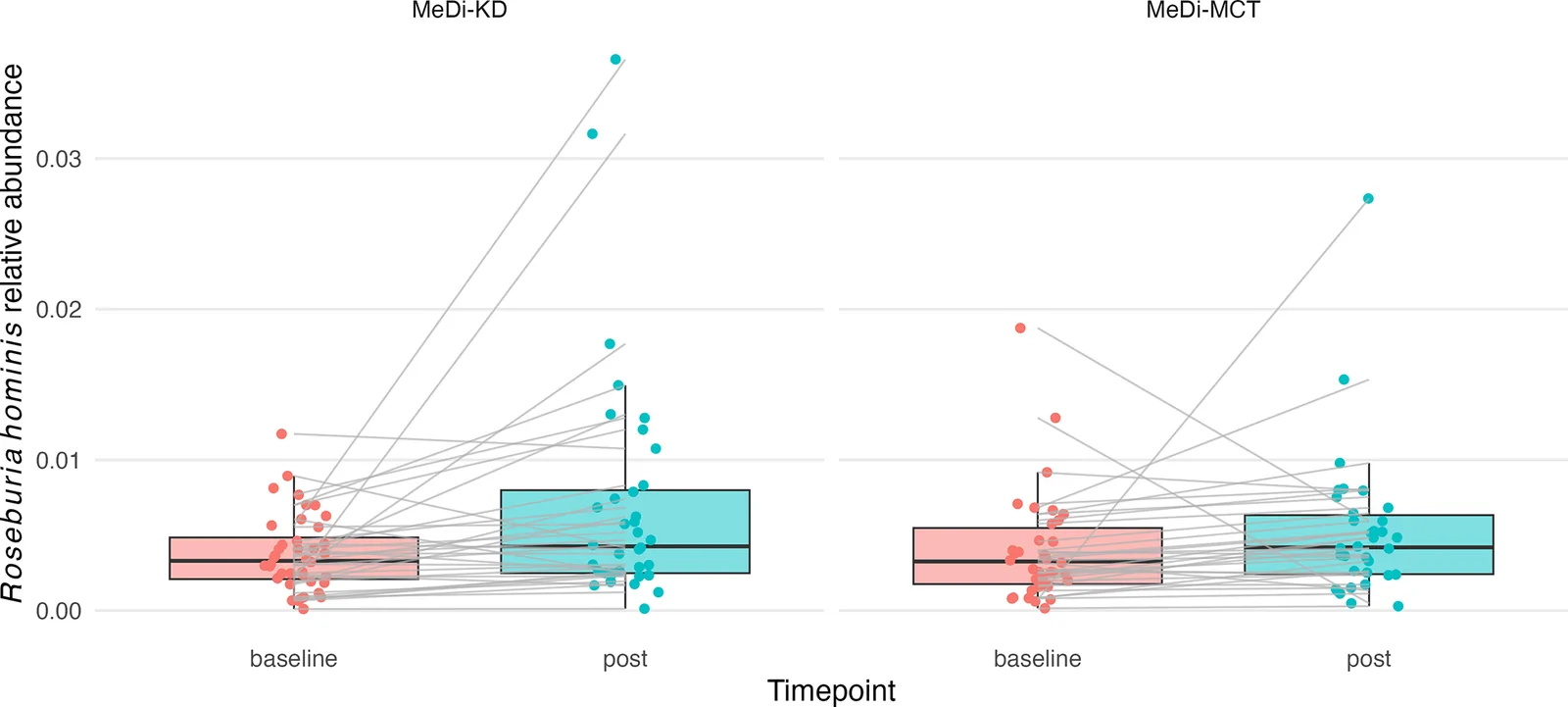
Relative abundance of *Roseburia hominis* increased modestly across both the MeDi-KD and MeDi-MCT (*q* = 0.023) The boxes show median and IQR; whiskers extend to 1.5× IQR.

### Neither intervention significant affected microbiome function

No significant changes in functional beta or alpha diversity metrics (*p* > 0.05; lrl < 0.3), nor relative abundance of individual functional genes (q > 0.05), were observed in response to either intervention.

### No significant between or within-intervention differences were seen in microbiome association networks

After multiple testing corrections, we did not observe any significant differences in global network properties (edge density difference = 0.010 *p* = 0.36; clustering coefficient difference = 0.13, *p* = 0.29; modularity difference = 0.13, *p* = 0.12) nor pairwise microbial associations when comparing baseline to post-intervention samples, nor when comparing MeDi-KD vs. MeDi-MCT samples at the post-intervention timepoint (Figures S3A–S3D).

### Reductions in fecal SCFAs were observed in response to the MeDi-KD, but not the MeDi-MCT, intervention

There were no significant intervention-related changes in concentration of the primary SCFA of interest, fecal butyrate (*p* > 0.05; lrl < 0.3) (Figure 7; Table S4). However, levels of total SCFAs (*p* = 0.039) as well as acetic acid (*p* = 0.041), propionic acid (*p* = 0.022), and isovaleric acid (*p* = 0.012) decreased from baseline following the MeDi-KD by a mean of 11.5% (SD: 41.4%), 10.5% (SD: 44.3%), 10.2% (SD: 54.5%), and 11.9% (SD: 52.2%), respectively (Figure S4). There were no strong correlations between individual or total SCFA levels and individual taxa at the species or genus level nor with any functional genes. Regarding between-intervention comparisons, total SCFAs (*p* = 0.002), acetic acid (*p* = 0.001), butyric acid (*p* = 0.047) (Figure 7), isovaleric acid (*p* = 0.019), and valeric acid (*p* = 0.020) were significantly lower following the MeDi-KD compared with the MeDi-MCT. No significant associations were observed between fecal SCFA concentrations and intervention-related changes in clinical outcomes, plasma inflammatory biomarkers, or community composition (*p* > 0.05). There were no significant relationships between SCFA levels and intervention sequence.

**Figure 7:**
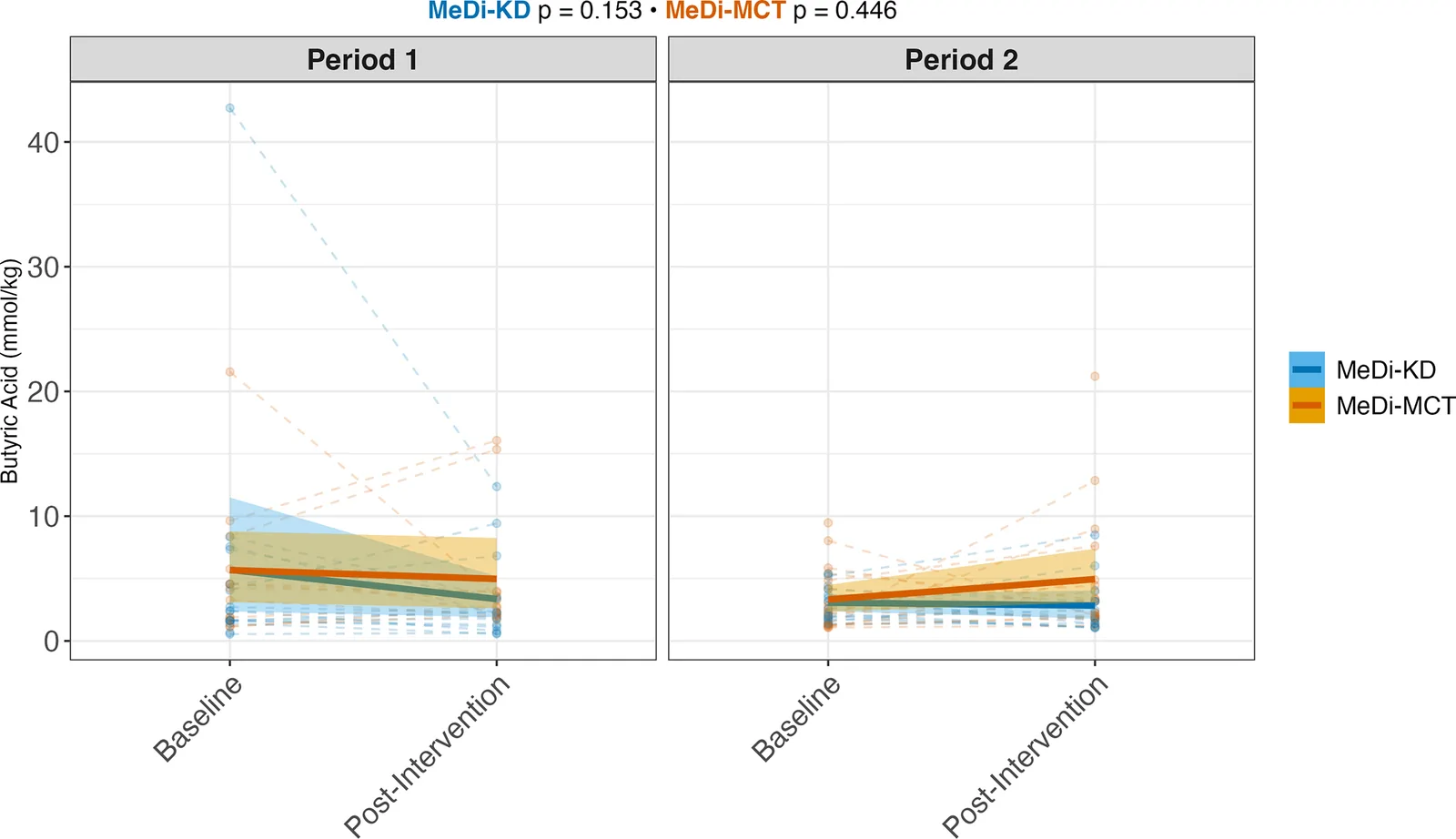
Intervention-related differences in changes from baseline in fecal butyric acid levels, separated by study period Dashed lines represent individual participant trajectories. Error bars indicate 95% confidence intervals.

### Fecal and serum zonulin and calprotectin levels were not affected by either intervention

There were no significant within or between-intervention changes in serum or stool zonulin or calprotectin (*p* > 0.05; r < |0.2|) (Figure S5; Table S4).

## Discussion

The aim of this study was to assess whether combining ketogenic nutritional interventions with a fiber-rich Mediterranean diet could avert the putative undesirable effects of standard KDs on the gut microbiota-immune-brain axis in the setting of PD. To this end, we measured and compared changes from baseline in stool microbiome composition and function, fecal SCFA concentrations, stool and serum calprotectin, stool and serum zonulin, and plasma concentrations of systemic inflammatory biomarkers in response to MeDi-KD and MeDi-MCT interventions. Neither intervention resulted in significant changes in community-level taxonomic profiles, alpha diversity, functional gene or SEED subsystem abundances, taxonomic features characteristic of PD gut dysbiosis, nor any increases in any of the measured peripheral or fecal biomarkers of gut or systemic inflammation. Despite the lack of observed community-level taxonomic changes, both interventions were associated with increases in the butyrate-producing species *R. hominis.* Concurrent reductions in plasma levels of the pro-inflammatory cytokines, IL-1β and IL-8, following the MeDi-MCT suggest a potential anti-inflammatory effect of this diet. Although the effects of each diet on gastrointestinal symptoms were variable and therefore did not reach statistical significance, improvements in self-reported bowel symptoms in a subset of participants were associated with improvements in overall quality of life. The relatively low rates of baseline constipation in our cohort likely limited the power of our study to detect significant intervention-related improvements in bowel function. While these results are overall reassuring regarding the short-term safety of both diets in PwP from a gastrointestinal and immune perspective, reductions in several fecal SCFA levels following adherence to the MeDi-KD warrant further investigation.

Indeed, reports of decreased fecal SCFA content in adults adhering to classical KDs for the treatment of both epilepsy and obesity were one of the key motivating concerns behind our trial.^51^^,^^52^^,^^53^ Fecal SCFAs derived from bacterial fiber (non-digestible carbohydrate) fermentation are decreased in PwP relative to healthy controls, with levels of acetate, propionate and butyrate inversely correlated to measures of disease severity.^54^ These straight-chain SCFAs are thought to help protect against PD-associated intestinal barrier compromise and downstream inflammation by serving as a fuel source for colonocytes, promoting tight junction protein expression, and stimulating mucus production.^55^^,^^56^^,^^57^ Butyrate administration has additionally been shown to protect against PD-related peripheral and central inflammation, oxidative and oxido-nitrosative stress via HDAC inhibition,^58^ and the JAK2/STAT3 signaling pathway.^13^^,^^14^^,^^59^^,^^60^ To mitigate against unwanted SCFA reductions, we pursued modified ketogenic interventions in our MeDi-KD and MeDi-MCT diets, in which we encouraged increased consumption of fiber-rich foods than is typical of classical KDs.^38^

Our fiber-optimized MeDi-KD intervention resulted in more subtle decreases in total fecal SCFAs and the fiber fermentation products, acetate and propionate of 10–12%, relative to the more dramatic decreases of 30%–80% seen with 4–6 weeks classical KDs.^51^^,^^52^ The magnitude of the observed changes may be consistent with random biological variation,^61^^,^^62^ and we did not witness any significant changes in fecal butyrate. Nevertheless, observed decreases in fecal acetate and propionate are of potential clinical relevance on their own: in PD mouse models, gut microbiome-derived propionate has been shown to improve both intestinal epithelial barrier integrity and motor function,^63^^,^^64^ while anti-neuroinflammatory effects have been proposed for both gut-derived acetate and propionate.^55^^,^^65^^,^^66^ Unlike acetic, propionic, and butyric acid whose physiologic effects have been studied extensively, the implications of our observed MeDi-KD-associated decrease in fecal isovaleric acid are less clear.^67^^,^^68^^,^^69^^,^^70^ This branched-chain fatty acid (BCFA), like isobutyric acid, is generated by fermentation of branched-chain amino acids (valine, leucine, and isoleucine), mainly by *Bacteroides* and *Clostridium* species in the human large intestine.^67^ Products of protein fermentation are believed to exert adverse effects on the colon, and fecal BCFA content has correspondingly been linked to aging, obesity, and irritable bowel syndrome.^67^^,^^69^^,^^71^ Fecal BCFA content increases in response to high protein diets, while negatively correlating with dietary fiber intake.^67^ The focus of our MeDi-KD intervention on high unsaturated fat intake and sufficient fiber-rich carbohydrates, with less emphasis on animal-based proteins, may help to explain reductions in fecal BCFAs, along with the less dramatic decreases we saw in carbohydrate-derived straight-chain SCFAs than have been reported for classical KDs.^51^^,^^52^^,^^72^ Taken together, it appears that the implementation of ketogenic principles in the context of a Mediterranean eating pattern attenuated, without entirely eliminating, unfavorable effects of traditional KDs on intestinal SCFA production, in keeping with previous studies in non-PD populations.^38^ The comparative advantages of the MeDi-KD versus the MeDi-MCT in this regard are in need of further study, as the lack of within-intervention MeDi-MCT-induced changes in fecal SCFA levels in our trial resulted in significantly lower concentrations of both beneficial straight-chain (acetic and propionic) and potentially harmful branched-chain (isovaleric and isobutyric) acids following the MeDi-KD relative to the MeDi-MCT.

Consistent with our comparatively modest observed diet-induced decreases in SCFAs,^55^^,^^73^^,^^74^ the overall stability of serum and fecal zonulin suggests that the combination of a ketogenic regimen with a MeDi may abridge previously reported KD-induced compromises of intestinal barrier function.^52^^,^^75^^,^^76^ Zonulin is an acute phase reactant which induces intestinal tight junction disassembly, for which both stool and serum levels have historically been measured as surrogate markers of intestinal permeability.^77^^,^^78^^,^^79^ The lack of apparent diet-induced change in intestinal permeability could relate to the lack of an *A. muciniphila* “bloom” in our study.^36^^,^^80^^,^^81^^,^^82^ Indeed, although *A. muciniphila* is generally considered to improve gut barrier function and confer beneficial effects on human health, opposite effects can be seen in particular disease and dietary contexts.^80^ In healthy hosts consuming a nutritionally complete diet, mucus degradation by *A. muciniphila* appears to stimulate compensatory mucin synthesis, thereby strengthening barrier defenses.^83^ In contrast, under conditions of dietary fiber inadequacy,^80^^,^^84^ or in disease states such as PD wherein host compensatory responses may be inherently blunted,^83^^,^^85^ accelerated mucin degradation by an expanded population of *A. muciniphila* may overwhelm host compensatory responses and thereby increase intestinal permeability. The fact that neither of the fiber-rich KDs in our trial appears to have elicited such a response indicates their potential superior safety profile over traditional KDs with respect to intestinal barrier function in PwP.

Important caveats must be noted with respect to the reliance on zonulin ELISA assays as our indicator of intestinal barrier integrity. Several studies have raised concerns regarding the specificity of commercially available human zonulin ELISA kits, with reported high levels of cross-reactivity to the serum glycoprotein, properdin, and other “zonulin family peptides.”^86^^,^^87^^,^^88^^,^^89^ Inconsistent concordance with other measures of intestinal permeability and gastrointestinal disease activity has further fueled concern regarding circulating zonulin’s validity as a clinically relevant biomarker.^77^^,^^79^^,^^86^^,^^89^^,^^90^^,^^91^^,^^92^ While measurement of fecal zonulin has been proposed as a more specific marker of intestinal barrier dysfunction, with reduced interference by cross-reactive plasma proteins and zonulin family peptides derived from other tissues, validation studies are limited.^91^^,^^93^ Future research will benefit from multimodal assessments of intestinal barrier function to enhance confidence in findings and establish consensus biomarkers.

Similar caution must be exercised in the interpretation of serum and fecal calprotectin levels as measures of gut inflammation, neither of which changed significantly in response to either the MeDi-MCT or MeDi-KD. Although elevated fecal calprotectin levels in PD have been previously reported as evidence of disease-related intestinal inflammation,^10^^,^^56^^,^^57^^,^^94^^,^^95^^,^^96^^,^^97^ their relationship to disease severity is less clear.^98^ Evidence for elevations in serum calprotectin in PD is even fewer,^10^ and the extent to which serum calprotectin represents a biomarker of gut versus systemic inflammation is a matter of scientific debate.^99^

Systemic inflammatory processes may have been better interrogated by measurement of circulating cytokines in our study, with which we saw small statistically significant decreases in plasma levels of the PD-linked proinflammatory cytokines, IL-8 and IL-1β,^100^^,^^101^^,^^102^^,^^103^^,^^104^ in response to the MeDi-MCT. Although these anti-inflammatory effects were within the range of natural temporal variation, they are nevertheless consistent with prior Mediterranean diet research, and may be mediated by signaling properties of constituent flavonoids, polyphenols, omega-3 polyunsaturated fatty acids, monounsaturated fatty acids, or other MeDi-enriched nutrients.^105^^,^^106^^,^^107^^,^^108^ BHB itself has been shown to inhibit NLRP3 inflammasome-stimulated production of IL-1β,^109^^,^^110^ while caprylic acid (one of the medium-chain fatty acids found within MCTs), has been shown to suppress IL-8 transcription and secretion through its actions on the NF-κB pathway.^111^^,^^112^ Direct anti-inflammatory mechanisms of MCTs/MCFAs,^112^^,^^113^^,^^114^^,^^115^^,^^116^ may help to explain the distinct inflammatory responses seen between our MeDi-MCT and MeDi-KD interventions. Our findings are nonetheless encouraging given the absence of undesirable increases in PD-associated circulating pro-inflammatory cytokines, including IL-6, TNFα, IL-10, and IL-12p70,^100^^,^^102^^,^^103^^,^^104^^,^^117^ in response to either of our study interventions.

While we did not find significant changes in microbiome composition or functional genes in association with observed intervention-related changes in SCFA and inflammatory biomarker levels, our small sample size and short-duration interventions likely constrained our ability to detect such effects. Although self-rated adherence was high, we have previously described a discrepancy between self-assessed and objective measures of adherence in this trial,^39^ further reducing the number of individuals exposed to the full intensity and/or extent of the interventions. Our target sample size was chosen based on prior clinical studies,^118^ and power calculations using fecal butyrate as a primary outcome,^38^ which may have sufficed to ascertain increases or decreases in univariable continuous biomarkers. However, our ultimate sample of 33 dual intervention completers was likely inadequate to resolve community and many taxon-level changes in the high-dimensional, sparse, zero-inflated multivariate microbiome datasets, wherein statistical power is reduced by the need for distance-based testing and multiple comparisons correction.^119^^,^^120^^,^^121^^,^^122^^,^^123^ Power estimations using community or taxon-level microbiome features as primary endpoints should be considered in sample size calculations for future studies, for which several approaches have been proposed.^119^^,^^121^^,^^124^ Despite our limited power, we did find an increase from baseline in *R. hominis* across both the MeDi-KD and MeDi-MCT. This finding supports a possible net gut-protective effect of our interventions, as *R. hominis* is a known butyrate-producing species depleted in PwP relative to healthy controls,^125^^,^^126^^,^^127^ has been shown to exert anti-inflammatory effects,^125^^,^^128^ and was associated with reduced MDS-UPDRS Part IV scores in our study. Similar increases in *R. hominis* have been observed in previous Mediterranean diet studies^18^^,^^129^ and may represent a key contributor to the putative beneficial effects of Mediterranean diets in PD.^18^

Validation of this observation in longer-term intervention studies is nevertheless needed, as transient diet-induced changes in microbiome activity and composition on the order of days to weeks often fail to translate to stable restructuring of the core gut microbiome community when examined after several months to years.^120^^,^^122^^,^^130^^,^^131^^,^^132^ The larger variation in gut microbiome community composition we observed over the 16 weeks between baseline measurements, as compared with the 8 weeks between intervention baselines and follow-ups, suggests that community-level compositional changes observed in our interventions were largely consistent with natural variation over time (which would be expected to increase with greater duration between comparisons).^133^^,^^134^ Inter-individual variability in microbiome responsiveness to acute dietary change further constrains our ability to draw conclusions based on short-term intervention studies like that herein.^135^^,^^136^^,^^137^^,^^138^^,^^139^^,^^140^ While we did not find an association between baseline alpha diversity and the degree of intervention-related change in community composition in this study, as has been reported elsewhere,^141^ we did witness a previously described link between baseline *Bacteroides* abundance and resistance to intervention-related community-level change.^142^^,^^143^ Novel methods to characterize “diet-responsive” gut microbiome profiles may help with participant selection and stratification in future trials.^122^^,^^131^^,^^138^^,^^139^

Although detection of intervention-related effects was hindered by study duration, analysis of our baseline data offers some insight into the impacts of long-term habitual diet on the gut-microbiome-immune axis. Our study recapitulated previously reported inverse associations between baseline alpha diversity metrics—considered key markers of gut microbiome health^144^—and proportion of calories derived from saturated fats,^145^ serum LDL and total cholesterol,^146^^,^^147^ stool zonulin,^57^ and the PD-associated circulating proinflammatory cytokine, IL-6.^57^^,^^148^ Although correlational in nature, these observations support the notion that long-term dietary composition may more meaningfully impact aspects of the gut-brain-immune axis relevant to PD than can be elicited with short-term dietary modification alone. For design of future longer-duration interventions, the inverse relationship between saturated fat intake and gut microbiome diversity favors the modified, low-saturated, high-unsaturated fat KD approach deployed in our study, versus standard KDs lacking specific guidance around dietary fat composition.^38^^,^^149^

Despite extensive discussion regarding the potential role of diet-microbiome interactions in PD,^150^^,^^151^ our study represents only the third RCT assessing the effects of a dietary intervention on the gut microbiome,^18^^,^^152^ and the first trial of MeDi-KD diets, in this population. To our knowledge, only two other studies have investigated gut microbiome changes in response to MeDi-KD interventions in non-PD adult cohorts.^38^^,^^153^ In keeping with our findings, neither of these studies reported diet-related changes in microbial alpha or beta diversity.^38^^,^^153^ Notably, ours was the only study of these three to use shallow shotgun metagenomic sequencing as opposed to 16S rRNA gene sequencing, enabling species-level resolution, reduced amplification bias, and direct measurement of functional genes.^154^

### Limitations of the study

This said, heterogeneity in study populations, dietary intervention formulation, analytic methods, and observed bacterial abundance associations illustrate some of the many challenges in comparing dietary impacts on the gut microbiome across studies.^131^ Several other limitations must be considered when interpreting the biological readouts of our study, in particular. The lack of a non-intervention control prevents us from disentangling effects common to both the MeDi-KD and MeDi-MCT interventions from either natural variation over time across measured variables or contextual effects related to clinical trial participation.^155^ With respect to our microbiome results, fecal sampling inevitably captures a biased subset of luminal contents,^156^ leaving open questions as to potential diet-induced changes at the level of the mucosa. Although our washout period of 8 weeks exceeds the general recommendation of 4 weeks or greater for diet-gut microbiome intervention crossover studies, these current recommendations are based on relatively few studies, and minimum washout periods to return microbial communities to their baseline state may vary by individual, disease state, and the degree of departure of intervention from habitual diets.^122^ Standardized diet prescriptions during run-in and washout periods may help to reduce baseline diet as a source of interindividual variation^157^; however, there has yet to be consensus on the optimal length of these intervals, and their duration must be balanced against considerations of participant burden.^120^ Beyond reducing statistical power, missing data introduce a potential source of bias: while analyses using maximum likelihood estimation are considered appropriate for longitudinal repeated-measures data under missing-at-random assumptions, these assumptions may have been plausibly violated if reasons for study dropout (i.e., gastrointestinal intolerance) related to unobserved follow-up gut microbiome or inflammatory biomarker outcomes.^158^^,^^159^^,^^160^ Larger studies would enable sensitivity analyses to assess the robustness of findings under alternate assumptions regarding the origins of missing data.^158^ Finally, the lack of blinded raters for clinical assessments in our study introduced a potential source of observer bias which could be eliminated in a larger-scale efficacy trial, with intervention durations of at least 6 months recommended to allow for detection of neuroprotective effects.^161^^,^^162^

Considering these limitations, the results of our multi-omics analysis nevertheless suggest the short-term safety of a low-carbohydrate, high-fat Mediterranean, and MCT-supplemented Mediterranean diet from a gut health perspective in PwP. Larger and longer-term studies are needed to validate these findings and establish a clinical benefit of these interventions. Alongside emerging precision medicine strategies such as those targeting alpha-synuclein,^163^ nutritional interventions represent promising complimentary approaches to modulate interconnected microbiome, metabolic, and immune components of the gut-brain axis implicated in PD pathogenesis. Future studies integrating metagenomics, metabolomics, host inflammatory profiling, genetic susceptibility markers, and longitudinal clinical phenotyping may help identify patient subgroups most likely to benefit from targeted dietary interventions in PD. Gut biomarkers of inflammation and intestinal permeability should continue to be tracked in such trials for indicators of benefit or harm which may have gone undetected in our cohort.

## Resource availability

### Lead contact

Requests for further information and resources should be directed to and will be fulfilled by the lead contact, Silke Appel-Cresswell (silke.cresswell@ubc.ca).

### Materials availability

This study did not generate new unique reagents.

### Data and code availability

• De-identified human metadata, biomarker, and microbiome sequencing data have been deposited at Borealis Dataverse: https://borealisdata.ca/previewurl.xhtml?token=e328ee49-974d-4089-afec-4224c371f3ca. They are publicly available as of the date of publication.
• Code used to generate the main findings of this manuscript is available in this paper’s supplemental information.
• Any additional information required to reanalyze the data reported in this paper is available from the lead contact upon request.

## Acknowledgments

As the leader of the CFNBI, we would like to acknowledge Dr. Cheryl Wellington’s essential contributions to the peripheral inflammatory biomarker analysis included in this study. We would also like to acknowledge her generosity in enabling us to utilize her lab space and equipment to run our ELISAs. Additionally, we would like to thank Dr. Biljana Jonoska Stojkova and Frances Cheng for their statistical support. The KIM trial was funded by the Weston Family Foundation (Grant Number: F21-04689).

## Author contributions

S.K., M.S.C., A.M.-R., M.A.S., B.B.F., T.R.C., and S.A.-C. were involved in study conceptualization, methodology, and funding acquisition. S.A.-C. supervised the project. K.T., Y.N.W., and A.J.K. were responsible for investigation, data curation, and project administration. K.T. conducted the statistical analysis. K.T., Y.N.W., A.J.K., S.L.S., and S.A.-C. had unrestricted access to all data. S.L.S., T.F.M., M.Y., O.B., M.G., and S.S. were involved in biological sample handling, storage, and execution of immunoassay execution. K.T. prepared the original draft of the manuscript. J.S.T.L., S.L.S., A.M.-R., and M.S.C. reviewed and edited the initial draft. All authors agreed to submit the manuscript, read and approved of the final version, and take full responsibility of its content, including the accuracy of the data and the fidelity of the trial to the registered protocol and its statistical analysis.

## Declaration of interests

Unrelated financial interests are as follows: S.A.-C. is supported by the Marg Meikle Professorship for Parkinson’s Research and has received grant funding from the Pacific Parkinson’s Research Institute, the Weston Family Foundation, Parkinson Canada, Parkinson Society British Columbia, Rick’s Heart Foundation, Canadian Institutes of Health Research (CIHR), Jack and Darlene Poole Foundation, VGH and UBC Hospital Foundation, and UBC Research Excellence Clusters program. S.A.-C. is the site principal investigator for industry-funded clinical trials by AbbVie, Praxis Precision Medicine, Biohaven, and BlueRock/Bayer. She has received honoraria for teaching by Merz and AbbVie and consulting fees from Sunovion, AbbVie, Ipsen, Knight, and Merz. K.T. is funded by the Canada Graduate Scholarships Doctoral (CGS-D) and UBC MD/PhD program. J.S.T.L. was funded by a Parkinson Canada Graduate Student Award.

## STAR★Methods

### Key resources table

**Table:** 

REAGENT or RESOURCE	SOURCE	IDENTIFIER
**Biological samples**		

Parkinson’s disease patients' blood, preserved and fresh frozen fecal samples.	Pacific Parkinson’s Research Center at the University of British Columbia	Ethics number: H21-03747

**Chemicals, peptides, and recombinant proteins**		

HCl	N/A	N/A
2-Ethylbutyric acid	N/A	N/A

**Critical commercial assays**		

CRP Developer kit Simoa™ assay	Quanterix Ltd	Item #: 100-0018
IL-12p70, IL-1β, IL-6, IL-8, TNFα, and IL-10 from CorPlex Human Cytokine 10-plex Panel 1 assay	Quanterix Ltd	Item #: 85-0329
Serum Calprotectin ELISA	Immundiagnostik Inc.	K6935
Stool Calprotectin ELISA	Immundiagnostik Inc.	K6927
Serum Zonulin ELISA	Immundiagnostik Inc.	K5601
Stool Zonulin ELISA	Immundiagnostik Inc.	K5600

**Deposited data**		

Human subject metadata, sample biomarker data and microbiome sequencing data.	Borealis Dataverse	https://borealisdata.ca/previewurl.xhtml?token=e328ee49-974d-4089-afec-4224c371f3ca

**Software and algorithms**		

fastp v0.24.0	OpenGene	https://github.com/opengene/fastp
FastQC v0.11.5	Babraham Bioinformatics	https://www.bioinformatics.babraham.ac.uk/projects/fastqc/
hostile v1.1.0	Bede Constantinides	https://github.com/bede/hostile
Kraken 2	Derrick Wood	https://github.com/DerrickWood/kraken2/wiki
Super-Focus	Genivaldo Silva	https://github.com/metageni/SUPER-FOCUS
Homo sapiens genome assembly	GCF_009914755.1	T2T-CHM13v2.0
Human major histocompatibility complex variants	IPD-IMGT/HLA	v3.51
PlusPF database	N/A	version 2024-06-05
SEED database	N/A	https://pubseed.theseed.org/FIG/seedviewer.cgi
R software (v.4.4.3)	R Foundation	https://www.r-project.org
*broom, broom.mixed, dplyr, dr4pl, emmeans, flextable, ggplot2, ggpubr, ggstatsplot, gtsummary, lme4, lmerTest, magrittr, nlme, officer, patchwork, permute, picante, purrr, readr, readxl, reshape2, tibble, tidyr, vegan* packages	R CRAN	https://cran.r-project.org
*volatility* package	Thomaz F. S. Bastiaanssen	https://github.com/thomazbastiaanssen/Volatility
*microViz* package	David Barnett	https://david-barnett.github.io/microViz/
*NetCoMi* package	Stefanie Peschel	https://github.com/stefpeschel/NetCoMi
MaAsLin3 package	bioBakery	https://github.com/biobakery/maaslin3
*DirichletMultinomial, phyloseq, microbiome*	Bioconductor	https://www.bioconductor.org/
Analysis code	Supplementary File 3	Data S2

**Other**		

OMNIgene®·GUT OM-200 kit	QIAGEN	OM-200
Bio-Tek MagBind Environmental DNA 96 Kit	Omega	N/A
Nextera library preparation kit	Illumina	N/A

### Experimental model and study participant details

The Mediterranean-Ketogenic Dietary Interventions in Parkinson’s Disease (KIM) Trial (ClinicalTrials.gov: NCT05469997) was conducted between April 2023 and October 2024 and approved by the University of British Columbia Clinical Research Ethics Board (H21-03747). Written informed consent was obtained from all participants prior to enrollment. All participants consented to the de-identified dissemination of study findings as a part of the informed consent process.

People with Parkinson’s disease (PwP) aged 40–85 years with mild-to-moderate disease severity (Hoehn and Yahr stages 1–3) receiving stable anti-parkinsonian medication regimens, or no medication, were eligible for inclusion. Participant demographic and baseline clinical characteristics are summarized in Table 2. A total of 52 participants (median age 68 [IQR: 63–74]; 31% female) took part in the study. Forty-one (84%) participants identified as Caucasian, 4 (8.2%) as East Asian, 3 (6.1%) as South Asian and 1 (2.0%) as Black/African. Participants were randomized 1:1 to intervention sequence using variable block sizes generated by an independent study team member who was not involved in participant enrollment or outcome assessment.

Sex and gender were self-reported. The study was inadequately powered to statistically evaluate the effects of sex or gender on trial outcomes.

### Method details

#### Trial design

The trial employed a randomized crossover design comparing two dietary interventions: a Mediterranean-ketogenic diet (MeDi-KD) and a Mediterranean diet supplemented with medium-chain triglyceride oil (MeDi-MCT). Full details of the study design have been reported elsewhere.^39^

The MeDi-KD consisted of a Mediterranean-style ketogenic diet providing approximately 4–10% of calories from carbohydrates, 10–20% from lean protein, and 69–82% from predominantly unsaturated fats. The MeDi-MCT intervention consisted of a standard Mediterranean diet supplemented with up to 20 mL of medium-chain triglyceride oil twice daily.

Participants attended in-person visits at the beginning and end of each intervention period. At each visit, fasting blood samples and fecal samples collected at home during the preceding week were obtained, clinical assessments were administered, and participants met with the study dietitian. Participants received intervention-specific recipes and completed weekly dietary adherence calls throughout the study.

A summary of biological samples available for each assay and study timepoint is provided in Table S1.

#### Clinical assessments

Participants completed standardized assessments evaluating motor symptoms, gastrointestinal function, cognition, neuropsychiatric symptoms, fatigue, physical activity, and quality of life (Table 1). These included the MDS-UPDRS, MoCA, PD-CRS, PD-CFRS, Beck Depression Inventory-II, Starkstein Apathy Scale, Parkinson Anxiety Scale, Fatigue Severity Scale, PDQ-39, WHO Quality of Life questionnaire, PASIPD, Bristol Stool Scale, Rome III criteria, Patient Global Impression of Change, and Diet History Questionnaire-II. Bowel movement frequency, Bristol stool scores, and laxative use were prospectively recorded in participant journals.

#### Plasma inflammatory biomarkers

Fasting plasma samples collected before and after each intervention were analyzed for CRP, IL-12p70, IL-1β, IL-6, IL-8, TNF-α, and IL-10 using Simoa assays (Quanterix). Assays were performed at the Core Facility for Neurology Biomarker Innovation (University of British Columbia) according to manufacturer protocols.

All samples from an individual participant were analyzed on the same assay plate. Participant groups were randomized across plates and samples randomized within plates. Laboratory personnel were blinded to treatment allocation. Samples were analyzed in duplicate and averaged for downstream analyses. Samples exhibiting excessive variability or assay quality concerns were re-run where possible.

#### Fecal sample processing

Participants collected both fresh-frozen and preserved stool samples before study visits. Preserved samples were collected using OMNIgene-GUT kits (OM-200; DNA Genotek Inc., Canada) and stored according to manufacturer instructions. All specimens were stored at −80 °C until batch processing. Preserved samples were available for most collections; fresh-frozen specimens were used when preserved collections were unavailable.

Fecal samples were processed according to standard laboratory procedures (fresh frozen) and manufacturer instructions (preserved) and stored at −80 °C until all samples were returned and ready for analysis in a single batch.

A total of 148 unique fecal samples (Table S1) were submitted to Microbiome Insights (Richmond, Canada) for DNA extraction, shallow shotgun metagenomic sequencing, and SCFA quantification, with technicians blinded to treatment allocation. The majority of the submitted samples were preserved samples collected using the OMNIgene-Gut kit (128/148, 86%); however, in the remaining 20 instances (14%), participants were unable to successfully collect preserved fecal samples using the kit. In these cases, fresh frozen samples were submitted for analysis as substitutes for preserved samples on the advice of company technical experts, as prior studies have demonstrated comparable taxonomic sequencing profiles using both fresh frozen and preserved collection methods.^178^ Similarities in overall taxonomic composition between fresh frozen and preserved sample types were verified using Permutational Multivariate Analysis of Variance (PERMANOVA) tests for differences in beta-diversity prior to downstream analysis of sequencing data. Given previous reports suggesting greater variability in SCFA stability between preserved and fresh frozen stool samples,^179^^,^^180^ sample type (preserved vs. fresh-frozen) was included as a covariate in all between-intervention comparisons of fecal SCFA content. For within-intervention analyses of SCFA content, only matched baseline/post-intervention sample pairs collected using the same method were included. Besides this criterion, all available data meeting assay-specific quality control criteria were retained for analysis.

#### DNA extraction and metagenomic sequencing

DNA extraction was performed using the Omega Bio-Tek MagBind Environmental DNA 96 Kit. DNA quality and concentration were assessed by gel electrophoresis and Qubit fluorometry. Libraries were prepared using Illumina Nextera chemistry and sequenced on an Illumina NovaSeq X platform using paired-end 150-bp reads.

Sequence processing included adapter removal, quality trimming, low-complexity read filtering, and host read removal. Taxonomic classification was performed using Kraken2 with the PlusPF database (release date: 2024–06-05). Functional profiling was performed using translated homology searches against the SEED database and annotated using Super-Focus. Taxa with fewer than 100 reads across all samples were excluded. Functional features were retained if present at counts greater than 100 in at least 5% of samples.

#### Short-chain fatty acid analysis

Fecal SCFA concentrations were quantified by gas chromatography using a TRACE 1310 gas chromatography system coupled to a flame ionization detector. Stool samples were homogenized, acidified, centrifuged, and spiked with 2-ethylbutyric acid as an internal standard prior to analysis. Acetate, propionate, butyrate, and related SCFAs were quantified from extracted supernatants.

#### Zonulin and calprotectin ELISAs

Fecal and serum zonulin and calprotectin concentrations were quantified using commercially available ELISA kits. Samples were allocated to 96-well plates using stratified randomization according to intervention group, visit number, and sex.

Absorbance measurements were acquired using a Tecan Infinite M200 PRO plate reader. Concentrations were interpolated from four-parameter logistic calibration curves generated in R. Duplicate measurements with coefficients of variation greater than 20% were excluded. Concentrations were calculated using averaged duplicate absorbance values and verified through visual inspection of fitted standard curves.

### Quantification and statistical analysis

All analyses were conducted in RStudio (version 2024.09.1 + 394). Statistical methods were reviewed by a University of British Columbia staff statistician. Statistical significance was defined as *p* < 0.05 or false-discovery-rate adjusted q < 0.05 unless otherwise specified.

For clinical outcomes, SCFAs, and inflammatory biomarkers, intervention effects were evaluated using linear mixed-effects models incorporating intervention sequence and period-specific baseline values as fixed effects and participant as a random effect. Within-intervention changes were assessed using Wilcoxon signed-rank tests. Analyses were performed using available-case data and maximum likelihood estimation.^181^

Spearman correlation coefficients were used to assess associations between continuous variables. Multiple-testing correction was performed using the Benjamini-Hochberg procedure within each family of related comparisons.

Baseline microbiome community types were identified using Dirichlet multinomial mixture modeling of core genera. Alpha diversity metrics, including observed richness, Chao1, Shannon, and Simpson indices, were calculated from rarefied count data. Associations between baseline variables and alpha diversity were assessed using linear regression, while intervention effects were evaluated using linear mixed-effects models.

Beta diversity was quantified using Aitchison distances derived from centered log-ratio transformed count tables. Community composition differences were assessed using PERMANOVA with 9,999 subject-constrained permutations to account for repeated sampling. Volatility analyses quantified longitudinal changes in microbiome composition and were compared using repeated-measures ANOVA.

Differential abundance analyses of microbial taxa and functional pathways were conducted using MaAsLin3. Models included intervention-by-timepoint interactions, intervention sequence, sequencing depth, and participant-level random effects. Features were normalized using total sum scaling with log transformation and filtered using minimum prevalence and abundance thresholds. False discovery rate correction was applied using the Benjamini-Hochberg method, with significance defined as q < 0.10.

Microbial association networks were constructed using SPRING as implemented in NetCoMi. Global network properties were compared using permutation testing, and differential pairwise associations were assessed using Fisher’s z-tests with local false-discovery-rate correction.

### Additional resources

Clinical trial registration: Mediterranean-Ketogenic Dietary Interventions in Parkinson’s Disease (KIM) Trial: https://clinicaltrials.gov/study/NCT05469997.

## Supplemental information

Document S1. Figures S1–S5 and Tables S1–S6

Data S1. Trial protocol

Data S2. Analysis code
