# Supplementary material for: Mediterranean-ketogenic vs. medium-chain triglyceride supplemented Mediterranean diet: Effects on the gut-brain-immune axis in Parkinson’s disease
Source: iScience. 2026 Oct 28;29(11):117981. doi: 10.1016/j.isci.2026.117981 (PMC13738181; doi:10.1016/j.isci.2026.117981)
Supplement: Supplementary file 1 [file mmc1.pdf]

iScience, Volume *29*

Supplemental information

Mediterranean-ketogenic vs. medium-chain triglyceride supplemented Mediterranean diet: Effects on the gut-brain-immune axis in Parkinson's disease

Kira Tosefsky, Samantha L. Schaffner, Joyce S.T. Lam, Yolanda N. Wang, Shayan Keymanesh, Annie J. Kuan, Avril Metcalfe-Roach, Mihai S. Cirstea, Matthew A. Sacheli, Tessa F. Morelli, Matthew Yap, Olivia Brigden, Madison Gill, Tamara R. Cohen, Sophie Stukas, B. Brett Finlay, and Silke Appel-Cresswell

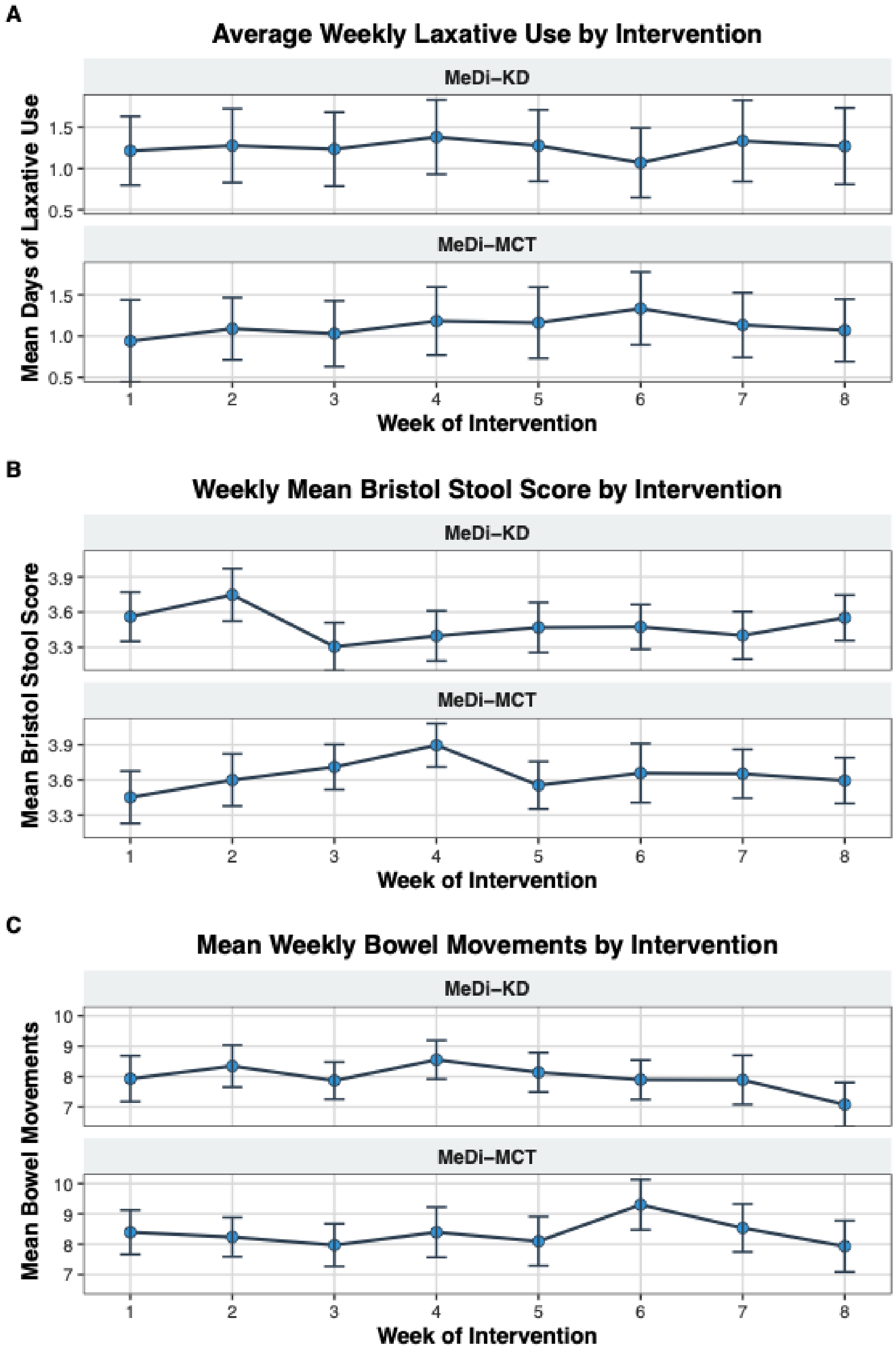

Figure S1. Changes in weekly laxative use, Bristol stool score and weekly bowel movements during the MeDi-KD vs. MeDi-MCT interventions. (A) Average number of days of laxative use, (B) average Bristol stool score per week and (C) average number of bowel movements per week during each intervention. Error bars represent standard errors of the mean (SEM).

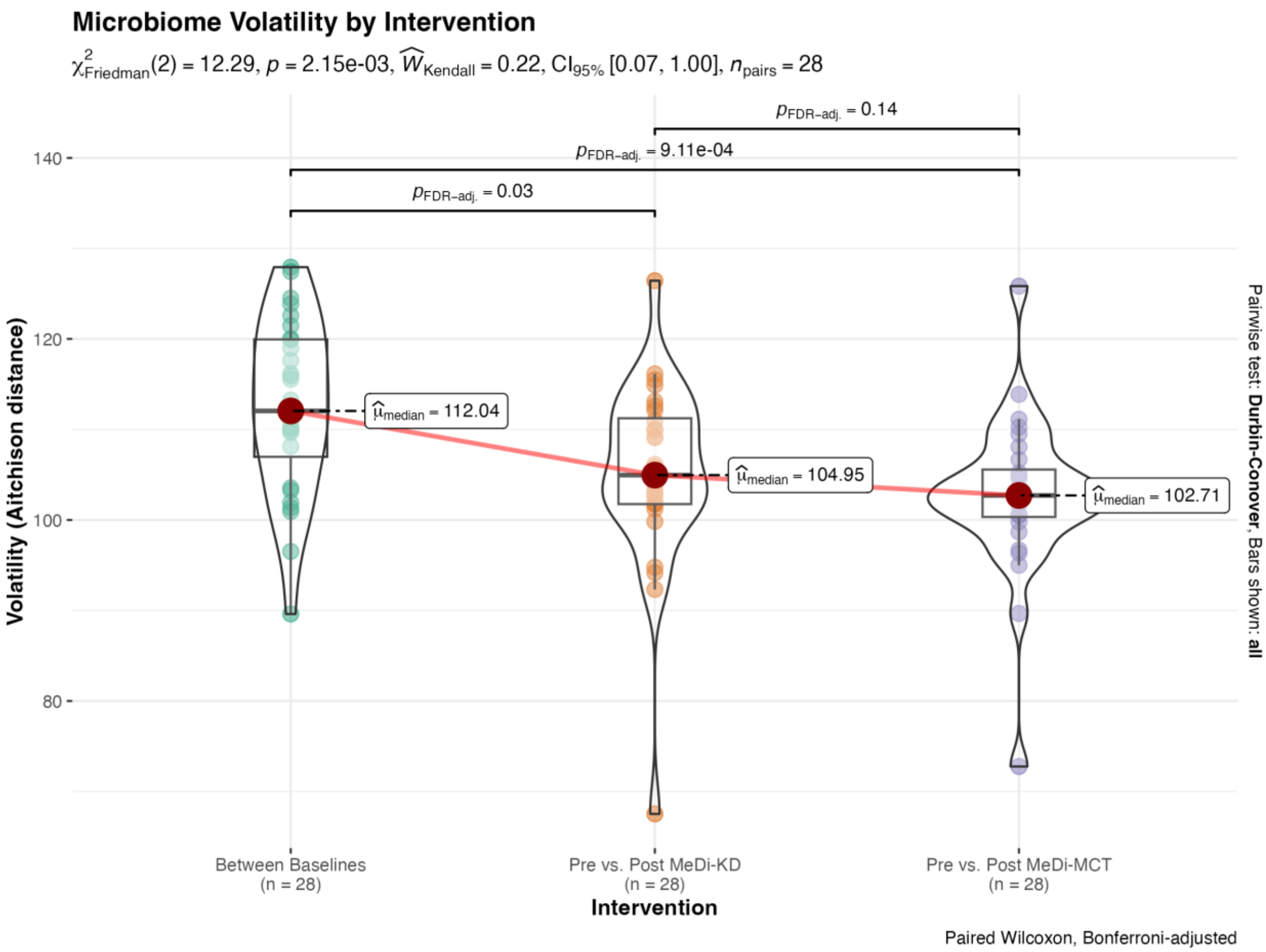

Figure S2. Microbiome volatility was significantly higher between baselines than between baseline and post-intervention follow-ups for either intervention.

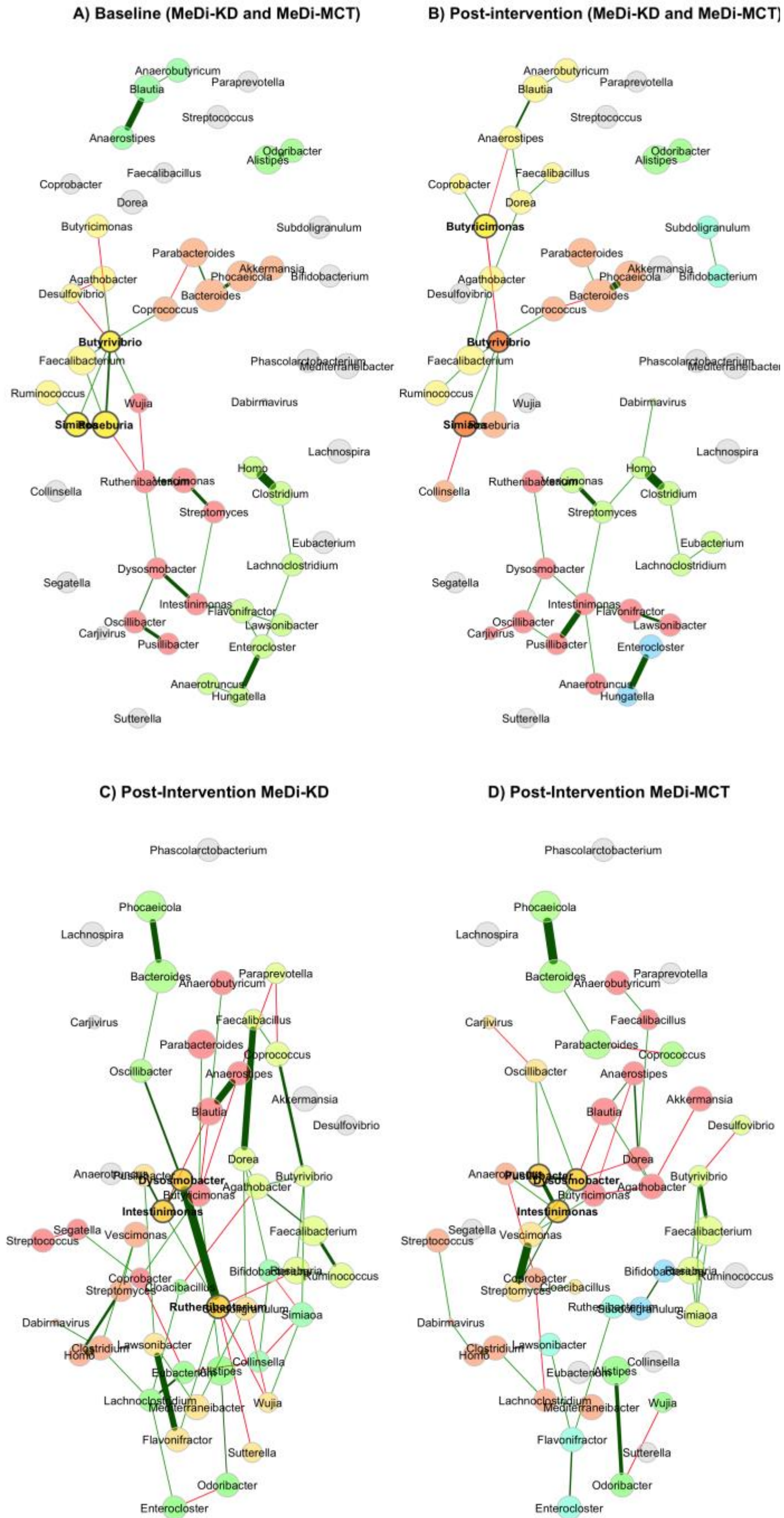

Figure S3. Microbial associations did not change significantly between timepoints (A-B) nor between interventions (C-D) in differential network analysis. (A) Baseline microbial network representation for the whole cohort. (B) Post-intervention microbial network representation for the full cohort. (C) Microbial network representation following the MeDi-KD intervention (D) Microbial network representation following the MeDi-MCT intervention. In each panel figure, each node represents an individual genus. Node colours represent communities or clusters detected during analysis. Node sizes represent abundance on the modified centered log-ratio-transformed scale. Green edges represent positive associations, and red edges represent negative associations between genera. Edge thickness corresponds to the strength of associations.

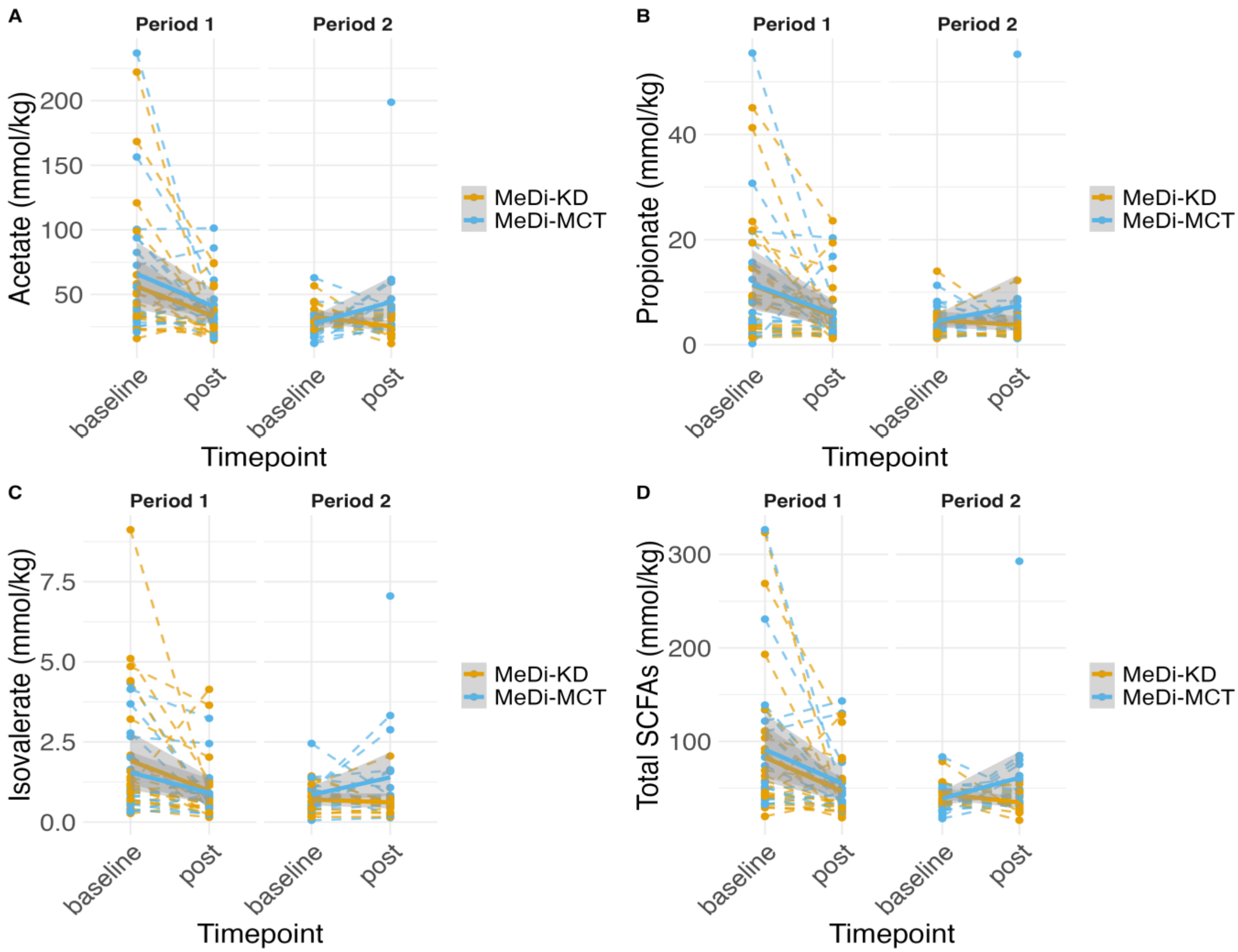

Figure S4. Intervention-related differences in changes from baseline in fecal isovaleric, acetic, propionic and total SCFA levels, separated by study period. Each of these SCFAs demonstrated statistically significant ($p<0.05$) changes from baseline in response to the MeDi-KD, but not the MeDi-MCT.

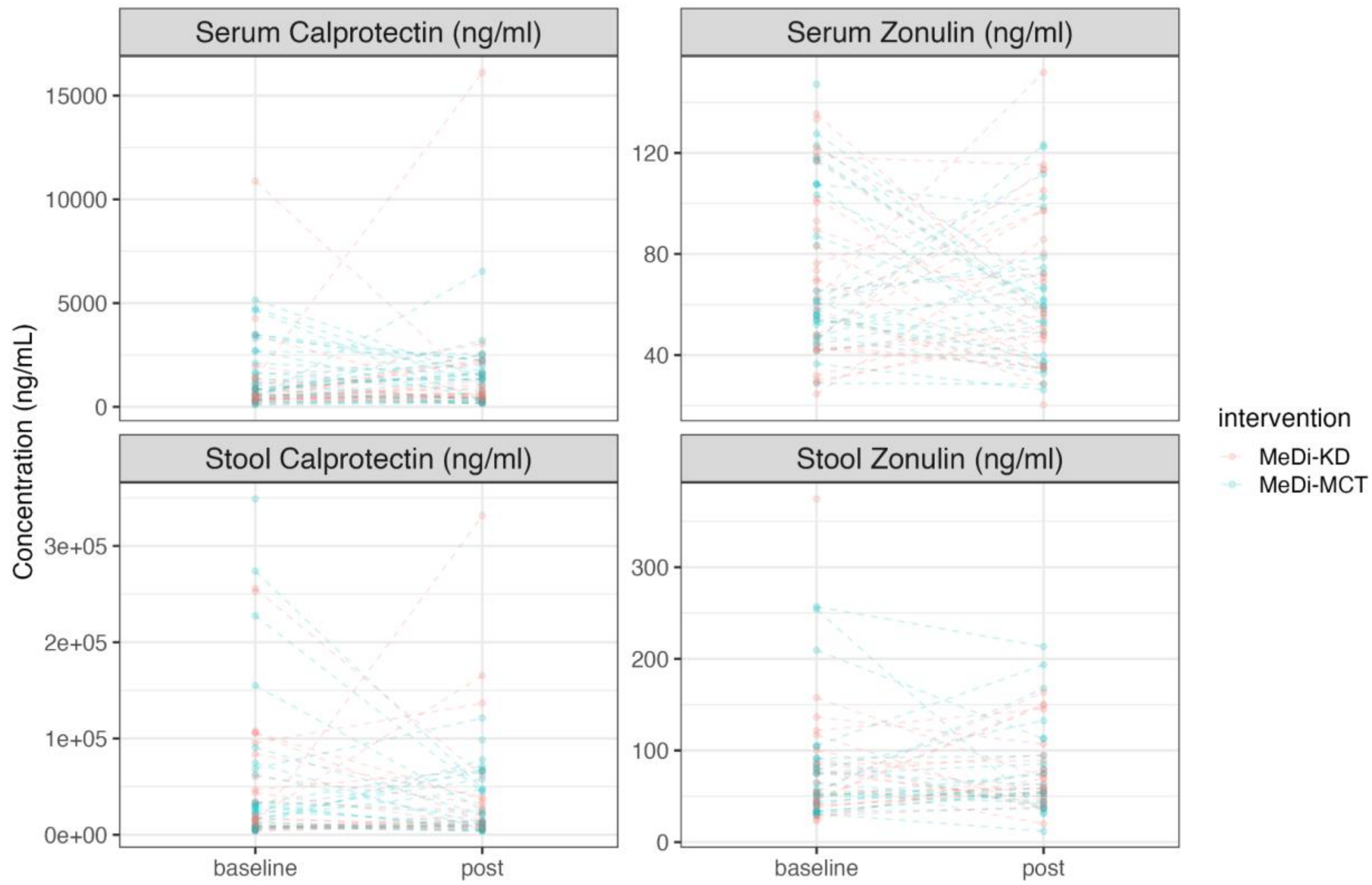

Figure S5. No significant intervention-related changes were observed in serum or stool calprotectin or zonulin concentrations.

Table S1. Summary of number of samples analyzed at each timepoint.

Sample Type	MeDi-KD Baseline	MeDi-KD Post	MeDi-MCT Baseline	MeDi-MCT Post	Second Baseline*	Total
ELISAs (Fecal)	30	25	33	27	5	120
ELISAs (Serum)	23	22	24	20	0	89
Microbiome (Fecal)	39	35	38	35	1	148
Inflammatory Biomarkers (Plasma)	42	37	46	38	1	164

*Participant attended third study visit, but did not start second intervention.

Table S2. Changes in self-reported symptom domains measured by the PGIC.

Characteristic	Overall N = 80[1]	MeDi-KD N = 39[1]	MeDi-MCT N = 41[1]	p-value[2]
Anxiety				0.8
Improvement	19 (27%)	10 (29%)	9 (24%)	
Worsening	10 (14%)	4 (12%)	6 (16%)	
No Change	42 (59%)	20 (59%)	22 (59%)	
Mood				>0.9
Improvement	26 (37%)	13 (38%)	13 (35%)	
Worsening	10 (14%)	5 (15%)	5 (14%)	
No Change	35 (49%)	16 (47%)	19 (51%)	
Wellbeing				0.8
Improvement	27 (38%)	14 (41%)	13 (35%)	
Worsening	9 (13%)	5 (15%)	4 (11%)	
No Change	35 (49%)	15 (44%)	20 (54%)	
Bowel				0.7
Improvement	35 (49%)	15 (44%)	20 (54%)	
Worsening	16 (23%)	8 (24%)	8 (22%)	
No Change	20 (28%)	11 (32%)	9 (24%)	
Motor				0.3
Improvement	20 (28%)	7 (21%)	13 (35%)	
Worsening	21 (30%)	10 (29%)	11 (30%)	
No Change	30 (42%)	17 (50%)	13 (35%)	
Fatigue				0.2
Improvement	19 (27%)	10 (29%)	9 (24%)	
Worsening	22 (31%)	13 (38%)	9 (24%)	
No Change	30 (42%)	11 (32%)	19 (51%)	

[1]n (%) Five participants in MeDi-KD and 4 in MeDi-MCT did not complete the PGIC

[2]Fisher's exact test; Pearson's Chi-squared test

Table S3. Mean (SD) levels of each assayed peripheral inflammatory biomarker at each of the four timepoints.

	MeDi-KD Baseline (pg/mL)	MeDi-KD Post (pg/mL)	MeDi-MCT Baseline (pg/mL)	MeDi-MCT Post (pg/mL)
CRP	6.52×10^6 (1.34×10^7)	4.45×10^6 (5.79×10^6)	6.25×10^6 (9.80×10^6)	1.00×10^7 (3.87×10^7)
IL-10	0.60 (0.76)	0.63 (0.75)	0.56 (0.67)	0.66 (1.15)
IL-12p70	0.32 (0.99)	0.16 (0.10)	0.19 (0.12)	0.15 (0.09)
IL-1β	0.12 (0.06)	0.12 (0.08)	0.15 (0.10)	0.12 (0.10)
IL-6	0.91 (1.18)	0.73 (0.48)	1.27 (1.89)	1.35 (2.49)
IL-8	5.92 (5.61)	5.46 (4.89)	6.17 (6.00)	5.26 (6.98)
TNF-a	0.68 (0.58)	0.63 (0.46)	0.67 (0.44)	0.64 (0.53)

Table S4. Within-intervention changes in circulating, fecal and clinical biomarkers.

Outcome	Intervention	Wilcoxon V	p-value	Effect size r
Acetate (mmol/kg)	MeDi-KD	104.0	0.041	-0.393
Acetate (mmol/kg)	MeDi-MCT	269.0	0.133	0.284
Propionate (mmol/kg)	MeDi-KD	94.0	0.022	-0.439
Propionate (mmol/kg)	MeDi-MCT	195.0	0.855	-0.034
Isobutyrate (mmol/kg)	MeDi-KD	110.0	0.058	-0.365
Isobutyrate (mmol/kg)	MeDi-MCT	238.0	0.425	0.151
Butyrate (mmol/kg)	MeDi-KD	129.0	0.149	-0.277
Butyrate (mmol/kg)	MeDi-MCT	237.0	0.439	0.146
Isovalerate (mmol/kg)	MeDi-KD	84.0	0.012	-0.485
Isovalerate (mmol/kg)	MeDi-MCT	221.0	0.682	0.077
Valerate (mmol/kg)	MeDi-KD	109.0	0.055	-0.370
Valerate (mmol/kg)	MeDi-MCT	249.0	0.295	0.198
Hexanoate (mmol/kg)	MeDi-KD	169.0	0.631	-0.092
Hexanoate (mmol/kg)	MeDi-MCT	280.0	0.080	0.331
Isocaproate (mmol/kg)	MeDi-KD	178.0	0.792	0.051
Isocaproate (mmol/kg)	MeDi-MCT	188.0	0.733	0.065
Heptanoate (mmol/kg)	MeDi-KD	199.0	0.810	-0.046
Heptanoate (mmol/kg)	MeDi-MCT	184.0	0.665	-0.082
Total SCFAs (mmol/kg)	MeDi-KD	103.0	0.039	-0.398
Total SCFAs (mmol/kg)	MeDi-MCT	258.0	0.210	0.237
MDS-UPDRS Part I item 1.11	MeDi-KD	16.0	0.763	0.000
MDS-UPDRS Part I item 1.11	MeDi-MCT	32.5	0.963	0.000
Serum calprotectin (ng/mL)	MeDi-KD	40.0	0.534	0.188
Serum calprotectin (ng/mL)	MeDi-MCT	46.0	0.583	0.159
Serum zonulin (ng/mL)	MeDi-KD	59.0	0.642	0.116
Serum zonulin (ng/mL)	MeDi-MCT	51.0	0.133	-0.354
Stool calprotectin (ng/mL)	MeDi-KD	104.0	0.689	-0.087

Outcome	Intervention	Wilcoxon V	p-value	Effect size r
Stool calprotectin (ng/mL)	MeDi-MCT	97.0	0.765	-0.067
Stool zonulin (ng/mL)	MeDi-KD	121.0	0.550	0.134
Stool zonulin (ng/mL)	MeDi-MCT	109.0	0.573	0.129
CRP (pg/mL)	MeDi-KD	163.0	0.989	0.003
CRP (pg/mL)	MeDi-MCT	113.0	0.040	-0.387
IL-12p70 (pg/mL)	MeDi-KD	174.0	0.757	0.062
IL-12p70 (pg/mL)	MeDi-MCT	188.0	0.733	-0.065
IL-1β (pg/mL)	MeDi-KD	137.0	0.493	-0.137
IL-1β (pg/mL)	MeDi-MCT	119.0	0.056	-0.361
IL-6 (pg/mL)	MeDi-KD	186.0	0.527	0.126
IL-6 (pg/mL)	MeDi-MCT	148.0	0.210	-0.237
IL-8 (pg/mL)	MeDi-KD	109.0	0.150	-0.288
IL-8 (pg/mL)	MeDi-MCT	55.0	0.001	-0.637
TNF-α (pg/mL)	MeDi-KD	157.0	0.882	0.030
TNF-α (pg/mL)	MeDi-MCT	125.0	0.076	-0.336
IL-10 (pg/mL)	MeDi-KD	192.0	0.427	0.159
IL-10 (pg/mL)	MeDi-MCT	176.0	0.539	-0.116
Total cholesterol (mmol/L)	MeDi-KD	69.0	0.609	0.132
Total cholesterol (mmol/L)	MeDi-MCT	86.0	0.478	-0.159
Triglycerides (mmol/L)	MeDi-KD	40.0	0.148	-0.362
Triglycerides (mmol/L)	MeDi-MCT	116.0	0.184	0.297
HDL cholesterol (mmol/L)	MeDi-KD	48.5	0.802	0.063
HDL cholesterol (mmol/L)	MeDi-MCT	48.5	0.105	-0.362
LDL cholesterol (mmol/L)	MeDi-KD	77.0	0.334	0.241
LDL cholesterol (mmol/L)	MeDi-MCT	102.5	0.763	0.067

Table S5. Changes in alpha diversity metrics in response to each intervention.

Alpha diversity metric	Intervention	p-value	Effect size r
Observed ASVs	MeDi-KD	0.206	-0.217
Observed ASVs	MeDi-MCT	0.681	0.073
Shannon diversity	MeDi-KD	0.135	-0.257
Shannon diversity	MeDi-MCT	0.466	0.129
Simpson diversity	MeDi-KD	0.139	-0.254
Simpson diversity	MeDi-MCT	0.379	0.155
Chao1 richness	MeDi-KD	0.489	-0.119
Chao1 richness	MeDi-MCT	0.722	0.063

Table S6. Associations between blood-based biomarkers and microbiome diversity metrics.

A. IL-6 (pg/mL) associations with α-diversity metrics

Characteristic	N	β	95% CI	p-value
Observed	42	-6.7e-05	-1.2e-04, -1.3e-05	0.016
Chao1	42	-7.3e-05	-1.4e-04, -1.1e-05	0.022
Shannon	42	-1.5e-01	-2.9e-01, -1.2e-02	0.034
Simpson	42	-9.4e-01	-2.4e+00, 5.4e-01	0.206

Abbreviation: CI = Confidence Interval

B. Total Cholesterol (mmol/L) associations with α-diversity metrics

Characteristic	N	β	95% CI	p-value
Observed	39	-2.1e-04	-4.4e-04, 2.0e-05	0.072
Chao1	39	-2.6e-04	-5.2e-04, -2.1e-06	0.048
Shannon	39	-6.7e-01	-1.2e+00, -9.8e-02	0.023
Simpson	39	-6.4e+00	-1.2e+01, -7.0e-01	0.029

Abbreviation: CI = Confidence Interval

C. LDL cholesterol (mmol/L) associations with α-diversity metrics

Characteristic	N	β	95% CI	p-value
Observed	39	-1.9e-04	-3.9e-04, 1.4e-05	0.067
Chao1	39	-2.6e-04	-4.8e-04, -3.0e-05	0.027
Shannon	39	-6.5e-01	-1.2e+00, -1.5e-01	0.012
Simpson	39	-6.5e+00	-1.1e+01, -1.6e+00	0.011

Abbreviation: CI = Confidence Interval

D. Stool zonulin (ng/ml) associations with α-diversity metrics

Characteristic	N	β	95% CI	p-value
Observed	24	-7.0e-03	-1.3e-02, -5.6e-04	0.035
Chao1	24	-8.7e-03	-1.6e-02, -1.5e-03	0.020
Shannon	24	-1.4e+01	-3.1e+01, 2.9e+00	0.098

Characteristic	N	β	95% CI	p-value
Simpson	24	-7.6e+01	-2.4e+02, 8.9e+01	0.351

Abbreviation: CI = Confidence Interval

E. Saturated fatty acids (% energy) associations with α-diversity metrics

Characteristic	N	β	95% CI	p-value
Observed	35	-3.1e-04	-7.8e-04, 1.6e-04	0.190
Chao1	35	-3.1e-04	-8.5e-04, 2.4e-04	0.258
Shannon	35	-1.2e+00	-2.3e+00, -1.2e-01	0.031
Simpson	35	-1.5e+01	-2.6e+01, -4.9e+00	0.006

Abbreviation: CI = Confidence Interval
