# Supplementary material for: Mediterranean-ketogenic vs. medium-chain triglyceride supplemented Mediterranean diet: Effects on the gut-brain-immune axis in Parkinson’s disease
Source: iScience. 2026 Oct 28;29(11):117981. doi: 10.1016/j.isci.2026.117981 (PMC13738181; doi:10.1016/j.isci.2026.117981)
Supplement: Supplementary file 2 [file mmc2.pdf]

Protocol Cover Page

STUDY TITLE

Ketogenic Diet Interventions in Parkinson's Disease: Safeguarding the Gut Microbiome

SHORT TITLE

KIM

PROTOCOL NAME

KIM-05

PROTOCOL IDENTIFYING NUMBER

KIM-04

PROTOCOL VERSION DATE

V 4.0 – May 18, 2023

Approved by PI: **August 10, 2022**

Name: Silke Appel-Cresswell **Signature:**

Table of Content

Protocol Cover Page	i
List of Abbreviations	1
Study Summary	2
Study Visit Summary	3
**1. Introduction**	5
1.1. Background and Rationale	5
**2. Objectives**	6
**3. Hypotheses**	6
**4. Study design**	7
4.1. Inclusion Criteria	7
4.2. Exclusion Criteria	7
4.3. Study Procedure	7
4.3.1 Participant Recruitment	7
4.3.2 Randomization	8
4.3.3 Participant Consent, Screening, and Initial Study Visit	8
4.3.4 fecal Sample	8
4.3.5 Blood Sample	9
4.3.6 Study Intervention	9
4.3.7 Participant Monitoring	10
4.3.8 Subsequent Study Visit	10
4.3.9 Assessment of Compliance	11
4.3.9.1 Dietary compliance	11
4.3.9.2 MCT-supplement Adherence	12
4.3.10 Premature Withdrawal/Discontinuation Criteria	12
4.3.11 Rescue Medication	12
4.3.12 Washout Period	12
4.3.13 Concomitant Medication	13
4.4. Study Monitoring	13
**5. Analysis**	13
5.1 Biological sampling and Analysis	13
5.1.1 Blood Sampling	13
5.1.2 Fecal Sampling	13
5.1.3 Fecal Sample Analysis	13
5.1.4 Blood Sample Analysis	14
5.2. Demographic and Clinical Information	15
5.3 Statistical Analysis	15
**6. Adverse Event Reporting**	15
6.1 Adverse Event Definitions	16
6.1.1 Adverse Event	16
6.1.2 Serious Adverse Event	16
6.1.3 Unlisted (Unexpected) Adverse Events	17
6.1.4 Life-threatening	17

6.1.5 Associated with the use of the Drug	17
**7. Trial Conduct**	17
8. Maintenance/Database	17
8.1. Confidentiality and Data Storage	17
8.2. Source Data/Documentation	18
8.3. Ethical Consideration	18
**9. Risks**	19
9.1 MCT Supplementation	19
9.2 The Mediterranean-Ketogenic Diet	19
9.3 Venipuncture	19
9.4 Non-physical risks	19
**10. Benefits**	21
**11. Reimbursement**	21
**12. Significance**	21
**13. References**	22
**14. Appendices**	30
14.1 Montreal Cognitive Assessment (MoCA)	30
14.2 Movement Disorder Society – Unified Parkinson's Disease Rating Scale (MDS-UPDRS)	31
14.3 Beck Depression Inventory, 2nd Edition (BDI-II)	32
14.4 Fatigue Severity Scale (FSS)	33
14.5 Parkinson's Anxiety Scale (PAS)	34
14.6 Starkstein Apathy Scale (AS)	35
14.7 Parkinson's Disease Questionnaire – 39 (PDQ-39)	36
14.8 ROME III Constipation Module (ROME III)	38
14.9 The Physical Activity Scale for Individuals with Physical Disabilities (PASIPD)	40
14.10 Diet History Questionnaire II	42
14.11 Bristol Stool Chart	52
14.12 MIND Diet Score	53
14.13 Mediterranean Diet Adherence Screener	54
14.14 Nutrition Information Form	55
14.15 Parkinson's Disease Cognitive Rating Scale (PD-CRS)	
14.16 Parkinson's Disease Cognitive Functional Rating Scale (PD-CFRS)	

List of Abbreviations

AE	Adverse Events
ANCOVA	Analysis of covariance
AS	Starkstein Apathy Scale
BDI-II	Beck Depression Inventory – II
BHB	Beta-Hydroxybutyrate
C-DHQ	Canadian version of Dietary History Questionnaire – II
CRA	Clinical Research Associate
CRF	Case Report Forms
CRP	C-Reactive Protein
DMCBH	Djavad Mowafaghian Centre for Brain Health
ELISA	Enzyme-linked immunosorbent assay
FFS	Fatigue Severity Scale
GC-MS	Gas Chromatography Mass Spectroscopy
GCP	Good Clinical Practice
hsCRP	High sensitivity C-Reactive protein
IBD	Inflammatory Bowl Disease
IBS	Irritable Bowl Syndrome
KB	Ketone Bodies
KD	Ketogenic Diets
LPS	Lipopolysaccharide
MCT	Medium-Chain Triglycerides
MDS	Movement Disorder Society
MDS-UPDRS	Movement Disorder Society – Unified Parkinson's Disease Rating Scale
MeDi	Mediterranean Diet
MeDi-KD	Mediterranean-Ketogenic Diet
MeDi-MCT	Mediterranean diet with MCT oil supplementation
MoCA	Montreal Cognitive Assessment
OTC	Over the Counter
PAS	Parkinson Anxiety Scale
PASIPD	The Physical Activity scale for Individuals with Physical Disabilities
PD	Parkinson's Disease
PD-CRS	Parkinson's Disease Cognitive Rating Scale
PD-CFRS	Parkinson's Disease Cognitive Functional Rating Scales
PDQ-39	Parkinson's Disease Questionnaire – 39
PI	Principal Investigator
PPRC	Pacific Parkinson Research Centre
SAE	Serious Adverse Event
UAE	Unlisted/unexpected adverse event
UPDRS	Unified Parkinson's Disease Rating Scale

WOCBP	Women of Childbearing Potential

Study Summary

Title	**Ketogenic Diet Interventions in Parkinson's Disease: Safeguarding the Gut Microbiome**
Short Title	KIM
Protocol Number	KIM-04
Phase	Phase IV
Design	A 24-week random order, crossover design clinical trial of two 8-week interventions – Mediterranean Ketogenic Diet (MeDi-KD) and Mediterranean Diet with MCT Oil Supplementation (MeDi-MCT) – separated by 8 weeks of washout
Study Duration	24 weeks per subject/ 24 months for completion of study
Study Centres	Djavad Mowafaghian Centre for Brain Health, University of British Columbia, BC
Objectives	To investigate two Mediterranean-Ketogenic dietary interventions with a focus on gut health safety in patients with Parkinson's Disease
Number of Subjects	50
Diagnosis and Main Inclusion Criteria	• Age between 40-85 years<br>• PD diagnosis based on MDS criteria [52]<br>• Hoehn & Yahr score of 1 to 3<br>• On stable dopaminergic medication for at least one month
Study Intervention, Dose, Route, Regimen	For the MeDi-MCT Arm: Adherence to a Mediterranean diet while supplementing with Medium Chain Triglyceride oil of varying doses (up to 40 mL of MCT per day) taken orally twice daily.<br>For the MeDi-KD Arm: Adhering to a Mediterranean-Ketogenic Diet. Requires limiting carbohydrate intake to about 10% of daily caloric intake while obtaining the remaining calories from MeDi-friendly fats (~70% of total daily energy) and proteins (~20% of total daily energy)
Duration of Administration	8-weeks for each intervention arm and washout for each subject to complete per protocol

	Screening	Pre-intervention 1 (visit 1)	Post-intervention 1 (visit 2)	Pre-intervention 2 (visit 3)	Post-intervention 2 (visit 4)
**Informed consent**	X				
**Participant Health Assessment**					
Demographic Information (sex, age, year of diagnosis, age of onset, initial symptoms)	X				
Medication overview	X	X	X	X	X
Past Medical History	X				
Weight	X	X	X	X	X
Height	X (verbal)	X (Measured)			
Waist Circumference	X	X	X	X	X
Pregnancy Test (WOCBP)	X				
**Other Participant Information**					
Family History/Ancestry		X			
Alcohol Use/Smoking		X			
Occupation		X			
Gynecological History		X			
Education and Socioeconomic Status		X			
**Clinical Assessments**					
Montreal Cognitive Assessment (MoCA)	X*			X	
Movement Disorder Society - Unified Parkinson's Disease Rating Scale (MDS-UPDRS)	X*		X	X	X
**Questionnaires**					
Fatigue Severity Scale (FSS)		X	X	X	X
Parkinson Anxiety Scale (PAS)		X	X	X	X
Starkstein Apathy Scale (AS)		X	X	X	X
Beck Depression Inventory, 2nd Ed. (BDI-II)	X*		X	X	X
Parkinson's Disease Questionnaire – 39 (PDQ-39)		X	X	X	X
ROME III Constipation Module (ROME III)		X	X	X	X
The Physical Activity Scale for Individuals with Physical Disabilities (PASIPD)		X	X	X	X
Diet History Questionnaire II (DHQ-II)		X			
Nutrition Information Form		X			
MIND Diet Score		X	X	X	X

Mediterranean Diet Adherence Screener (MEDAS)	X	X	X	X
Bristol Stool Chart	X	X	X	X
Parkinson's Disease Cognitive Rating Scale ***(PD-CRS)***	X	X	X	X
Parkinson's Disease Cognitive Functional Rating Scale ***(PD-CFRS)***	X	X	X	X
Patient Global impression of Change (PGIC)		X		X
WHO Quality of Life Questionnaire	X	X	X	X
**Sample Collection**				
Blood Sample	X	X	X	X
Fecal Sample	X	X	X	X
**Qualitative Interview**		X		X

***** These interventions will be done in person during visit one and will serve as both a screen tool and baseline measurements.

1. Introduction

1.1. Background and Rationale

Parkinson's disease (PD), the second most common and the most rapidly growing neurodegenerative disease worldwide [1,2], remains without a cure or neuroprotective therapy other than potentially exercise [3]. Gut-related symptoms are common and often the initial symptoms, suggesting a possible intestinal origin of PD [4]. Transplantation of fecal matter from human donors with PD compared to healthy controls leads to worsening of PD features and inflammation in an alpha-synuclein overexpressing mouse model [5]. Over a dozen studies have demonstrated gut dysbiosis in PD with reduced diversity, increased pro-inflammatory capacity, and decreased Short-Chain Fatty Acids (SCFA) production as key characteristics [5-10] and persistently increased relative abundance of Akkermansia [5-17]. Integration of these findings [7] proposes that a lack of dietary fiber intake in PD leads to reduced production of SCFA including butyrate, which provides energy for epithelial cells and in turn a reduced mucin layer. Simultaneously, increased Akkermansia levels lead to decomposition of the mucin layer. Collectively, these alterations may result in compromised intestinal barrier function, gut inflammation, invasion of pathogens, presumed exposure of the intestinal neural plexus to lipopolysaccharides and environmental toxins and aggregation of alpha-synuclein fibrils [7].

Emerging evidence suggests that both ketogenic [18-23] and Mediterranean diets [24-30] have beneficial and likely complementary effects in PD. Combining principles from both diets would likely optimize dietary benefits. To the best of our knowledge, no clinical trials have been performed into combined ketogenic and Medi-stye dietary interventions in PD yet.

Mediterranean diets (MeDi) are primarily but not exclusively plant-based [24]. Their promotion of high fiber content intake promotes the production of SCFA and are associated with improved gut microbiome diversity, reduced oxidative stress, and improved insulin sensitivity [25]. Adherence to MeDi in PD is associated with a reduced risk to develop parkinsonism or prodromal PD [26-29] and higher age of onset of PD [30].

Ketogenic diets (KD) are high in fat, adequate in protein and very low in carbohydrates [31]. Unlike the MeDi, KD can provide ketone bodies (KB) [32] as an alternative fuel source to glucose, the utilization of which is perturbed in the PD brain [33]. Fatty acids are converted to KBs such as beta-hydroxybutyrate (BHB) [31-35]. BHB, in turn, is used for mitochondrial ATP generation [32] through bypassing the deficient complex I activity in the PD mitochondria [33]. Additionally, BHB also exhibits anti-oxidative [36] and anti-inflammatory properties [37] (review by [38]).

KDs have been successfully used for decades in epilepsy [39] and more recently in diabetes [40], obesity [41], and neurological disorders [18-19, 42]. Pilot trials in PD report improved Unified Parkinson's Disease Rating Scale (UPDRS) scores (N= 5) [20], cognitive performance (N=14) [21] and non-motor symptoms (N=47) [22] (recent review by [23]).

Another method for inducing the state of ketosis is by consumption of ketogenic medium-chain triglycerides (MCTs; 8-carbon tricaprylin, 10-carbon tricaprin) [43]. MCTs are converted to KBs, which can readily cross the blood-brain barrier and the mitochondrial membrane [43]. In other words, unlike the KDs, MCTs can elevate the blood KB levels without the need for carbohydrate intake restrictions or calory deficiency [43]. Despite a milder ketosis effect than the KD, the ketogenic benefits of MCTs appear to be largely preserved as shown in epilepsy [44] and cognitive impairment with an increase of

230% in brain ketone metabolism in mild cognitive impairment and significant cognitive improvement [45-46].

Several studies of classical KDs in non-PD populations have observed significant alterations on the gut microbiome, including an increase in Akkermansia [47]. For instance, a human study in cognitive impairment reported a 4-fold increase of Akkermansia with the Modified Mediterranean-Ketogenic diet (MeDi-KD) [48]. Akkermansia plays a critical role in the anti-seizure effect of the KD, which blooms by more than 30% within days of starting the diet [49]. The classic KD can also reduce fecal SCFA levels, which are important in promoting gut health [50].

By combining the principles of MeDi with ketogenic interventions, we hope to leverage the gut-health promoting aspects of the former with bioenergetics benefits of the later, in a safe manner.

2. Objectives

This is a phase IV proof-of concept, random order, cross-over study in participants with PD examining two 8-week interventions: (1) the Mediterranean ketogenic diet (MeDi-KD) and (2) the Mediterranean diet supplemented with medium-chain triglycerides (MeDi-MCT), separated by an 8-week washout period.

Aims:

1. To examine safety of MeDi-KD and MeDi-MCT interventions with a focus on gut inflammation, microbial composition, SCFA production, and gut-barrier integrity.

We will assess changes in short chain fatty acid (SCFA)/ butyrate production and gut microbiome compositions, levels of fecal and serum calprotectin (inflammation), and zonulin (barrier function) comparing before and after each 8-week intervention. We will track any potential adverse events.

2. To examine feasibility and adherence to the two diets.

We will assess retention and adherence rates and perform qualitative participant interviews on feasibility and barriers to following either diet, measure adherence with repeated one-day food diaries and weekly monitoring of blood beta-hydroxybutyrate levels using finger-pricking tests.

3. To explore clinical outcomes and systemic inflammation.

We will measure PD motor and non-motor including gastrointestinal symptoms as well as systemic inflammation. We will compare outcomes between the two different interventions and explore correlations between the different diet interventions, ketone levels, wet biomarkers, adherence and clinical measures.

3. Hypothesis

(H1a) Neither the MeDi-MCT nor the MeDi-KD (pre-post comparison) will be associated with significantly increased faecal calprotectin levels compared to the respective baseline.

(H1b) Neither the MeDi-MCT nor the MeDi-KD (pre-post comparison) will be associated with significantly increased relative Akkermansia levels, reduced total SCFA levels, decreased butyrate levels or reduced gut barrier function (fecal zonulin), respectively.

(H2) Retention rates for both diets will be at least 75%.

4. Study Design

This safety study consists of two 8-week interventions (MeDi-MCT and MeDi-KD) in random order using a cross-over design, separated by an 8-week washout period.

The focus of this study is on safety and changes in gut microbiome function and inflammation, outcomes not expected to be significantly driven by placebo effects, thus neither intervention will be placebo-controlled in favor of a larger sample size and higher analytic power. The 8-week intervention period is based on previous ketogenic interventions that have observed significant changes in the microbiome in trials of 8 weeks duration or less [48,50,51].

4.1. Inclusion Criteria

- Age between 40-85 years
- PD diagnosis based on Movement Disorder Society (MDS) criteria [52]
- Hoehn & Yahr score of 1 to 3
- On stable dopaminergic medication for at least one month

4.2. Exclusion Criteria

- Atypical parkinsonism
- Medical or psychiatric conditions that would prevent full participation in the nutrition intervention
- Pregnancy
- Significant dysphagia
- Diabetes on insulin
- Anti-coagulation on warfarin
- Inflammatory bowel disease
- Dementia defined by Montreal Cognitive Assessment (MoCA) Scores of less than 21
- Inability to fill in electronic questionnaires or understand study instructions
- Use of immunomodulatory agents
- Probiotic use in the last 4 weeks (except for dietary sources such as yoghurt, kefir etc.), or antibiotic use in the last 3 months prior to the trial
- Use of MCT oil or on ketogenic diet in last 8 weeks prior to the trial
- Allergic to MCT oil, coconut oil, or coconut

4.3. Study Procedure

4.3.1. Participant Recruitment

Participants will include patients with a confirmed diagnosis of PD from the tertiary UBC Movement Disorder Clinic, the REACH BC platform (https://www.reachbc.ca/), BC Brain Wellness Program (www.bcbrainwellness.ca, see appendix), communications of the Djavad Mowafaghian Centre for Brain Health, the Vancouver Coastal Health Research Institute, and social media communications. If individuals from other clinics/centers are interested in participating in this clinical trial, they may reach out to a study team member at the Pacific Parkinson's Research Centre (PPRC) for more information. A diagnosis of Parkinson's disease must be confirmed by the individual's most recent consult letter from their neurologist.

Patient participants who express interest in research will meet with a study team member at the PPRC not involved in their clinical care to discuss the study, what participation entails, eligibility criteria, and the risks and benefits. Potential participants will then be given the opportunity to ask any questions they have and will be given time to decide whether participation is suitable.

The PPRC sees over 2,500 patients with Parkinson's disease per year, most of which actively participate in research. Given the recruitment rate of prior microbiome studies in PD at our Centre and the active role that PPRC patients take in research, we are confident that our recruitment schedule is feasible.

We aim to recruit 50 participants, with the aim of having at least 40 participants complete the intervention. We have estimated a 20% dropout rate.

4.3.2. Randomization

Participants will be randomly allocated to start with either the Mediterranean ketogenic diet (MeDi-KD) or the Mediterranean diet with MCT oil supplement (MeDi-MCT) with a 1:1 ratio, using variable block sizes. Randomization will be computer-generated (e.g., using the Blockrand package in R) and conducted by an independent statistician prior to the enrollment of the first participant.

4.3.3. Participant Consent, Screening, and Initial Study Visit

Willing and eligible participants will meet with the study team and review the study consent form. The consent review discussion will take place remotely or in-person at the UBC Movement Disorder Clinic. Following informed consent, individuals will complete screening assessments, including the MoCA, the Beck Depression Inventory (BDI-II), the UPDRS, as well as a past medical history and medications review. Individuals must meet all eligibility criteria in order to participate in the study. If they do not meet eligibility criteria for this study, study participation will be stopped. All procedures completed as part of the screening process are for research purposes only. If individuals continue to meet the criteria, a study team member will extract and collect de-identified clinical and demographic data already part of the patient's clinical records at the UBC Movement Disorder Clinic. This clinical data will include past medical history, disease age of onset, initial PD symptoms and medications.

A series of additional questionnaires and assessments, as well as the collection of lifestyle, health and demographic information will be completed following the screening process. Please see page 3 for the full list. Individuals' height, weight and waist circumference will also be measured.

All participants will provide a fecal sample and blood sample. The blood sample will be completed on-site, and the fecal sample will be completed at home and mailed to the research coordinator at the PPRC.

Participants may be asked to complete some of the initial visit study procedures remotely via telephone, email and/or videoconferencing prior to coming on-site for a brief in-person visit.

4.3.4. Fecal Sample

Fecal samples will be taken at home by participants. Participants will be provided with two fecal sampling kits during visit 1, each of which will include a sampling scoop, container, instructions and Bristol stool chart form to fill out. After the first visit, participants will be asked to send in their first fecal sample by mail (pre-stamped packaging to be provided at initial visit) and after the first 8-week intervention they will be asked to collect a second sample to bring with them to the second visit. During the third visit, the participants will be provided with another two fecal sampling kits; one to be mailed back after collection at home and the other to be brought back after the completion of the second 8-week intervention. To collect the samples, they will be instructed to scoop a sample of their fecal matter and place the scoop into the container. The container will be labelled with the participant's unique study ID.

4.3.5. Blood Sample

A total of 12 mL of blood will be collected by venipuncture at each visit with an evacuated tube system by a qualified study team member. Blood collection will occur at the PPRC in the UBC Movement Disorders Clinic. Proper sanitary and disinfection protocols will be followed stringently.

Participants will not receive the results of any analyses done on their samples, even in the event of unanticipated findings.

4.3.6. Study Interventions

<u>MeDi-KD</u>

In the MeDi-KD group, the participants will adhere to a modified Mediterranean-ketogenic diet. The ketogenic component of the diet will require limiting the intake of carbohydrates to about 4-10% of all calories consumed in a day, while obtaining most of the energy from healthy fats, mostly from plant-based sources (~69-82% of the daily caloric intake) and lean proteins (~10-20% of the daily caloric intake). The simplified ketogenic ratio (the ratio of fat to carbohydrates plus protein) will be gradually increased during the first two weeks from 1:1 to 2:1. The Mediterranean component of the diet will encourage the participants to consume more green leafy vegetables, nuts, and olive oil, while limiting the consumption of processed or fried food, red meat, full-fat dairy, and sweets.

<u>MeDi-MCT</u>

In the MeDi-MCT group, the participants will adhere to the Mediterranean diet as described above, but with a greater allowance for carbohydrates and natural sweets such as fresh fruits. In addition, they will be asked to take two daily doses of medium-chain triglyceride oil (MCT oil). MCT oil is extracted from coconut oil and will provide additional energy for the body.

During the first two weeks, the participants will be instructed to gradually increase their MCT oil intake until one of the following thresholds are reached:

1) The dose is not tolerable
2) The limit of the recommended dose on the supplements' label has been reached (i.e., <40 mL of MCT oil supplement/day).
3) The blood ketone levels have surpassed the minimum threshold for being clinically relevant (0.3 mM/L) as determined by weekly self-administered blood ketone measurements.

The MCT oil supplement that will be used in this study is Nutiva MCT oil (Nutiva Inc.). This product is approved by Health Canada (NPN: 80086912) and will be used according to its approved condition of use (i.e., source of medium-chain fatty acids which supports energy production in the body at a cellular level [ATP]). Three tablespoons of Nutiva MCT oil (15mL each tablespoon) can be taken per day with the recommended first-time dose of 5 mL (one teaspoon). Each serving (one tablespoon) of this product provides 130 Calories from MCTs (14 g) with a C8-C10 ratio of 60:40. Nutiva MCT oil can be mixed into any beverage of choice but cannot be used for cooking.

All participants will start the intervention by taking 1 teaspoon (5 mL) of MCT oil twice daily for the first day to acclimate their bodies to the supplement. The dose will be increased to 2 teaspoons twice daily on day 2 and 1 tablespoon (15 mL) twice daily on the subsequent days of the week. Blood ketone levels will be measured at the end of week one; if tolerability of the supplement remains positive and if the blood ketone level of 0.3 mM/L has not been reached, the participant will be asked to increase the dose to 20 mL of MCT twice daily. The MCT oil can be mixed into coffee or shakes.

4.3.7. Participant Monitoring

Phone check-ins will be completed at the week 4 mark of each intervention phase. Compliance and adverse events will be queried at each check-in and at the final visit. Participants will be asked to report adverse events throughout the duration of the study to the research team and will be provided with the relevant contact information (telephone numbers). Additionally at each check-in participants will be asked to remotely complete various questionnaires as outlined in the study visit summary with the exception of the compliance survey that will be filled in by the study coordinator based on participant report. Lastly, approximately 30 days after the final visit a phone check-in will be completed with participants.

4.3.8. Subsequent Study Visit

<u>Visit 2/Postintervention Visit 1</u>

Following the completion of the first 8-week intervention, the participant will be asked to complete a second study visit (Visit 2/postintervention visit 1). Some of these visit procedures again may be completed remotely by accessing our electronic data capture system, by telephone or videoconferencing, prior to coming to the clinic for a brief in-person portion of the visit. The second stool sample will be collected after the participant's first bowel movement following completion of the 8-week intervention and brought with them to the final visit. The participants will be asked to complete a series of questionnaires, anthropometric assessments, clinical assessments and provide a second blood sample. Moreover, they will be asked to bring all study supplement boxes with all used and unused bottles to the in-person visit (if they started the study in the MeDi-MCT arm). At the end of the second visit overall compliance to the intervention will be assessed (see 4.3.9 for details) and they will be asked about the feasibility and barriers to following the respective dietary intervention.

Washout period

After completing the second study visit, the participants will start the 8-week washout period. During the washout period they will return to the same lifestyle they were following prior to the start of the study. We will conduct phone check-ins at week 4 of the washout period. There will be no in-person visits scheduled for the washout period.

Visit 3/pre-intervention visit 2

Following the washout period, the participants will be asked to complete another in person visit, which will be very similar in nature to the initial study visit. Namely, they will be asked to provide another blood sample, complete a number of questionnaires, anthropometric assessments, and bring back the fecal sample that you have previously collected at home.

During this visit, they will be assigned to the other arm of the study to complete another 8-week intervention. MCT oil supply will be provided to those who will start the MeDi-MCT intervention. All participants will be provided with another study journal, which they will use to track their daily intake of the MCT oil supplement (those in the MeDi-MCT arm), bowel movements, one day sample weekly records of their food intake, and any adverse reactions.

Final visit/post-intervention visit 2

Within one week of completion of the second 8-week intervention, the participants will be asked to complete a final visit. Similar to before, they will complete a series of questionnaires, anthropometric assessments, clinical assessments, provide a blood sample, and bring back the previously collected fecal sample.

Postintervention visits will be scheduled within the 8th week of the respective intervention period. Some of these visit procedures again may be completed remotely by accessing our electronic data capture system, by telephone or videoconferencing, prior to coming to the clinic for a brief in-person portion of the visit. The postintervention stool samples will be collected after the first bowel movement following the completion of the 8-week intervention and brought to the visit.

Approximately 30 days after the final study visit, the research coordinator will check in with participants to see how participants are doing.

4.3.9. Assessment of Compliance

The overall compliance will be assessed at the check-ins (week 4 of each intervention phase) and recorded by the study coordinator via compliance surveys. This is intended to encourage compliance and provide information on compliance over the duration of the intervention. In case of poor compliance (< 70% adherence to treatment) assessed at week 4, the research coordinator will call the participant to develop strategies for improvement. If the participant is unable to improve compliance to at least 70% they may be discontinued from the study.

Moreover, the level of ketosis will be self-monitored by the participants at home and on a weekly basis using the commercially available Freestyle Precision Neo device (Abbot). Participants will be provided

with a Freestyle Lancing device II and lancets (Abbot), which they use to prick their finger and obtain a small drop of blood for the test. The Freestyle Precision Neo has a built-in port for test strips which can be used for monitoring blood BHB levels using Freestyle Precision blood ketone strips. All material needed for the test will be provided to the participants. Participants will be educated on using the device during the study visits and ongoing support will be provided during the weekly check-ins.

Previous studies have found blood BHB levels to be more accurate in predicting benefits of ketosis as compared to daily urine ketone monitoring, even if blood analyses are done less frequently [53, 54].

Participants will also keep diaries on stool habits using the Bristol Stool scale to rate stool composition as well as capturing frequency and other clinically relevant information, in addition to tracking their intake of the MCT supplement.

4.3.9.1. Dietary compliance

Dietary adherence will be fostered with a multi-pronged strategy following manuals and standard operating procedures similar to prior large scale, successful dietary interventions [28,55]. During both treatment interventions participants will have equal contact time with a registered dietitian. Before the first in-person visit, participant will be asked to complete a Nutrition Information Form that contains questions on participants habitual dietary patterns and other nutrition related information. Each participant will have one in-person session with a registered dietitian at the beginning of each intervention at the pre-intervention visit, with a virtual follow up within the first week of intervention. Weekly check-ins will be conducted virtually for the duration of each intervention thereafter to ensure a continuous and motivated adherence to the dietary interventions. To encourage adherence to the protocol and to successfully address barriers and challenges, individual sessions are standardized but allow for individualization given the needs of the participant. Participants will be able to make individualized weekly meal plan with provided recipes and receive coaching by a registered dietitian using motivational counselling techniques. Extra sessions with a registered dietitian can be provided to participants who are facing urgent dietary issues or questions. The content of extra sessions will be documented and discussed.

Additionally, participants will also be asked to fill in a 3-day food record at the beginning of the intervention (capturing two weekdays and a weekend day) and pseudo-random one-day records at weekly intervals to ensure adherence and successful implementation of the respective protocol. The Participants will also be asked to fill in the MIND Diet Score [83] and Mediterranean Diet Adherence Screener [84] at each pre-intervention and post-intervention visit to assess the adherence and the effect of the interventions on dietary patterns.

Ajinomoto Cambrooke Inc, a therapeutic nutrition company provided two Ketogenic Diet Professional Training sessions to our registered dietitian and provided a gram scale to each participant to assist them in following the recipe.

4.3.9.2. MCT-Supplement Adherence

In addition to strategies mentioned above (i.e., check-ins with the dietitian, blood ketone monitoring, and filling daily diaries) the participants will be requested to bring all study supplement boxes and bottles (used and unused) to the final visit where overall compliance will be determined by bottle count.

4.3.10. Premature Withdrawal/Discontinuation Criteria

Participation is completely voluntary. Participants may withdraw at any time without giving reasons, without any change to the care they will receive from their doctor, and without any loss of benefits to which they are otherwise entitled.

If they choose to enter the study and then decide to withdraw at a later time, all information about them collected up to the point of their withdrawal including, where applicable, information obtained from their biological samples will be retained for analysis in order to protect the integrity of the research, which may benefit future research participants and patients. However, no further information will be collected.

If samples have been collected before a participant withdraws, they will be asked if they wish to have their samples destroyed to prevent further testing. Samples from withdrawn participants will only be kept with their permission. There may be exceptions where the samples will not be able to be withdrawn for example where the sample is no longer identifiable (meaning it cannot be linked in any way back to their identity).

If a study participant no longer meets inclusion/exclusion criteria at any time throughout the study duration, the principal investigator may consider early discontinuation of the study intervention (i.e. BDI-II score above 28).

4.3.11. Rescue Medication

There are no specific antidotes or medications for unexpected effects associated with MCT supplements. In the event of any gastrointestinal upset, the use of over-the-counter medications (i.e. antacids, laxatives, loperamide) may be recommended by the study investigator.

4.3.12. Washout Period

Participants will continue with their usual baseline diet. This will not be standardized but we will capture baseline dietary intake with the food frequency questionnaire at the first visit. Dietary changes lead to gut microbiome changes within days [55], therefore, the 8-week washout period should be sufficient to avoid any carry-over effects; nonetheless, we will assess potential carry-over effects of diet order in the analysis.

4.3.13. Concomitant Medication

There is no limitation on concomitant medication while taking The MCT supplement. However, we include the following drug exclusion criteria for our study:

- Probiotic usage in the last 4 weeks prior to the trial
- Antibiotic usage in the last 3 months prior to the trial
- Regular benzodiazepines usage
- Use of immunomodulatory agents
- Change in anti-depressant within in the last 4 weeks
- Change in Parkinson medication within the last 2 weeks

- Use of MCT oil or ketogenic diet in the last 8 weeks, prior to the trial

4.4. Study Monitoring

Study monitoring will be conducted on a regular and as-needed basis by an experienced and qualified clinical research associate (CRA). Monitoring will follow a risk-based approach with a focus on participant safety and investigational product accountability. On-site monitoring visits will include, but are not limited to review of participant informed consent forms, adverse events/serious adverse events documentation and reporting, investigational product accountability, and source document verification.

5. Analysis

5.1. Biological sampling and analysis

We will collect blood and faecal samples at each assessment. The nature of the proof-of-concept study and inherent budget constraints require limitation to the most immediately informative markers. Additional samples will be collected (serum, plasma, erythrocytes) for future analysis such as metabolomics, lipidomics, neurofilament light chain in serum, vitamins, polyunsaturated fatty acids.

5.1.1. Blood sampling:

Participants will fast overnight before the study visit, come to the lab in the morning where they have breakfast (and if on the MeDi-MCT arm their morning MCT oil), one to two hours after the MCT intake/breakfast, blood samples will be collected.

5.1.2. Fecal sampling

After collection, a portion will be suspended in a buffer and another freshly frozen without buffer. Participants will aim to collect the sample in the 24 hours before coming for their assessment visits, place the freshly frozen samples into their home freezer immediately after collection and transport the sample on ice to our lab where it will be stored at -80 degrees Celsius. Alternatively, faecal samples can be couriered on ice.

5.1.3. Fecal sample analysis

The fecal samples will be analyzed for the following:

Microbiome readout: We will perform shotgun metagenomics on the Illumina NextSeq 500 platform using the DNA extracted from fecal samples. Microbiome markers will include relative counts of specific taxa including Akkermansia, diversity, functional pathways, and pro-/anti-inflammatory propensity.

DNA extraction, library preparation, sequencing, and processing will follow locally established procedures. In short, DNA is extracted with a KingFisher robot using the Qiagen MagAttract PowerSoil DNA KF kit (Formerly MO Bio PowerSoil DNA Kit). Following quality control, libraries are prepared with the Illumina Nextera library preparation kit (Illumina, San Diego, CA, USA). Paired-end sequencing (150 bp x 2) is done on an Illumina NextSeq 500.

Shotgun metagenomic sequence reads are processed with the Sunbeam pipeline. Processing includes adapter removal, read trimming, low-complexity-reads removal, and host-sequence removals with referencing to the Genome Reference Consortium Human Reference 37. The remaining reads are

taxonomically classified with the MiniKraken2_v1 database [56]. For functional profiling, high-quality (filtered) reads are aligned against the SEED database via translated homology search and annotated to Subsystems, or functional levels, 1-3 using Super-Focus [57].

Fecal SCFA content: SCFA analysis (including butyrate, acetate, propionate) will be carried out in the freshly frozen faecal samples, using a well-established gas chromatography-mass spectrometry (GC-MS) protocol [58]. In brief, 1 mL of ice-cold 10 % isobutanol is added to 100-150 mg of fecal sample to extract SCFAs from fecal samples. The supernatant is processed and labeled by isobutyl chloroformate. The labeled SCFA samples will be loaded on an Agilent GC-MS system for SCFA analysis.

Calprotectin and Zonulin: A consistent feature of gut microbiome dysbiosis in PD is an increase in intestinal inflammatory markers such as Calprotectin and increased intestinal permeability markers such as zonulin [6,59,60]. Both markers are commonly used for diagnosis and assessment of inflammatory bowel disease (IBD) and irritable bowel syndrome (IBS), which are risk factors for development of PD [59]. We will measure both faecal and blood-based markers. Zonulin will be measured with a competitive binding enzyme-linked immunosorbent assay (ELISA) [59], calprotectin will be measured with a two-site sandwich ELISA [61].

5.1.4. Blood sample analysis

The blood samples will be analyzed for the following:

Ketone bodies:

Continuous monitoring of blood BHB levels will be done using the Freestyle Precision Neo device on a weekly basis. The device is compatible with the Freestyle Precision blood ketone strips. Freestyle Lancing Device II and Freestyle lancets will be used to prick a finger and obtain a small drop of blood. The measurements will be digitally stored in the reader. The participants will be asked to bring the reader with themselves to the respective post-intervention study visit, where study team members can extract the data. Blood BHB levels, which are the most accurate form of determining the level of ketosis, will also be measured using the blood samples obtained during the study visits [54].

Inflammatory biomarkers: Inflammation, both systemic and neuroinflammation are strongly implicated in the pathophysiology of PD and increased inflammation is associated with faster disease progression [62]. Dietary patterns can act pro-inflammatory such as the typical “Western diet” [63] or anti-inflammatory such as Mediterranean diets [64]. In PD, higher baseline blood C-reactive protein (CRP) levels are associated with shorter survival [65] and faster motor progression [66].

We will perform the high sensitivity CRP (hsCRP) test to measure the levels of CRP. The blood samples will also be analyzed for 46 blood cytokine and chemokine analytes, using electrochemiluminescence assays from MesoScale Discovery, a high sensitivity assay kit.

5.2. Demographic and Clinical Information

We will collect detailed clinical and demographic information to maximize insights from this trial. All assessments will be collected in the ON state.

Demographics data will include information regarding the participants age, sex, race, ethnicity, education, occupational history, and marital status.

Clinical data will include information on the participants' height, weight, waist circumference, blood pressure, past medical history, family history, mode of birth, and a list of medications and supplements currently in use.

Additionally, the following medical questionnaires will be administered for the outlined reason:

- Movement Disorder Society – Unified Parkinson's Disease Rating Scale (MDS-UPDRS) for PD severity [67]
- Fatigue Severity Scale (FSS) for measuring Fatigue in PD patients [68]
- Starkstein Apathy Scale (AS) for measuring Apathy in PD patients [69]
- Beck Depression Inventory II (BDI-II) for measuring symptoms of depression [70]
- Parkinson's Anxiety Scale (PAS) [71]
- Parkinson's disease questionnaire (PDQ-39) for assessing quality of life in PD patients [72]
- Physical Activity Scale for Individuals with Physical Disabilities (PASIPD) [73]
- Bristol Stool Chart for assessing stool consistency [74]
- Rome III for constipation and irritable bowel symptoms [75]
- Patient Global Impression of Change (PGIC) for impression of change due to the interventions
- WHO Quality of life questionnaire for general quality of life
- The Canadian version of the Diet History questionnaire (C-DHQ II) [76] for dietary habits prior to beginning of the study.
- The Parkinson's Disease Cognitive Rating Scale (PD-CRS) [77]
- The Parkinson's Disease Cognitive Functional Rating Scale (PD-CFRS) [78]

5.3. Statistical Analysis

We will employ repeated measures analysis of covariance (ANCOVA) where time (pre versus post) will be the repeated factor, and age, sex, PD duration, levodopa-equivalent dose, use of entacapone [16] and order of diet intervention will be covariates. Covariates that do not significantly contribute will be dropped from the model. We will perform separate repeated measures analyses for each diet intervention (pre and post MeDi-KD and Medi-MCT, respectively) and will correct for multiple comparisons. The primary outcome analysis will be based on intention-to-treat. Datasets will also be analyzed segregated by sex on an explorative basis. Functional analysis of altered microbiome profiles will be used to understand the biological mechanisms triggered by the respective dietary interventions. In-depth unsupervised analyses of clinical phenotypes, microbiome features, and inflammatory markers will be used to identify empirical groupings among features, interventions and patients.

6. Adverse Event Reporting

Study physicians and the research staff will monitor the study procedures for this trial for overall safety and scientific relevance on an ongoing basis. Study investigators will evaluate every adverse event for safety and causality. The investigator will report the following types of AEs to the Clinical Research Ethics

Board: a) serious AND unanticipated AND possibly, probably or definitely related events; b) anticipated adverse events occurring with a greater magnitude or frequency than expected; and c) other unanticipated problems involving risks to subjects or others. These adverse events or unanticipated problems involving risks to subjects or others will be reported to the CREB within 48 hours of it becoming known to the investigator. Serious adverse events and serious unexpected adverse events will also be reported to the Natural and Non-prescription Health Products Directorate (NNHPD) in an expedited manner.

6.1. Adverse Event Definitions

6.1.1. Adverse Event (AE):

An adverse event (AE) is defined as any adverse change from the participant's baseline condition, regardless of relationship to study drug, including clinical or laboratory tests, or abnormalities which occur after informed consent is signed and up to 30 days after the study product has been discontinued. Clinically significant adverse changes in clinical status and physical examinations are considered AEs. Any participant complaint associated with such an abnormal finding will also be reported as an AE. AEs/SAEs will be collected for all untoward occurrences after ICF is signed.

Adverse events include but are not limited to: (1) worsening or change in nature, severity, or frequency of conditions or symptoms present at the start of the study; (2) participant deterioration due to primary illness; (3) intercurrent illness; and (4) drug interaction. An abnormal laboratory value will only be reported as an AE it if requires therapeutic medical intervention, if the investigator considers it to be an AE, or if it leads to the participant being withdrawn from the study.

The investigator should attempt to establish a diagnosis of the event based on signs, symptoms, and/or other clinical information. In such cases, the diagnosis should be documented as the AE and not the individual signs/symptoms. Symptoms and conditions present at the beginning of the study will be characterized, so that AEs can be defined as any new symptom, or any increase in frequency or severity of an existing symptom.

Following questioning and evaluation, all AEs, whether determined to be related or unrelated to the study drug by the Site Protocol Principal Investigator, must be documented in the participant's medical records, in accordance with the investigator's normal clinical practice. Each AE is evaluated for duration, severity, seriousness, and causal relationship to the study drug.

6.1.2. Serious Adverse Event (SAE):

Any untoward medical occurrence that at any dose: results in death, is life-threatening, require inpatient hospitalization or prolongation of existing hospitalization, results in persistent or significant disability/incapacity, or is a congenital anomaly/birth defect.

Medical and scientific judgment will be exercised in deciding whether expedited reporting in addition to the above is appropriate in other situations, such as important medical events that may not be immediately life-threatening or result in death or hospitalization but may jeopardize the subject or may

require intervention to prevent one of the outcomes listed in the definition above. Hospitalizations that fulfill one of the following conditions will not have to be reported as an SAE:

- Hospitalizations for social reasons and thus unrelated to a deterioration of the subject's condition or adverse event (e.g., deterioration of the living conditions related to environmental factors rather than to a deterioration of the disease, lack of transportation to the investigational site, respite care for the caregiver)
- Hospitalizations for elective surgical interventions for which the date had already been determined prior to the study participation.

6.1.3. Unlisted (Unexpected) Adverse Event:

An adverse event, the nature or severity of which is not consistent with the applicable product information (e.g. package insert/summary of product characteristics for an approved product (ICH and GCP)

6.1.4. Life-Threatening:

Any event in which the subject was at risk of death at the time of the event; it does not refer to an event, which hypothetically might have caused death if it were more severe.

6.1.5. Associated with the Use of the Drug:

An adverse event is considered associated with the use of the drug if the attribution is possible, probable, or very likely.

7. Trial Conduct

This study will be conducted in compliance with the protocol approved by the University of British Columbia Clinical Research Ethics Board (UBC CREB), and according to Good Clinical Practice standards 73. No deviation from the protocol will be implemented without the prior review and approval of the Ethics committee where it may be necessary to eliminate an immediate hazard to a research subject. In such case, the deviation will be reported to the UBC CREB as soon as possible.

8. Maintenance/Database

8.1. Confidentiality and Data Storage

Each participant will be assigned a unique study ID upon entrance into the study. This number will not include any personally identifying information. Only this number will be used on any research-related information collected from participants, including bio-specimens. Only the PI and REB-approved study team members will have access to the key that links participant study numbers to their identifying information. Clinical data collected from patient medical records will be de-identified upon extraction.

Data will be stored on Research Electronic Data Capture System (REDCap) as well as on password protected and encrypted computerized files on personal computers at the Djavad Mowafaghian Centre for Brain Health (DMCBH). Hard copies of participant source documents will be stored in a restricted-access office.

Fecal and blood samples will be stored at -80 C in a restricted-access refrigerator at DMCBH before analysis at the Michael Smith Laboratories and Wellington Laboratory at UBC. During analysis, all samples will be stored in restricted-access rooms and freezers. All samples will be destroyed in a confidential manner at the end of the study in their respective locations.

8.2. Source Data/Documentation

The site will maintain appropriate medical and research records for this trial and institutional requirements for the protection of confidentiality of study participants. The Principal Investigator is responsible for assuring that the data collected are complete, accurate, and recorded in a timely manner.

Study data will be collected on study specific case report forms (CRFs). The only patient identifier that will appear on CRFs is a unique assigned patient number assigned by the randomization system. The map between patient name and patient number will be maintained by the site. Source documentation should support the data collected on the CRFs. Source documents include all recordings of observations or notations of clinical activities and all reports and records necessary for the evaluation and reconstruction of the clinical trial. Data for CRFs will be collected during patient visits, phone calls with participants and health care providers, patient diaries, and completed questionnaires and abstracted from the participant's participation in the trial. Both the original records and the data extracts will be retained for period of 25 years as per Good Clinical Practice (GCP) guidelines.

8.3. Ethical Consideration

This study will be conducted according to Canadian and international standards of GCP for all studies. Applicable government regulations and research policies and procedures of the University of British Columbia will also be followed.

This protocol and any amendments will be submitted to the UBC Clinical Research Ethics Boards (CREB) for formal approval to conduct the study. The decision of the CREB concerning the conduct of the study will be made in writing to the investigator.

All subjects for this study will be provided a consent form describing this study and providing sufficient information for subjects to make an informed decision about their participation in this study. This consent form will be submitted with the protocol for review and approval by the CREB. The formal consent of a subject, using the CREB-approved consent form, will be obtained before that subject is submitted to any study procedure. This consent form will be signed by the subject or their legally acceptable surrogate, and the investigator-designated research professional obtaining the consent.

9. Risks

9.1. MCT Supplementation

MCT supplements used in this trial are Health Canada approved Natural Health Products and therefore, are regarded as safe within the approved condition of use. The approved condition of use for MCT supplements is to be used as a source of energy in dietary regimens where caloric intake is restricted.

The most common associated side effect of MCT oil is gastrointestinal upset, which are typically mild and transient in nature and can be treated with over the counter (OTC) medication and stopping the supplement. We will account for this by continuously monitoring the participants and providing them with symptoms diaries. Moreover, the dosage of the MCT supplement will be titrated during the first week of the study by gradually increasing the dose from 10 to 40 millilitres of MCT oil per day.

MCT oil is high in saturated fat (14 g of saturated fats per 15 mL of oil) and its consumption in excess may increase the risk of developing coronary heart disease. Previous studies have determined that 50 g of MCT per day is safe and well tolerated by the participants [79].

9.2. The Mediterranean-Ketogenic Diet

MeDi-KD is not associated with any significant risks. KDs in general rely on fats as the primary source of calories. High consumption of animal-based fats can be associated with an increased risk of developing coronary heart disease [18]. Nonetheless, the majority of the fats in the Mediterranean-Ketogenic diet are derived from plant-based sources to reduce the risk of hypercholesterolemia and other blood lipid abnormalities.

Moreover, classical KDs are associated with alterations withing the gut microbiome, including a reduction in butyrate SCFA [50] and a reduction in the microbial diversity of the gut [49,80]. Although it has not yet been established whether these alterations are harmful or not, the MeDi-KD circumvents these potential risks by allowing for a higher intake of vegetables, particularly those rich in fiber, which are essential for production of SCFAs including butyrate. Previous studies investigating the MeDi-KD in AD did not observe any changes in the microbial diversity of the gut [48].

We will monitor the patient participants' symptoms closely through regular check-ins and symptoms diaries. Particularly, we will actively monitor for gastrointestinal symptoms and prurigo pigmentosa ("keto rash") – an inflammatory skin condition associated with KDs – which, responds to treatment with an increase in carbohydrate intake and antibiotics [81].

9.3. Venipuncture

There are physical risks associated with collection of a blood sample. These are generally considered minimal in risk. Risks include bruising in the area of venipuncture, physical pain caused by the needle, possible exposure to infection, and emotional discomfort that might accompany the blood draw. Research personnel will do what they can to make the participant comfortable. Proper cleanliness and disinfection procedures will be followed stringently for every procedure.

9.4. Blood Ketone Self-monitoring

There might be slight discomfort associated with the weekly blood ketone self-monitoring tests, as the participants will be asked to use the lancing device to prick their fingertips and obtain a small drop of blood. Nonetheless, we anticipate that this potential discomfort will be minor in nature, as the Freestyle Lancing device II that we will provide utilizes a patented comfort zone technology, which has shown to reduce the pain associated with finger pricking.

9.5. Non-physical risks

There is a non-physical risk associated with possible disclosure of clinical data, which could result in discrimination by employers, insurance providers, and others. There are many safeguards in place to prevent this from occurring, so the risk is estimated to be small.

10. Benefits

Numerous interconnected pathological mechanisms contribute to PD including inflammation, oxidative stress, accumulation of alpha-synuclein aggregates, mitochondrial dysfunction and neuronal energy failure, brain insulin-resistance, changes in lipid metabolism, and lack of neurotrophic factors [82]. In order to develop neuroprotective therapies (which the field of PD sorely lacks), multi-pronged approaches simultaneously addressing several pathways implied in PD are likely required. Dietary interventions such as the Mediterranean diets (MeDi) and ketogenic diets (KD) provide such a multi-pronged approach with promising emerging evidence for each and some distinct mechanisms of action.

This study will harness the benefits of both the MeDi-KD and the MeDi-MCT diets, neither of which has been investigated in PD before. Furthermore, microbiome-based and microbiome-targeting therapies have the potential to provide novel mechanisms of action and if applied early enough to have preventive effects. Diet is the strongest modulator of the gut microbiome and insights from this study have the potential to foster the development of synergistic nutritional and microbiome-based therapies.

11. Reimbursement

Participants will be provided with pre-paid packaging for mailing of their fecal samples to the PPRC. There will be no costs to the participants. Participants will receive some remuneration depending on where participants will be travelling from (minimum of $30). See below for details:

- Vancouver Metro Region = $30 per visit
- Fraser Valley Region= $45 per visit
- Vancouver Island/Kootenay-Boundary/Northern/Thompson-Okanagan Regions = $75 per visit

12. Significance

The study is part of our team's larger program into synergistic lifestyle and microbiome-based interventions in brain disorders. It will de-risk future studies on ketogenic interventions by providing vital information about the safety, tolerability, adherence, and feasibility of the MeDi-KD and MeDi-MCT. We will determine if the ketogenic interventions in PD are safe or associated with harmful gut inflammation, reduction of butyrate/SCFA levels, systemic inflammation, gut barrier compromise or dysbiosis, particularly with respect to a potential Akkermansia bloom. We will understand if the MeDi-KD and the MeDi-MCT can elevate blood ketone bodies to clinically relevant levels, while remaining feasible and safe, while valuing the participants' perspective.

By the end of the project, we will weigh safety, feasibility and exploratory efficacy signals to determine which intervention to pursue in larger clinical trials. With this information, we plan to conduct a larger clinical trial to assess clinical benefits in the most promising intervention with more elaborate data collection (e.g., brain imaging information)

13. Reference

1. GBD 2016 Parkinson's Disease Collaborators. Global, regional, and national burden of Parkinson's disease, 1990-2016: a systematic analysis for the Global Burden of Disease Study 2016. Lancet Neurol 2018 Nov;17(11):939–953. PMID:30287051

2. Dorsey ER, Sherer T, Okun MS, Bloem BR. The Emerging Evidence of the Parkinson Pandemic. J Parkinsons Dis 2018;8(s1):S3–S8. PMID:30584159

3. van der Kolk NM, de Vries NM, Kessels RPC, Joosten H, Zwinderman AH, Post B, Bloem BR. Effectiveness of home-based and remotely supervised aerobic exercise in Parkinson's disease: a double-blind, randomised controlled trial. Lancet Neurol 2019 Nov;18(11):998–1008. PMID:31521532

4. Borghammer P, Van Den Berge N. Brain-First versus Gut-First Parkinson's Disease: A Hypothesis. J Parkinsons Dis 2019;9(s2):S281–S295. PMID:31498132

5. Sampson TR, Debelius JW, Thron T, Janssen S, Shastri GG, Ilhan ZE, Challis C, Schretter CE, Rocha S, Gradinaru V, Chesselet M-F, Keshavarzian A, Shannon KM, Krajmalnik-Brown R, Wittung-Stafshede P, Knight R, Mazmanian SK. Gut Microbiota Regulate Motor Deficits and Neuroinflammation in a Model of Parkinson's Disease. Cell 2016 Dec 1;167(6):1469–1480.e12. PMID:27912057

6. Aho VTE, Houser MC, Pereira PAB, Chang J, Rudi K, Paulin L, Hertzberg V, Auvinen P, Tansey MG, Scheperjans F. Relationships of gut microbiota, short-chain fatty acids, inflammation, and the gut barrier in Parkinson's disease. Mol Neurodegener 2021 Feb 8;16(1):6. PMID:33557896

7. Hirayama M, Ohno K. Parkinson's Disease and Gut Microbiota. Ann Nutr Metab 2021 Sep 9;1–8. PMID:34500451

8. Boertien JM, Pereira PAB, Aho VTE, Scheperjans F. Increasing Comparability and Utility of Gut Microbiome Studies in Parkinson's Disease: A Systematic Review. J Parkinsons Dis 2019;9(s2):S297–S312. PMID:31498131

9. Cirstea MS, Yu AC, Golz E, Sundvick K, Kliger D, Radisavljevic N, Foulger LH, Mackenzie M, Huan T, Finlay BB, Appel-Cresswell S. Microbiota Composition and Metabolism Are Associated With Gut Function in Parkinson's Disease. Mov Disord 2020 Jul;35(7):1208–1217.

10. Wallen ZD, Appah M, Dean MN, Sesler CL, Factor SA, Molho E, Zabetian CP, Standaert DG, Payami H. Characterizing dysbiosis of gut microbiome in PD: evidence for overabundance of opportunistic pathogens. NPJ Parkinsons Dis 2020 Jun 12;6:11. PMID:32566740

11. Barichella M, Severgnini M, Cilia R, Cassani E, Bolliri C, Caronni S, Ferri V, Cancello R, Ceccarani C, Faierman S, Pinelli G, De Bellis G, Zecca L, Cereda E, Consolandi C, Pezzoli G. Unraveling gut

microbiota in Parkinson’s disease and atypical parkinsonism. Mov Disord 2019 Mar;34(3):396–405. PMID:30576008

12. Nishiwaki H, Ito M, Ishida T, Hamaguchi T, Maeda T, Kashihara K, Tsuboi Y, Ueyama J, Shimamura T, Mori H, Kurokawa K, Katsuno M, Hirayama M, Ohno K. Meta-analysis of gut dysbiosis in Parkinson’s disease. Mov Disord Wiley; 2020 Sep;35(9):1626–1635. PMID:32557853

13. Scheperjans F, Aho V, Pereira PAB, Koskinen K, Paulin L, Pekkonen E, Haapaniemi E, Kaakkola S, Eerola-Rautio J, Pohja M, Kinnunen E, Murros K, Auvinen P. Gut microbiota are related to Parkinson’s disease and clinical phenotype. Mov Disord 2015 Mar;30(3):350–358. PMID:25476529

14. Keshavarzian A, Green SJ, Engen PA, Voigt RM, Naqib A, Forsyth CB, Mutlu E, Shannon KM. Colonic bacterial composition in Parkinson’s disease. Mov Disord 2015 Sep;30(10):1351–1360. PMID:26179554

15. Unger MM, Spiegel J, Dillmann K-U, Grundmann D, Philippeit H, Bürmann J, Faßbender K, Schwiertz A, Schäfer K-H. Short chain fatty acids and gut microbiota differ between patients with Parkinson’s disease and age-matched controls. Parkinsonism Relat Disord 2016 Nov;32:66–72. PMID:27591074

16. Hill-Burns EM, Debelius JW, Morton JT, Wissemann WT, Lewis MR, Wallen ZD, Peddada SD, Factor SA, Molho E, Zabetian CP, Knight R, Payami H. Parkinson’s disease and Parkinson's disease medications have distinct signatures of the gut microbiome. Mov Disord 2017 May;32(5):739–749. PMID:28195358

17. Heintz-Buschart A, Pandey U, Wicke T, Sixel-Döring F, Janzen A, Sittig-Wiegand E, Trenkwalder C, Oertel WH, Mollenhauer B, Wilmes P. The nasal and gut microbiome in Parkinson’s disease and idiopathic rapid eye movement sleep behavior disorder. Mov Disord 2018 Jan;33(1):88–98. PMID:28843021

18. Włodarek D. Role of Ketogenic Diets in Neurodegenerative Diseases (Alzheimer’s Disease and Parkinson's Disease). Nutrients [Internet] 2019 Jan 15;11(1). PMID:30650523

19. Paoli A, Bianco A, Damiani E, Bosco G. Ketogenic diet in neuromuscular and neurodegenerative diseases. Biomed Res Int 2014 Jul 3;2014:474296. PMID:25101284

20. Vanitallie TB, Nonas C, Di Rocco A, Boyar K, Hyams K, Heymsfield SB. Treatment of Parkinson disease with diet-induced hyperketonemia: a feasibility study. Neurology 2005 Feb 22;64(4):728–730. PMID:15728303

21. Krikorian R, Shidler MD, Summer SS, Sullivan PG, Duker AP, Isaacson RS, Espay AJ. Nutritional ketosis for mild cognitive impairment in Parkinson’s disease: A controlled pilot trial. Clin Park Relat Disord 2019 Aug 6;1:41–47. PMID:34316598

22. Phillips MCL, Murtagh DKJ, Gilbertson LJ, Asztely FJS, Lynch CDP. Low-fat versus ketogenic diet in Parkinson's disease: A pilot randomized controlled trial. Mov Disord 2018 Aug;33(8):1306–1314. PMID:30098269

23. Choi A, Hallett M, Ehrlich D. Nutritional Ketosis in Parkinson's Disease - a Review of Remaining Questions and Insights. Neurotherapeutics [Internet] 2021 Jul 7; PMID:34235637

24. Davis C, Bryan J, Hodgson J, Murphy K. Definition of the Mediterranean Diet; a Literature Review. Nutrients 2015 Nov 5;7(11):9139–9153. PMID:26556369

25. Nagpal R, Shively CA, Register TC, Craft S, Yadav H. Gut microbiome-Mediterranean diet interactions in improving host health. F1000Res 2019 May 21;8:699. PMID:32704349

26. Alcalay RN, Gu Y, Mejia-Santana H, Cote L, Marder KS, Scarmeas N. The association between Mediterranean diet adherence and Parkinson's disease. Mov Disord 2012 May;27(6):771–774. PMID:22314772

27. Maraki MI, Yannakoulia M, Stamelou M, Stefanis L, Xiromerisiou G, Kosmidis MH, Dardiotis E, Hadjigeorgiou GM, Sakka P, Anastasiou CA, Simopoulou E, Scarmeas N. Mediterranean diet adherence is related to reduced probability of prodromal Parkinson's disease. Mov Disord John Wiley and Sons Inc.; 2019 Jan 1;34(1):48–57.

28. Agarwal P, Wang Y, Buchman AS, Holland TM, Bennett DA, Morris MC. MIND Diet Associated with Reduced Incidence and Delayed Progression of Parkinsonism in Old Age. J Nutr Health Aging Springer-Verlag France; 2018 Dec 1;22(10):1211–1215.

29. Molsberry S, Bjornevik K, Hughes KC, Healy B, Schwarzschild M, Ascherio A. Diet pattern and prodromal features of Parkinson's disease. Neurology [Internet] 2020 Aug 19; PMID:32817391

30. Metcalfe-Roach A, Yu AC, Golz E, Cirstea M, Sundvick K, Kliger D, Foulger LH, Mackenzie M, Finlay BB, Appel-Cresswell S. MIND and Mediterranean Diets Associated with Later Onset of Parkinson's Disease. Mov Disord [Internet] Wiley Online Library; 2020; Available from: https://onlinelibrary.wiley.com/doi/abs/10.1002/mds.28464

31. Freeman JM, Kossoff EH, Hartman AL. The ketogenic diet: one decade later. Pediatrics. 2007;119(3):535-543. doi:10.1542/peds.2006-2447

32. Nylen K, Likhodii S, Burnham WM. The ketogenic diet: proposed mechanisms of action. Neurotherapeutics. 2009;6(2):402-405. doi:10.1016/j.nurt.2009.01.021

33. Norwitz NG, Hu MT, Clarke K. The Mechanisms by Which the Ketone Body D-β-Hydroxybutyrate May Improve the Multiple Cellular Pathologies of Parkinson's Disease. Front Nutr. 2019;6:63. doi:10.3389/fnut.2019.00063

34. Cahill GF Jr. Fuel metabolism in starvation. Annu Rev Nutr 2006;26:1–22. PMID:16848698

35. Owen OE, Morgan AP, Kemp HG, Sullivan JM, Herrera MG, Cahill GF Jr. Brain metabolism during fasting. J Clin Invest 1967 Oct;46(10):1589–1595. PMID:6061736

36. Shimazu T, Hirschey MD, Newman J, He W, Shirakawa K, Le Moan N, Grueter CA, Lim H, Saunders LR, Stevens RD, Newgard CB, Farese RV Jr, de Cabo R, Ulrich S, Akassoglou K, Verdin E. Suppression of oxidative stress by β-hydroxybutyrate, an endogenous histone deacetylase inhibitor. Science 2013 Jan 11;339(6116):211–214. PMID:23223453

37. Gasior M, Rogawski MA, Hartman AL. Neuroprotective and disease-modifying effects of the ketogenic diet [Internet]. Behavioural Pharmacology. 2006. p. 431–439. [doi: 10.1097/00008877-200609000-00009]

38. García-Rodríguez D, Giménez-Cassina A. Ketone Bodies in the Brain Beyond Fuel Metabolism: From Excitability to Gene Expression and Cell Signaling. Front Mol Neurosci 2021 Aug 27;14:732120. PMID:34512261

39. D'Andrea Meira I, Romão TT, Pires do Prado HJ, Krüger LT, Pires MEP, da Conceição PO. Ketogenic Diet and Epilepsy: What We Know So Far. Front Neurosci 2019 Jan 29;13:5. PMID:30760973

40. Yuan X, Wang J, Yang S, Gao M, Cao L, Li X, Hong D, Tian S, Sun C. Effect of the ketogenic diet on glycemic control, insulin resistance, and lipid metabolism in patients with T2DM: a systematic review and meta-analysis. Nutr Diabetes 2020 Nov 30;10(1):38. PMID:33257645

41. Castellana M, Conte E, Cignarelli A, Perrini S, Giustina A, Giovanella L, Giorgino F, Trimboli P. Efficacy and safety of very low calorie ketogenic diet (VLCKD) in patients with overweight and obesity: A systematic review and meta-analysis. Rev Endocr Metab Disord Springer; 2020 Mar;21(1):5–16. PMID:31705259

42. Myette-Côté É, Soto-Mota A, Cunnane SC. Ketones - Potential to Achieve Brain Energy Rescue and Sustain Cognitive Health during Aging. Br J Nutr 2021 Sep 28;1–47. PMID:34581265

43. Marten B, Pfeuffer M, Schrezenmeir J. Medium-chain triglycerides. International Dairy Journal. 2006;16(11):1374-1382. doi:10.1016/j.idairyj.2006.06.015

44. Neal EG, Chaffe H, Schwartz RH, Lawson MS, Edwards N, Fitzsimmons G, Whitney A, Cross JH. A randomized trial of classical and medium-chain triglyceride ketogenic diets in the treatment of childhood epilepsy. Epilepsia 2009 May;50(5):1109–1117. PMID:19054400

45. Fortier M, Castellano C-A, St-Pierre V, Myette-Côté É, Langlois F, Roy M, Morin M-C, Bocti C, Fulop T, Godin J-P, Delannoy C, Cuenoud B, Cunnane SC. A ketogenic drink improves cognition in mild cognitive impairment: Results of a 6-month RCT. Alzheimers Dement 2021 Mar;17(3):543–552. PMID:33103819

46. Fortier M, Castellano C-A, Croteau E, Langlois F, Bocti C, St-Pierre V, Vandenberghe C, Bernier M, Roy M, Descoteaux M, Whittingstall K, Lepage M, Turcotte ÉE, Fulop T, Cunnane SC. A ketogenic drink improves brain energy and some measures of cognition in mild cognitive impairment. Alzheimers Dement 2019 May;15(5):625–634. PMID:31027873

47. Paoli A, Mancin L, Bianco A, Thomas E, Mota JF, Piccini F. Ketogenic Diet and Microbiota: Friends or Enemies? Genes [Internet] 2019 Jul 15;10(7). PMID:31311141

48. Nagpal R, Neth BJ, Wang S, Craft S, Yadav H. Modified Mediterranean-ketogenic diet modulates gut microbiome and short-chain fatty acids in association with Alzheimer's disease markers in subjects with mild cognitive impairment. EBioMedicine 2019 Sep;47:529–542. PMID:31477562

49. Olson CA, Vuong HE, Yano JM, Liang QY, Nusbaum DJ, Hsiao EY. The Gut Microbiota Mediates the Anti-Seizure Effects of the Ketogenic Diet. Cell 2018 Jun 14;173(7):1728–1741.e13. PMID:29804833

50. Ferraris C, Meroni E, Casiraghi MC, Tagliabue A, De Giorgis V, Erba D. One Month of Classic Therapeutic Ketogenic Diet Decreases Short Chain Fatty Acids Production in Epileptic Patients. Front Nutr 2021 Mar 29;8:613100. PMID:33855040

51. Ang QY, Alexander M, Newman JC, Tian Y, Cai J, Upadhyay V, Turnbaugh JA, Verdin E, Hall KD, Leibel RL, Ravussin E, Rosenbaum M, Patterson AD, Turnbaugh PJ. Ketogenic Diets Alter the Gut Microbiome Resulting in Decreased Intestinal Th17 Cells. Cell 2020 Jun 11;181(6):1263–1275.e16. PMID:32437658

52. Postuma RB, Berg D, Stern M, Poewe W, Olanow CW, Oertel W, Obeso J, Marek K, Litvan I, Lang AE, Halliday G, Goetz CG, Gasser T, Dubois B, Chan P, Bloem BR, Adler CH, Deuschl G. MDS clinical diagnostic criteria for Parkinson's disease. Mov Disord 2015 Oct;30(12):1591–1601. PMID:26474316

53. van Delft R, Lambrechts D, Verschuure P, Hulsman J, Majoie M. Blood beta-hydroxybutyrate correlates better with seizure reduction due to ketogenic diet than do ketones in the urine. Seizure 2010 Jan;19(1):36–39. PMID:19962324

54. Anderson JC, Mattar SG, Greenway FL, Lindquist RJ. Measuring ketone bodies for the monitoring of pathologic and therapeutic ketosis. Obes Sci Pract 2021 Oct;7(5):646–656. PMID:34631141

55. David LA, Maurice CF, Carmody RN, Gootenberg DB, Button JE, Wolfe BE, Ling AV, Devlin AS, Varma Y, Fischbach MA, Biddinger SB, Dutton RJ, Turnbaugh PJ. Diet rapidly and reproducibly alters the human gut microbiome. Nature 2014 Jan 23;505(7484):559–563. PMID:24336217

56. Wood DE, Lu J, Langmead B. Improved metagenomic analysis with Kraken 2. Genome Biol 2019 Nov 28;20(1):257. PMID:31779668

57. Silva GGZ, Green KT, Dutilh BE, Edwards RA. SUPER-FOCUS: a tool for agile functional analysis of shotgun metagenomic data. Bioinformatics 2016 Feb 1;32(3):354–361. PMID:26454280

58. Zheng X, Qiu Y, Zhong W, Baxter S, Su M, Li Q, Xie G, Ore BM, Qiao S, Spencer MD, Zeisel SH, Zhou Z, Zhao A, Jia W. A targeted metabolomic protocol for short-chain fatty acids and branched-chain amino acids. Metabolomics 2013 Aug 1;9(4):818–827. PMID:23997757

59. Schwiertz A, Spiegel J, Dillmann U, Grundmann D, Bürmann J, Faßbender K, Schäfer K-H, Unger MM. Fecal markers of intestinal inflammation and intestinal permeability are elevated in Parkinson's disease. Parkinsonism Relat Disord 2018 May;50:104–107. PMID:29454662

60. Dumitrescu L, Marta D, Dănău A, Lefter A, Tulbă D, Cozma L, Manole E, Gherghiceanu M, Ceafalan LC, Popescu BO. Serum and Fecal Markers of Intestinal Inflammation and Intestinal Barrier Permeability Are Elevated in Parkinson's Disease. Front Neurosci 2021 Jun 18;15:689723. PMID:34220443

61. Ohlsson B, Roth B, Larsson E, Höglund P. Calprotectin in serum and zonulin in serum and feces are elevated after introduction of a diet with lower carbohydrate content and higher fiber, fat and protein contents. Biomed Rep 2017 Apr;6(4):411–422. PMID:28413639

62. Harms AS, Ferreira SA, Romero-Ramos M. Periphery and brain, innate and adaptive immunity in Parkinson's disease. Acta Neuropathol 2021 Apr;141(4):527–545. PMID:33555429

63. Tran HQ, Bretin A, Adeshirlarijaney A, Yeoh BS, Vijay-Kumar M, Zou J, Denning TL, Chassaing B, Gewirtz AT. "Western Diet"-Induced Adipose Inflammation Requires a Complex Gut Microbiota. Cell Mol Gastroenterol Hepatol 2020;9(2):313–333. PMID:31593782

64. Chrysohoou C, Panagiotakos DB, Pitsavos C, Das UN, Stefanadis C. Adherence to the Mediterranean diet attenuates inflammation and coagulation process in healthy adults: The ATTICA Study. J Am Coll Cardiol 2004 Jul 7;44(1):152–158. PMID:15234425

65. Sawada H, Oeda T, Umemura A, Tomita S, Kohsaka M, Park K, Yamamoto K, Sugiyama H. Baseline C-Reactive Protein Levels and Life Prognosis in Parkinson Disease. PLoS One 2015 Jul 28;10(7):e0134118. PMID:26218286

66. Umemura A, Oeda T, Yamamoto K, Tomita S, Kohsaka M, Park K, Sugiyama H, Sawada H. Baseline Plasma C-Reactive Protein Concentrations and Motor Prognosis in Parkinson Disease. PLoS One 2015 Aug 26;10(8):e0136722. PMID:26308525

67. Goetz CG, Tilley BC, Shaftman SR, Stebbins GT, Fahn S, Martinez-Martin P, Poewe W, Sampaio C, Stern MB, Dodel R, Dubois B, Holloway R, Jankovic J, Kulisevsky J, Lang AE, Lees A, Leurgans S, LeWitt PA, Nyenhuis D, Olanow CW, Rascol O, Schrag A, Teresi JA, van Hilten JJ, LaPelle N, Movement Disorder Society UPDRS Revision Task Force. Movement Disorder Society-sponsored revision of the Unified Parkinson's Disease Rating Scale (MDS-UPDRS): scale presentation and clinimetric testing results. Mov Disord 2008 Nov 15;23(15):2129–2170. PMID:19025984

68. Brown RG, Dittner A, Findley L, Wessely SC. The Parkinson fatigue scale. Parkinsonism Relat Disord 2005 Jan;11(1):49–55. PMID:15619463

69. Starkstein SE, Mayberg HS, Preziosi TJ, Andrezejewski P, Leiguarda R, Robinson RG. Reliability, validity, and clinical correlates of apathy in Parkinson's disease. J Neuropsychiatry Clin Neurosci 1992 Spring;4(2):134–139. PMID:1627973

70. Beck AT, Steer RA, Brown G. Beck Depression Inventory–II. Psychol Assess [Internet]. [doi: 10.1037/t00742-000]

71. Leentjens AFG, Dujardin K, Pontone GM, Starkstein SE, Weintraub D, Martinez-Martin P. The Parkinson Anxiety Scale (PAS): development and validation of a new anxiety scale. Mov Disord 2014 Jul;29(8):1035–1043. PMID:24862344

72. Jenkinson C, Fitzpatrick R, Peto V, Greenhall R, Hyman N. The Parkinson's Disease Questionnaire (PDQ-39): development and validation of a Parkinson's disease summary index score. Age Ageing 1997 Sep;26(5):353–357. PMID:9351479

73. Washburn RA, Zhu W, McAuley E, Frogley M, Figoni SF. The physical activity scale for individuals with physical disabilities: development and evaluation. Arch Phys Med Rehabil 2002 Feb;83(2):193–200. PMID:11833022

74. Lewis SJ, Heaton KW. Stool form scale as a useful guide to intestinal transit time. Scand J Gastroenterol 1997 Sep;32(9):920–924. PMID:9299672

75. Drossman DA, Dumitrascu DL. Rome III: New standard for functional gastrointestinal disorders. J Gastrointestin Liver Dis 2006 Sep;15(3):237–241. PMID:17013448

76. Thompson FE, Subar AF, Brown CC, Smith AF, Sharbaugh CO, Jobe JB, Mittl B, Gibson JT, Ziegler RG. Cognitive research enhances accuracy of food frequency questionnaire reports: results of an experimental validation study. J Am Diet Assoc 2002 Feb;102(2):212–225. PMID:11846115

77. Pagonabarraga, J., Kulisevsky, J., Llebaria, G., García-Sánchez, C., Pascual-Sedano, B. and Gironell, A. (2008), Parkinson's disease-cognitive rating scale: A new cognitive scale specific for Parkinson's disease. Mov. Disord., 23: 998-1005. https://doi.org/10.1002/mds.22007

78. Kulisevsky J, Fernández de Bobadilla R, Pagonabarraga J, Martínez-Horta S, Campolongo A, García-Sánchez C, Pascual-Sedano B, Ribosa-Nogué R, Villa-Bonomo C. Measuring functional impact of cognitive impairment: validation of the Parkinson's disease cognitive functional rating scale. Parkinsonism and Related Disorders. 2013 Sep;19(9):812-7.

79. Nygaard HB, Kent BA, Stager S, et al. A phase 1B multiple ascending dose study of the safety, tolerability, pharmacokinetics, and pharmacodynamics of a medium chain triglyceride supplement in Alzheimer's disease: Brain energy rescue interventions to treat or delay Alzheimer's disease. Alzheimer's & Dementia. 2020;16(S10). doi:10.1002/alz.037960

80. Ma D, Wang AC, Parikh I, et al. Ketogenic diet enhances neurovascular function with altered gut microbiome in young healthy mice. Sci Rep. 2018;8(1):6670. doi:10.1038/s41598-018-25190-5

81. Mufti A, Mirali S, Abduelmula A, McDonald KA, Alabdulrazzaq S, Sachdeva M, Yeung J. Clinical manifestations and treatment outcomes in prurigo pigmentosa (Nagashima disease): A systematic review of the literature. JAAD Int 2021 Jun;3:79–87. PMID:34409375

82. Bloem BR, Okun MS, Klein C. Parkinson's disease. Lancet 2021 Jun 12;397(10291):2284–2303. PMID:33848468

83. Morris MC, Tangney CC, Wang Y, Sacks FM, Barnes LL, Bennett DA, et al. MIND diet slows cognitive decline with aging. Alzheimer's & Dementia [Internet]. 2015 Sep;11(9):1015–22. Available from: https://www.sciencedirect.com/science/article/abs/pii/S1552526015001946

84. Estruch R, Ros E, Salas-Salvadó J, Covas MI, Corella D, Arós F, et al. Primary Prevention of Cardiovascular Disease with a Mediterranean Diet Supplemented with Extra-Virgin Olive Oil or Nuts. New England Journal of Medicine [Internet]. 2018 Jun 21;378(25):e34. Available from: https://www.nejm.org/doi/full/10.1056/nejmoa1800389

14. Appendices

14.1 Montreal Cognitive Assessment (MoCA)

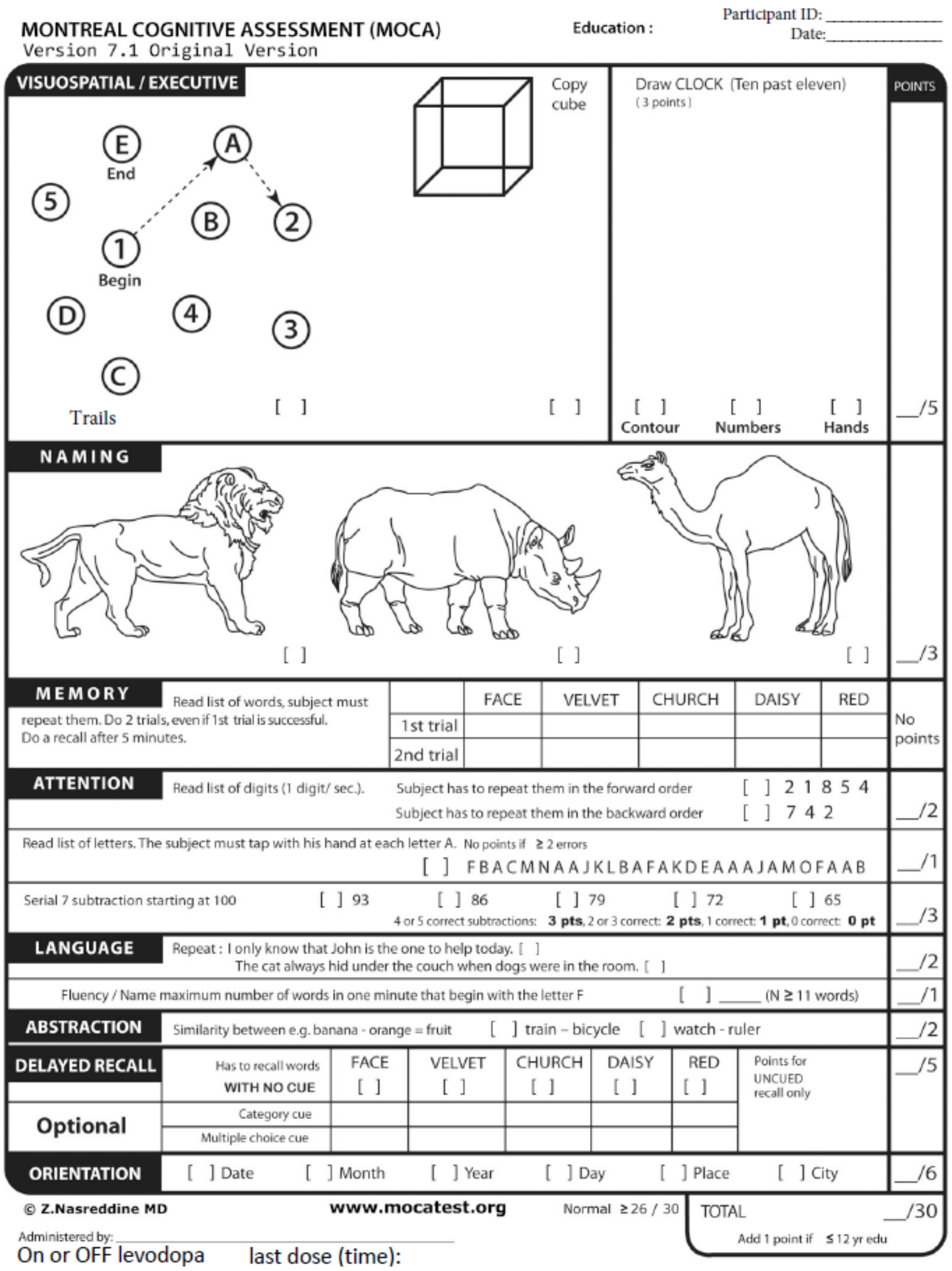

MONTREAL COGNITIVE ASSESSMENT (MOCA)
Version 7.1 Original Version

Education :

Participant ID: ______________
Date:______________

		POINTS
**VISUOSPATIAL / EXECUTIVE** — (E) End, (A), (5), (B), (2), (1) Begin, (D), (4), (3), (C) — Trails [ ] ; Copy cube [ ]	Draw CLOCK (Ten past eleven) (3 points) — [ ] Contour [ ] Numbers [ ] Hands	__/5
**NAMING** — [ ] [ ] [ ]		__/3

MEMORY Read list of words, subject must repeat them. Do 2 trials, even if 1st trial is successful. Do a recall after 5 minutes.

	FACE	VELVET	CHURCH	DAISY	RED
1st trial					
2nd trial					

No points

ATTENTION Read list of digits (1 digit/ sec.). Subject has to repeat them in the forward order [ ] 2 1 8 5 4 — Subject has to repeat them in the backward order [ ] 7 4 2 — __/2

Read list of letters. The subject must tap with his hand at each letter A. No points if ≥ 2 errors
[ ] F B A C M N A A J K L B A F A K D E A A A J A M O F A A B — __/1

Serial 7 subtraction starting at 100 [ ] 93 [ ] 86 [ ] 79 [ ] 72 [ ] 65 — __/3
4 or 5 correct subtractions: **3 pts**, 2 or 3 correct: **2 pts**, 1 correct: **1 pt**, 0 correct: **0 pt**

LANGUAGE Repeat : I only know that John is the one to help today. [ ]
The cat always hid under the couch when dogs were in the room. [ ] — __/2

Fluency / Name maximum number of words in one minute that begin with the letter F [ ] _____ (N ≥ 11 words) — __/1

ABSTRACTION Similarity between e.g. banana - orange = fruit [ ] train – bicycle [ ] watch - ruler — __/2

**DELAYED RECALL**	Has to recall words WITH NO CUE	FACE [ ]	VELVET [ ]	CHURCH [ ]	DAISY [ ]	RED [ ]	Points for UNCUED recall only	__/5
**Optional**	Category cue							
	Multiple choice cue							

ORIENTATION [ ] Date [ ] Month [ ] Year [ ] Day [ ] Place [ ] City — __/6

© Z.Nasreddine MD www.mocatest.org Normal ≥ 26 / 30 TOTAL __/30
Add 1 point if ≤ 12 yr edu

Administered by: ______________________
On or OFF levodopa last dose (time):

14.2 Movement Disorder Society - Unified Parkinson's Disease Rating Scale (MDS-UPDRS)

Patient Name or Subject ID	Site ID	(mm-dd-yyyy) Assessment Date	Investigator's Initials
________________	___ ___ ___	___ ___ - ___ ___ - ___ ___ ___ ___	___ ___ ___

MDS UPDRS Score Sheet

1.A	Source of information	☐ Patient<br>☐ Caregiver<br>☐ Patient + Caregiver	3.3b	Rigidity– RUE	
			3.3c	Rigidity– LUE	
**Part I**			3.3d	Rigidity– RLE	
1.1	Cognitive impairment		3.3e	Rigidity– LLE	
1.2	Hallucinations and psychosis		3.4a	Finger tapping– Right hand	
1.3	Depressed mood		3.4b	Finger tapping– Left hand	
1.4	Anxious mood		3.5a	Hand movements– Right hand	
1.5	Apathy		3.5b	Hand movements– Left hand	
1.6	Features of DDS		3.6a	Pronation- supination movements– Right hand	
1.6a	Who is filling out questionnaire	☐ Patient<br>☐ Caregiver<br>☐ Patient + Caregiver	3.6b	Pronation- supination movements– Left hand	
			3.7a	Toe tapping–Right foot	
1.7	Sleep problems		3.7b	Toe tapping– Left foot	
1.8	Daytime sleepiness		3.8a	Leg agility– Right leg	
1.9	Pain and other sensations		3.8b	Leg agility– Left leg	
1.10	Urinary problems		3.9	Arising from chair	
1.11	Constipation problems		3.10	Gait	
1.12	Light headedness on standing		3.11	Freezing of gait	
1.13	Fatigue		3.12	Postural stability	
**Part II**			3.13	Posture	
2.1	Speech		3.14	Global spontaneity of movement	
2.2	Saliva and drooling		3.15a	Postural tremor– Right hand	
2.3	Chewing and swallowing		3.15b	Postural tremor– Left hand	
2.4	Eating tasks		3.16a	Kinetic tremor– Right hand	
2.5	Dressing		3.16b	Kinetic tremor– Left hand	
2.6	Hygiene		3.17a	Rest tremor amplitude– RUE	
2.7	Handwriting		3.17b	Rest tremor amplitude– LUE	
2.8	Doing hobbies and other activities		3.17c	Rest tremor amplitude– RLE	
2.9	Turning in bed		3.17d	Rest tremor amplitude– LLE	
2.10	Tremor		3.17e	Rest tremor amplitude– Lip/jaw	
2.11	Getting out of bed		3.18	Constancy of rest	
2.12	Walking and balance			Were dyskinesias presen	☐ No ☐ Yes
2.13	Freezing			Did these movements interfere with ratings?	☐ No ☐ Yes
3a	Is the patient on medication?	☐ No ☐ Yes		Hoehn and Yahr Stage	
3b	Patient's clinical state	☐ Off ☐ On	**Part IV**		
3c	Is the patient on Levodopa?	☐ No ☐ Yes	4.1	Time spent with dyskinesias	
3.C1	If yes, minutes since last dose:		4.2	Functional impact of dyskinesias	
**Part III**			4.3	Time spent in the OFF state	
3.1	Speech		4.4	Functional impact of fluctuations	
3.2	Facial expression		4.5	Complexity of motor fluctuations	
3.3a	Rigidity– Neck		4.6	Painful OFF-state dystonia	

July 1, 2008

Copyright © 2008 Movement Disorder Society. All rights reserved.
This chart may not be copied, distributed or otherwise used in whole or in part without prior written consent of the Movement Disorder Society

14.3 Beck Depression Inventory, 2nd Edition (BDI-II)

ID #: ___________

Beck Depression Inventory, 2nd Edition (BDI-II)

Date: ___________

Please read each group of statements carefully, then pick out one statement in each group that best describes the way you have been feeling during the **past two weeks, including today**. Circle the number beside the statement you have picked. If several statements in the group seem to apply equally well, circle the highest number for that group. Be sure that you do not choose more than one statement for any group, including item 16 (Changes in Sleeping Pattern) or Item 18 (Changes in Appetite).

**1. Sadness**<br>0 I do not feel sad.<br>1 I feel sad much of the time.<br>2 I am sad all the time.<br>3 I am so sad or unhappy that I can't stand it.	**7. Self-Dislike**<br>0 I feel the same about myself as ever.<br>1 I have lost confidence in myself.<br>2 I am disappointed in myself.<br>3 I dislike myself.
**2. Pessimism**<br>0 I am not discouraged about my future.<br>1 I feel more discouraged about my future than I used to.<br>2 I do not expect things to work out for me.<br>3 I feel my future is hopeless and will only get worse.	**8. Self-Criticalness**<br>0 I don't criticize or blame myself more than usual.<br>1 I am more critical of myself than I used to be.<br>2 I criticize myself for all of my faults.<br>3 I blame myself for everything bad that happens.
**3. Past Failure**<br>0 I do not feel like a failure.<br>1 I have failed more than I should have.<br>2 As I look back, I see a lot of failures.<br>3 I feel I am a total failure as a person.	**9. Suicidal Thoughts or Wishes**<br>0 I don't have any thoughts of killing myself.<br>1 I have thoughts of killing myself, but would not carry them out.<br>2 I would like to kill myself.<br>3 I would kill myself if I had the chance.
**4. Loss of Pleasure**<br>0 I get as much pleasure as I ever did from the things I enjoy.<br>1 I don't enjoy things as much as I used to.<br>2 I get very little pleasure from the things I used to enjoy.<br>3 I can't get any pleasure from the things I used to enjoy.	**10. Crying**<br>0 I don't cry any more than I used to.<br>1 I cry more than I used to.<br>2 I cry over every little thing.<br>3 I feel like crying, but I can't.
**5. Guilty Feelings**<br>0 I don't feel particularly guilty.<br>1 I feel guilty over many things I have done or should have done.<br>2 I feel quite guilty most of the time.<br>3 I feel guilty all of the time.	**11. Agitation**<br>0 I am no more restless or wound up than usual.<br>1 I feel more restless or wound up than usual.<br>2 I am so restless or agitated that it's hard to stay still.<br>3 I am so restless or agitated that I have to keep moving or doing something.
**6. Punishment Feelings**<br>0 I don't feel I am being punished.<br>1 I feel I may be punished.<br>2 I expect to be punished.<br>3 I feel I am being punished.	**12. Loss of Interest**<br>0 I have not lost interest in other people or activities.<br>1 I am less interested in other people or things than before.<br>2 I have lost most of my interest in other people or things.<br>3 It's hard to get interested in anything.

Copyright 1996, by Aaron T. Beck. All rights reserved.

ID #: ___________

Beck Depression Inventory, 2nd Edition (BDI-II)

Date: ___________

**13. Indecisiveness**<br>0 I make decisions about as well ever.<br>1 I find it more difficult to make decisions than usual.<br>2 I have much greater difficulty in making decisions than I used to.<br>3 I have trouble making any decisions.	**18. Changes in Appetite**<br>0 I have not experienced any change in my appetite.<br>1a My appetite is somewhat less than usual.<br>1b My appetite is somewhat greater than usual.<br>2a My appetite is much less than before.<br>2b My appetite is much greater than usual.<br>3a I have no appetite at all.<br>3b I crave food all the time.
**14. Worthlessness**<br>0 I do not feel I am worthless.<br>1 I don't consider myself as worthwhile and useful as I used to.<br>2 I feel more worthless as compared to other people.<br>3 I feel utterly worthless.	**19. Concentration Difficulty**<br>0 I can concentrate as well as ever.<br>1 I can't concentrate as well as usual.<br>2 it's hard to keep my mind on anything for very long.<br>3 I find I can't concentrate on anything.
**15. Loss of Energy**<br>0 I have as much energy as ever.<br>1 I have less energy than I used to have.<br>2 I don't have enough energy to do very much.<br>3 I don't have enough energy to do anything.	**20. Tiredness or Fatigue**<br>0 I am no more tired or fatigued than usual.<br>1 I get more tired or fatigued more easily than usual.<br>2 I am too tired or fatigued to do a lot of the things I used to.<br>3 I am too tired or fatigued to do most of the things I used to do.
**16. Changes in Sleeping Pattern**<br>0 I have not experienced any change in my sleeping pattern.<br>1a I sleep somewhat more than usual.<br>1b I sleep somewhat less than usual.<br>2a I sleep a lot more than usual.<br>2b I sleep a lot less than usual.<br>3a I sleep most of the day.<br>3b I wake up 1-2 hours early and can't get back to sleep.	**21. Loss of Interest in Sex**<br>0 I have not noticed any recent change in my interest in sex.<br>1 I am less interested in sex than I used to be.<br>2 I am much less interested in sex now.<br>3 I have lost interest in sex completely.
**17. Irritability**<br>0 I am no more irritable than usual.<br>1 I am more irritable than usual.<br>2 I am much more irritable than usual.<br>3 I am irritable all the time.	**Total Score:** ______

Copyright 1996, by Aaron T. Beck. All rights reserved.

14.4 Fatigue Severity Scale (FSS)

Participant ID: ______________
Date: ______________

Physical Therapy Toolbox: Fatigue Severity Scale

The Fatigue Severity Scale (FSS); is a method of evaluating the impact of fatigue on you. Please read each of the nine statements below and circle a number from 1 to 7, based on how closely you agree that the statement describes your condition during the past week.

A low value (1) indicates strong disagreement with the statement, whereas a high value (7) indicates strong agreement.

It is important that you circle a number (1 to 7); for every question.

During the past week, I have found that:	Disagree						Agree
1. My motivation is lower when I am fatigued.	1	2	3	4	5	6	7
2. Exercise brings on my fatigue.	1	2	3	4	5	6	7
3. I am easily fatigued.	1	2	3	4	5	6	7
4. Fatigue interferes with my physical functioning.	1	2	3	4	5	6	7
5. Fatigue causes frequent problems for me.	1	2	3	4	5	6	7
6. My fatigue prevents sustained physical functioning.	1	2	3	4	5	6	7
7. Fatigue interferes with carrying out certain duties and responsibilities.	1	2	3	4	5	6	7
8. Fatigue is among my three most disabling symptoms.	1	2	3	4	5	6	7
9. Fatigue interferes with my work, family, or social life.	1	2	3	4	5	6	7

14.5 Parkinson's Anxiety Scale (PAS)

Page 1 of 3 ID #:___________

Date:___________

The Parkinson Anxiety Scale (PAS): English Version

A. Persistent Anxiety

Please mark one circle for each item below

In the past four weeks, to what extent did you experience the following symptoms?

A.1. Feeling anxious or nervous

- Not at all, or never
- Very mild, or rarely
- Mild, or sometimes
- Moderate, or often
- Severe, or (nearly) always

A.2. Feeling Tense or stressed

- Not at all, or never
- Very mild, or rarely
- Mild, or sometimes
- Moderate, or often
- Severe, or (nearly) always

A.3. Being unable to relax

- Not at all, or never
- Very mild, or rarely
- Mild, or sometimes
- Moderate, or often
- Severe, or (nearly) always

A.4. Excessive worrying about everyday matters

- Not at all, or never
- Very mild, or rarely
- Mild, or sometimes
- Moderate, or often
- Severe, or (nearly) always

A.5. Fear of something bad, or even the worst, happening

- Not at all, or never
- Very mild, or rarely
- Mild, or sometimes
- Moderate, or often
- Severe, or (nearly) always

Page 2 of 3 ID #:___________

Date:___________

B. Episodic Anxiety

Please mark one circle for each item below

In the past four weeks, did you experience episodes of the following symptoms?

B.1. Panic or intense fear

- Never
- Rarely
- Sometimes
- Often
- Nearly Always

B.2. Shortness of breath

- Never
- Rarely
- Sometimes
- Often
- Nearly Always

B.3. Heart palpitations or heart beating fast (not related to physical effort or activity)

- Never
- Rarely
- Sometimes
- Often
- Nearly Always

B.4. Fear of losing control

- Never
- Rarely
- Sometimes
- Often
- Nearly Always

Page 3 of 3 ID #:___________

Date:___________

C. Avoidance Behavior

Please mark one circle for each item below

In the past four weeks, to what extent did you fear or avoid the following situations?

C.1. Social situations (where one may be observed, or evaluated by others, such as speaking in public, or talking to unknown people)

- Never
- Rarely
- Sometimes
- Often
- Nearly Always

C.2. Public setting (situations from which it may be difficult or embarrassing to escape, such as queues or lines, crowds, bridges, or public transportation)

- Never
- Rarely
- Sometimes
- Often
- Nearly Always

C.3. Specific objects or situations (such as flying, heights, spiders or other animals, needles, or blood)

- Never
- Rarely
- Sometimes
- Often
- Nearly Always

Copyright of this scale and it translations is held by the authors (Leentjens AFG, Dujardin K, Pontone GM, Starkstein SE, Weintraub D, and Martinez-Martin P; 2014). The scale and its translations are in the public domain and may be used without additional permission and free of charge on the condition that it's source is referenced.

Version 1 Leentjens et al, January 1, 2014

14.6 Starkstein Apathy Scale (SAS)

ID: ____________ Date: ___________

Apathy Scale

Please read each statement carefully. For each statement, please tick the column that best describes how you have been feeling in the last two weeks.

	Not at all	Slightly	Some	A lot
1. Are you interested in learning new things?				
2. Does anything interest you?				
3. Are you concerned about your condition?				
4. Do you put much effort into things?				
5. Are you always looking for something to do?				
6. Do you have plans and goals for the future?				
7. Do you have motivation?				
8. Do you have the energy for daily activities?				
9. Does someone have to tell you what to do each day?				
10. Are you indifferent to things?				
11. Are you unconcerned with many things?				
12. Do you need a push to get started on things?				
13. Are you neither happy nor sad, just in between?				
14. Would you consider yourself apathetic?				

Starkstein *et al.* 1992 V1 January 1, 1992

14.7 Parkinson's Disease Questionnaire - 39 (PDQ-39)

ID #: ____________
Date: ____________

Parkinson's Disease Quality of Life Questionnaire (PDQ-39)

Due to having Parkinson's disease,
how often <u>during the last month</u> have you…

Please check one box for each question

		Never	Occasionally	Sometimes	Often	Always or cannot do at all
1.	Had difficulty doing the leisure activities which you would like to do?	☐	☐	☐	☐	☐
2.	Had difficulty looking after your home, e.g. repairs/ improvements, housework, cooking?	☐	☐	☐	☐	☐
3.	Had difficulty carrying shopping (or grocery) bags?	☐	☐	☐	☐	☐
4.	Had problems walking 1 km (half a mile)?	☐	☐	☐	☐	☐
5.	Had problems walking 100 m (100 yards)?	☐	☐	☐	☐	☐
6.	Had problems getting around the house as easily as you would like?	☐	☐	☐	☐	☐
7.	Had difficulty getting around in public places?	☐	☐	☐	☐	☐
8.	Needed someone else to accompany you when you went out?	☐	☐	☐	☐	☐

Please make sure that you have ***checked one box for each question***, before going on to the next page.

Final English (Canada) PDQ-39.
PDQ-39 © Copyright, Oxford University Innovation Limited 1993. All Rights Reserved.
The authors, being Professor Crispin Jenkinson, Professor Ray Fitzpatrick and Ms Viv Peto, have asserted their moral rights.

ID #: ____________
Date: ____________

Due to having Parkinson's disease,
how often <u>during the last month</u> have you…

Please check one box for each question

		Never	Occasionally	Sometimes	Often	Always
9.	Felt frightened or worried about falling in public?	☐	☐	☐	☐	☐
10.	Been confined to the house more than you would like?	☐	☐	☐	☐	☐
11.	Had difficulty washing yourself?	☐	☐	☐	☐	☐
12.	Had difficulty dressing yourself?	☐	☐	☐	☐	☐
13.	Had problems doing up buttons or shoe laces?	☐	☐	☐	☐	☐
14.	Had problems writing clearly?	☐	☐	☐	☐	☐
15.	Had difficulty cutting up your food?	☐	☐	☐	☐	☐
16.	Had difficulty holding a drink without spilling it?	☐	☐	☐	☐	☐
17.	Felt depressed?	☐	☐	☐	☐	☐

Please make sure that you have ***checked one box for each question***, before going on to the next page.

ID #: ____________
Date: ____________

Due to having Parkinson's disease,
how often <u>during the last month</u> have you…

Please check one box for each question

		Never	Occasionally	Sometimes	Often	Always
18.	Felt isolated and lonely?	☐	☐	☐	☐	☐
19.	Felt weepy or tearful?	☐	☐	☐	☐	☐
20.	Felt angry or bitter?	☐	☐	☐	☐	☐
21.	Felt anxious?	☐	☐	☐	☐	☐
22.	Felt worried about your future?	☐	☐	☐	☐	☐
23.	Felt you had to conceal your Parkinson's from people?	☐	☐	☐	☐	☐
24.	Avoided situations which involve eating or drinking in public?	☐	☐	☐	☐	☐
25.	Felt embarrassed in public due to having Parkinson's disease?	☐	☐	☐	☐	☐
26.	Felt worried by other people's reaction to you?	☐	☐	☐	☐	☐
27.	Had problems with your close personal relationships?	☐	☐	☐	☐	☐

Please make sure that you have ***checked one box for each question***, before going on to the next page.

Final English (Canada) PDQ-39.
PDQ-39 © Copyright, Oxford University Innovation Limited 1993. All Rights Reserved.
The authors, being Professor Crispin Jenkinson, Professor Ray Fitzpatrick and Ms Viv Peto, have asserted their moral rights.

ID #: ____________
Date: ____________

Due to having Parkinson's disease,
how often <u>during the last month</u> have you…

Please check one box for each question

		Never	Occasionally	Sometimes	Often	Always
28.	Lacked support in the ways you need from your spouse or partner? *If you do not have a spouse or partner, please tick here* ☐	☐	☐	☐	☐	☐
29.	Lacked support in the ways you need from your family or close friends?	☐	☐	☐	☐	☐
30.	Unexpectedly fallen asleep during the day?	☐	☐	☐	☐	☐
31.	Had problems with your concentration, e.g. when reading or watching TV?	☐	☐	☐	☐	☐
32.	Felt your memory was bad?	☐	☐	☐	☐	☐
33.	Had distressing dreams or hallucinations?	☐	☐	☐	☐	☐
34.	Had difficulty with your speech?	☐	☐	☐	☐	☐
35.	Felt unable to communicate with people properly?	☐	☐	☐	☐	☐
36.	Felt ignored by people?	☐	☐	☐	☐	☐

Please make sure that you have ***checked one box for each question***, before going on to the next page.

Final English (Canada) PDQ-39.
PDQ-39 © Copyright, Oxford University Innovation Limited 1993. All Rights Reserved.
The authors, being Professor Crispin Jenkinson, Professor Ray Fitzpatrick and Ms Viv Peto, have asserted their moral rights.

ID #: ____________
Date: ____________

Due to having Parkinson's disease,
how often during the last month have you…

Please check one box for each question

	Never	Occasionally	Sometimes	Often	Always
**37.** Had painful muscle cramps or spasms?	☐	☐	☐	☐	☐
**38.** Had aches and pains in your joints or other parts of your body?	☐	☐	☐	☐	☐
**39.** Felt unpleasantly hot or cold?	☐	☐	☐	☐	☐

Please check that you have ***ticked one box for each question***.

Thank you for completing the questionnaire.

Final English (Canada) PDQ-39.
PDQ-39 © Copyright, Oxford University Innovation Limited 1993. All Rights Reserved.
The authors, being Professor Crispin Jenkinson, Professor Ray Fitzpatrick and Ms Viv Peto, have asserted their moral rights.

14.8 ROME III Constipation Module (ROME III)

TAP (H18-03083) | PI & Sponsor: Dr. Appel-Cresswell

ID #: ___________
Date: ___________

ROME III BOWEL DISORDERS MODULE

Question	Answer
41. In the last 3 months, how often did you have discomfort or pain anywhere in your abdomen?	⓪ Never → *Skip to question 52*<br>① Less than one day a month<br>② One day a month<br>③ Two to three days a month<br>④ One day a week<br>⑤ More than one day a week<br>⑥ Every day
42. Did you have pain only (not discomfort or a mixture of discomfort and pain)?	⓪ Never or rarely<br>① Sometimes<br>② Often<br>③ Most of the time<br>④ Always

Copyright 2000 © by Rome Foundation. All Rights Reserved.

ID #: ___________
Date: ___________

ROME III BOWEL DISORDERS MODULE

Question	Answer
43. For women: Did this discomfort or pain occur only during your menstrual bleeding and not at other times?	⓪ No<br>① Yes<br>② Does not apply because I have had the change in life (menopause) or I am a male
44. When you had this pain, how often did it limit or restrict your daily activities (for example, work, household activities, and social events)?	⓪ Never or rarely<br>① Sometimes<br>② Often<br>③ Most of the time<br>④ Always
45. Have you had this discomfort or pain 6 months or longer?	⓪ No<br>① Yes
46. How often did this discomfort or pain get better or stop after you had a bowel movement?	⓪ Never or rarely<br>① Sometimes<br>② Often<br>③ Most of the time<br>④ Always
47. When this discomfort or pain started, did you have more frequent bowel movements?	⓪ Never or rarely<br>① Sometimes<br>② Often<br>③ Most of the time<br>④ Always
48. When this discomfort or pain started, did you have less frequent bowel movements?	⓪ Never or rarely<br>① Sometimes<br>② Often<br>③ Most of the time<br>④ Always

Copyright 2000 © by Rome Foundation. All Rights Reserved.

ID #: ___________
Date: ___________

ROME III BOWEL DISORDERS MODULE

Symptoms in the Stomach and Intestines (continued)

Question	Answer
49. When this discomfort or pain started, were your stools (bowel movements) looser?	⓪ Never or rarely<br>① Sometimes<br>② Often<br>③ Most of the time<br>④ Always
50. When this discomfort or pain started, how often did you have harder stools?	⓪ Never or rarely<br>① Sometimes<br>② Often<br>③ Most of the time<br>④ Always
51. How often was this pain or discomfort relieved by moving or changing positions?	⓪ Never or rarely<br>① Sometimes<br>② Often<br>③ Most of the time<br>④ Always
52. In the last 3 months, how often did you have fewer than three bowel movements (0–2) a week?	⓪ Never or rarely<br>① Sometimes<br>② Often<br>③ Most of the time<br>④ Always
53. In the last 3 months, how often did you have hard or lumpy stools?*	⓪ Never or rarely<br>① Sometimes<br>② Often<br>③ Most of the time<br>④ Always

Copyright 2000 © by Rome Foundation. All Rights Reserved.

* *Those who wish to use the new criteria for subclassifying IBS patients into subtypes based on stool consistency may substitute the following response scale in Questions 53 and 61:*

⓪ Never or rarely
① About 25% of the time
② About 50% of the time
③ About 75% of the time
④ Always, 100% of the time

ID #: ___________
Date: ___________

ROME III BOWEL DISORDERS MODULE

Question	Answer
54. In the last 3 months, how often did you strain during bowel movements?	⓪ Never or rarely<br>① Sometimes<br>② Often<br>③ Most of the time<br>④ Always
55. In the last 3 months, how often did you have a feeling of incomplete emptying after bowel movements?	⓪ Never or rarely<br>① Sometimes<br>② Often<br>③ Most of the time<br>④ Always
56. In the last 3 months, how often did you have a sensation that the stool could not be passed, (i.e., was blocked), when having a bowel movement?	⓪ Never or rarely<br>① Sometimes<br>② Often<br>③ Most of the time<br>④ Always
57. In the last 3 months, how often did you press on or around your bottom or remove stool in order to complete a bowel movement?	⓪ Never or rarely<br>① Sometimes<br>② Often<br>③ Most of the time<br>④ Always
58. In the last 3 months, how often did you have difficulty relaxing or letting go to allow the stool to come out during a bowel movement?	⓪ Never or rarely<br>① Sometimes<br>② Often<br>③ Most of the time<br>④ Always
59. Did any of the symptoms of constipation listed in questions 52–58 above begin more than 6 months ago?	⓪ No<br>① Yes

Copyright 2000 © by Rome Foundation. All Rights Reserved.

ID #: ____________
Date:____________

ROME III BOWEL DISORDERS MODULE

Question	Answer	
**Symptoms in the Stomach and Intestines (continued)**		
60. In the last 3 months, how often did you have 4 or more bowel movements a day?	⓪ Never or rarely<br>① Sometimes<br>② Often<br>③ Most of the time<br>④ Always	
61. In the last 3 months, how often did you have loose, mushy, or watery stools?*	⓪ Never or rarely<br>① Sometimes<br>② Often<br>③ Most of the time<br>④ Always	→ *Skip to question 64* (after ⓪)
62. In the last 3 months, were at least three-fourths (3/4) of your stools loose, mushy, or watery?	⓪ No<br>① Yes	
63. Did you begin having frequent loose, mushy, or watery stools more than 6 months ago?	⓪ No<br>① Yes	
64. In the last 3 months, how often did you have to rush to the toilet to have a bowel movement?	⓪ Never or rarely<br>① Sometimes<br>② Often<br>③ Most of the time<br>④ Always	

Copyright 2000 © by Rome Foundation. All Rights Reserved.

** Those who wish to use the new criteria for subclassifying IBS patients into subtypes based on stool consistency may substitute the following response scale in Questions 53 and 61:*

⓪ Never or rarely
① About 25% of the time
② About 50% of the time
③ About 75% of the time
④ Always, 100% of the time

ID #: ____________
Date:____________

ROME III BOWEL DISORDERS MODULE

Question	Answer	
65. In the last 3 months, how often was there mucus or slime in your bowel movement?	⓪ Never or rarely<br>① Sometimes<br>② Often<br>③ Most of the time<br>④ Always	
66. In the last 3 months, how often did you have bloating or distension?	⓪ Never<br>① Less than one day a month<br>② One day a month<br>③ Two to three days a month<br>④ One day a week<br>⑤ More than one day a week<br>⑥ Every day	→ *Skip the question below* (after ⓪)
67. Did your symptoms of bloating or distention begin more than 6 months ago?	⓪ No<br>① Yes	

Copyright 2000 © by Rome Foundation. All Rights Reserved.

Additional Question (NOT a part of ROME III Bowel Module)

How would you rate your constipation in the last 2 weeks?

0 None: I have not experienced constipation
1 Mild: constipated some of the time
2 Moderate: constipated a lot of the time
3 Severe: constipated most or all of the time

14.9 The Physical Activity Scale for Individuals with Physical Disabilities (PASIPD)

ID#: ______________

Date: ______________

The Physical Activity Scale for Individuals with Physical Disabilities (PASIPD)

Leisure Time Activity

1. **During the past 7 days how often did you engage in stationary activities such as reading, watching TV, computer games, or doing handcrafts?**

1. Never (Go to question #2)	
2. Seldom (1-2d)	
3. Sometimes (3-4d)	
4. Often(5-7d)	

What were these activities? ______________________________

On average, how many hours per day did you spend in these stationary activities?

1. Less than 1 hour	
2. 1 but less than 2 hours	
3. 2-4h	
4. More than 4 hour	

2. **During the past 7 days, how often did you walk, wheel, push outside your home other than specifically for exercise. For example, getting to work or class, walking the dog, shopping, or other errands?**

1. Never (Go to question #3)	
2. Seldom (1-2d)	
3. Sometimes (3-4d)	
4. Often(5-7d)	

On average, how many hours per day did you spend wheeling or pushing outside your home?

1. Less than 1 hour	
2. 1 but less than 2 hours	
3. 2-4h	
4. More than 4 hour	

3. **During the past 7 days, how often did you engage in light sport or recreational activities such as bowling, golf with a cart, hunting or fishing, darts, billiards or pool, therapeutic exercise (physical or occupational therapy, stretching, use of a standing frame) or other similar activities?**

1. Never (Go to question #4)	
2. Seldom (1-2d)	
3. Sometimes (3-4d)	
4. Often(5-7d)	

Version 1 Washburn et al., February 1, 2002 Page 1 of 6

ID#: ______________

Date: ______________

What were these activities? ______________________________

On average, how many hour per day did you spend in these light sport or recreational activities?

1. Less than 1 hour	
2. 1 but less than 2 hours	
3. 2-4h	
4. More than 4 hour	

4. **During the past 7 days, how often did you engage in moderate sport and recreational activities such as doubles tennis, softball, golf without a cart, ballroom dancing, wheeling or pushing for pleasure or other similar activities?**

1. Never (Go to question #5)	
2. Seldom (1-2d)	
3. Sometimes (3-4d)	
4. Often(5-7d)	

What were these activities? ______________________________

On average, how many hours per day did you spend in these moderate sport and recreational activities?

1. Less than 1 hour	
2. 1 but less than 2 hours	
3. 2-4h	
4. More than 4 hour	

5. **During the past 7 days, how often did you engage in strenuous sport and recreational activities such as jogging, wheelchair racing (training), off-road pushing, swimming, aerobic dance, arm cranking, cycling (hand or leg), singles tennis, rugby, basketball, walking with crutches and braces, or other similar activities?**

1. Never (Go to question #6)	
2. Seldom (1-2d)	
3. Sometimes (3-4d)	
4. Often(5-7d)	

What were these activities? ______________________________

On average, how many hours per day did you spend in these strenuous sport or recreational activities?

1. Less than 1 hour	
2. 1 but less than 2 hours	
3. 2-4h	
4. More than 4 hour	

Version 1 Washburn et al., February 1, 2002 Page 2 of 6

ID#: ______________

Date: ______________

6. **During the past 7 days, how often did you do any exercise specifically to increase muscle strength and endurance such as lifting weights, push-ups, pull-ups, dips, or wheelchair push-ups, etc?**

1. Never (Go to question #7)	
2. Seldom (1-2d)	
3. Sometimes (3-4d)	
4. Often(5-7d)	

What were these activities? ______________________________

On average, how many hours per day did you spend in these exercises to increase muscle strength and endurance?

1. Less than 1 hour	
2. 1 but less than 2 hours	
3. 2-4h	
4. More than 4 hour	

Household Activity

7. **During the past 7 days, how often have you done any light housework, such as dusting, sweeping floors or washing dishes?**

1. Never (Go to question #8)	
2. Seldom (1-2d)	
3. Sometimes (3-4d)	
4. Often(5-7d)	

On average, how many hours per day did you spend doing light housework?

1. Less than 1 hour	
2. 1 but less than 2 hours	
3. 2-4h	
4. More than 4 hour	

8. **During the past 7 days, how often have you done any heavy housework or chores such as vacuuming, scrubbing floors, washing windows, or walls, etc?**

1. Never (Go to question #9)	
2. Seldom (1-2d)	
3. Sometimes (3-4d)	
4. Often(5-7d)	

Version 1 Washburn et al., February 1, 2002 Page 3 of 6

ID#: ______________

Date: ______________

On average, how many hours per day did you spend doing heavy housework or chores?

1. Less than 1 hour	
2. 1 but less than 2 hours	
3. 2-4h	
4. More than 4 hour	

9. **During the past 7 days, how often you done home repairs like carpentry, painting, furniture refinishing, electrical work, etc?**

1. Never (Go to question #10)	
2. Seldom (1-2d)	
3. Sometimes (3-4d)	
4. Often(5-7d)	

On average, how many hours per day did you spend doing home repairs?

1. Less than 1 hour	
2. 1 but less than 2 hours	
3. 2-4h	
4. More than 4 hour	

10. **During the past 7 days how often have you done lawn work or yard care including mowing, leaf or snow removal, tree or bush trimming, or wood chopping, etc?**

1. Never (Go to question #11)	
2. Seldom (1-2d)	
3. Sometimes (3-4d)	
4. Often(5-7d)	

On average, how many hours per day did you spend doing lawn work?

1. Less than 1 hour	
2. 1 but less than 2 hours	
3. 2-4h	
4. More than 4 hour	

11. **During the past 7 days, how often have you done outdoor gardening?**

1. Never (Go to question #12)	
2. Seldom (1-2d)	
3. Sometimes (3-4d)	
4. Often(5-7d)	

Version 1 Washburn et al., February 1, 2002 Page 4 of 6

ID#: ______________

Date: ______________

On average, how many hours per day did you spend doing outdoor gardening?

1. Less than 1 hour	
2. 1 but less than 2 hours	
3. 2-4h	
4. More than 4 hour	

12. During the past 7 days, how often did you care for another person, such as children, a dependent spouse, or another adult?

1. Never (Go to question #13)	
2. Seldom (1-2d)	
3. Sometimes (3-4d)	
4. Often(5-7d)	

On average, how many hours per day did you spend caring for another person?

1. Less than 1 hour	
2. 1 but less than 2 hours	
3. 2-4h	
4. More than 4 hour	

<u>Work-Related Activity</u>

13. During the past 7 days, how often did you work for pay or as a volunteer? (Exclude work that mainly involved sitting with slight arm movement such as light office work, computer work, light assembly line work, driving bus or van, etc.)

1. Never (Go to END)	
2. Seldom (1-2d)	
3. Sometimes (3-4d)	
4. Often(5-7d)	

On average, how many hours per day did you spend working for pay or as a volunteer?

1. Less than 1 hour	
2. 1 but less than 2 hours	
3. 2-4h	
4. More than 4 hour	

See scoring on next page

ID#: ______________

Date: ______________

<u>Scoring:</u>

sum of item multiplier x average hours per day over items 2-13

Item multipliers
1. Not scored
2. 2.5
3. 3.0
4. 4.0
5. 8.0
6. 5.5
7. 1.5
8. 4.0
9. 4.0
10. 4.0
11 4.0
12. 1.5
13. 2.5

SCORE: ____________

<u>Reference:</u>

Washburn, Richard A., et al. "The Physical Activity Scale for Individuals with Physical Disabilities: Development and Evaluation." *Archives of Physical Medicine and Rehabilitation*, vol. 83, no. 2, 2002, pp. 193–200., doi:10.1053/apmr.2002.27467. Accessed 12 Mar. 2018.

Diet History Questionnaire II

Adapted for Canada from the National Institutes of Health
Diet History Questionnaire II

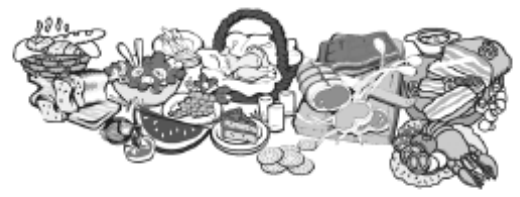

GENERAL INSTRUCTIONS

- **Answer each question as best you can. Estimate if you are not sure. A guess is better than leaving a blank.**
- **Use only a black ball-point pen. Do not use a pencil or felt-tip pen. Do not fold, staple, or tear the pages.**
- **Shade the box next to your answer, like this:** ■
- **If you make any changes, cross out the incorrect answer and shade in the box next to the correct answer. Also draw a circle around the correct answer.**
- **If you mark NEVER, NO, or DON'T KNOW for a question, please follow any arrows or instructions that direct you to the next question.**
- **Questions on portion size use measures like cups, ounces, teaspoons and tablespoons. Metric conversions are provided below.**

Volume	Weight
1 cup = 8 ounces = 250ml	1 ounce = 30g
1 fluid ounce = 30ml	
1 teaspoon = 5ml	
1 tablespoon = 15ml	

BEFORE TURNING THE PAGE, PLEASE COMPLETE THE FOLLOWING QUESTIONS.

Today's date: __ __ / __ __ / 2 0 __ __
month day year

In what month were you born?
☐ Jan ☐ Jul
☐ Feb ☐ Aug
☐ Mar ☐ Sep
☐ Apr ☐ Oct
☐ May ☐ Nov
☐ Jun ☐ Dec

In what year were you born? 1 9 __ __

Are you male or female?
☐ Male
☐ Female

BAR CODE LABEL OR SUBJECT ID HERE

DHQ II Past Month 28295

1. Over the past month, how often did you drink **tomato juice**?
   - ☐ NEVER (GO TO QUESTION 2)
   - ☐ 1 time in past month ☐ 2-3 times in past month ☐ 1-2 times per week ☐ 3-4 times per week ☐ 5-6 times per week
   - ☐ 1 time per day ☐ 2-3 times per day ☐ 4-5 times per day ☐ 6 or more times per day

   1a. Each time you drank **tomato juice**, how much did you usually drink?
   - ☐ Less than ½ cup (4 ounces)
   - ☐ ½ to 1¼ cups (4 to 10 ounces)
   - ☐ More than 1¼ cups (10 ounces)

2. Over the past month, how often did you drink **other vegetable juice**? *(Please do not include tomato juice.)*
   - ☐ NEVER (GO TO QUESTION 3)
   - ☐ 1 time in past month ☐ 2-3 times in past month ☐ 1-2 times per week ☐ 3-4 times per week ☐ 5-6 times per week
   - ☐ 1 time per day ☐ 2-3 times per day ☐ 4-5 times per day ☐ 6 or more times per day

   2a. Each time you drank **other vegetable juice**, how much did you usually drink?
   - ☐ Less than ¾ cup (6 ounces)
   - ☐ ¾ to 1¼ cups (6 to 10 ounces)
   - ☐ More than 1¼ cups (10 ounces)

3. Over the past month, how often did you drink 100% **orange juice** or **grapefruit juice**?
   - ☐ NEVER (GO TO QUESTION 4)
   - ☐ 1 time in past month ☐ 2-3 times in past month ☐ 1-2 times per week ☐ 3-4 times per week ☐ 5-6 times per week
   - ☐ 1 time per day ☐ 2-3 times per day ☐ 4-5 times per day ☐ 6 or more times per day

   3a. Each time you drank 100% **orange juice** or **grapefruit juice**, how much did you usually drink?
   - ☐ Less than ¾ cup (6 ounces)
   - ☐ ¾ to 1¼ cups (6 to 10 ounces)
   - ☐ More than 1¼ cups (10 ounces)

Question 4 appears in the next column

   3b. How often was the orange juice or grapefruit juice you drank **calcium-fortified**?
   - ☐ Almost never or never
   - ☐ About ¼ of the time
   - ☐ About ½ of the time
   - ☐ About ¾ of the time
   - ☐ Almost always or always

4. Over the past month, how often did you drink **other 100% fruit juice** or **100% fruit juice mixtures** (such as apple, grape, pineapple, or others)?
   - ☐ NEVER (GO TO QUESTION 5)
   - ☐ 1 time in past month ☐ 2-3 times in past month ☐ 1-2 times per week ☐ 3-4 times per week ☐ 5-6 times per week
   - ☐ 1 time per day ☐ 2-3 times per day ☐ 4-5 times per day ☐ 6 or more times per day

   4a. Each time you drank **other 100% fruit juice** or **100% fruit juice mixtures**, how much did you usually drink?
   - ☐ Less than ¾ cup (6 ounces)
   - ☐ ¾ to 1½ cups (6 to 12 ounces)
   - ☐ More than 1½ cups (12 ounces)

5. How often did you drink **other fruit drinks** (such as Fruité, Fruitopia, Five Alive, Sunny D, or Kool-Aid, diet or regular)?
   - ☐ NEVER (GO TO QUESTION 6)
   - ☐ 1 time in past month ☐ 2-3 times in past month ☐ 1-2 times per week ☐ 3-4 times per week ☐ 5-6 times per week
   - ☐ 1 time per day ☐ 2-3 times per day ☐ 4-5 times per day ☐ 6 or more times per day

   5a. Each time you drank **other fruit drinks**, how much did you usually drink?
   - ☐ Less than 1 cup (8 ounces)
   - ☐ 1 to 2 cups (8 to 16 ounces)
   - ☐ More than 2 cups (16 ounces)

   5b. How often were your other fruit drinks **diet** or **low calorie**?
   - ☐ Almost never or never
   - ☐ About ¼ of the time
   - ☐ About ½ of the time
   - ☐ About ¾ of the time
   - ☐ Almost always or always

Question 6 appears on the next page

2 28295

Over the past month...

6. How often did you drink **milk** or **milk substitutes as a beverage** (NOT in coffee, NOT in cereal)? *(Please do not include chocolate milk, hot chocolate, and milk in milkshakes or meal replacement beverages.)*
   - ☐ NEVER (GO TO QUESTION 7)
   - ☐ 1 time in past month ☐ 2-3 times in past month ☐ 1-2 times per week ☐ 3-4 times per week ☐ 5-6 times per week
   - ☐ 1 time per day ☐ 2-3 times per day ☐ 4-5 times per day ☐ 6 or more times per day

   6a. Each time you drank **milk** or **milk substitutes as a beverage**, how much did you usually drink?
   - ☐ Less than 1 cup (8 ounces)
   - ☐ 1 to 1½ cups (8 to 12 ounces)
   - ☐ More than 1½ cups (12 ounces)

   6b. What kind of **milk** or **milk substitutes** did you usually drink?
   - ☐ Whole milk
   - ☐ 2% fat milk
   - ☐ 1% fat milk
   - ☐ Skim, nonfat, or 0.5% fat milk
   - ☐ Soy milk
   - ☐ Rice milk
   - ☐ Almond milk
   - ☐ Other

7. How often did you drink **chocolate milk** or **hot chocolate**?
   - ☐ NEVER (GO TO QUESTION 8)
   - ☐ 1 time in past month ☐ 2-3 times in past month ☐ 1-2 times per week ☐ 3-4 times per week ☐ 5-6 times per week
   - ☐ 1 time per day ☐ 2-3 times per day ☐ 4-5 times per day ☐ 6 or more times per day

   7a. Each time you drank **chocolate milk** or **hot chocolate**, how much did you usually drink?
   - ☐ Less than 1 cup (8 ounces)
   - ☐ 1 to 2 cups (8 to 16 ounces)
   - ☐ More than 2 cups (16 ounces)

   7b. How often was the chocolate milk or hot chocolate you drank **reduced-fat** or **fat-free**?
   - ☐ Almost never or never
   - ☐ About ¼ of the time
   - ☐ About ½ of the time
   - ☐ About ¾ of the time
   - ☐ Almost always or always

Question 8 appears in the next column

8. How often did you drink **milkshakes**?
   - ☐ NEVER (GO TO QUESTION 9)
   - ☐ 1 time in past month ☐ 2-3 times in past month ☐ 1-2 times per week ☐ 3-4 times per week ☐ 5-6 times per week
   - ☐ 1 time per day ☐ 2-3 times per day ☐ 4-5 times per day ☐ 6 or more times per day

   8a. Each time you drank **milkshakes**, how much did you usually drink?
   - ☐ Less than 1 cup (8 ounces)
   - ☐ 1 to 2 cups (8 to 16 ounces)
   - ☐ More than 2 cups (16 ounces)

9. How often did you drink **meal replacement** or **high-protein beverages** (such as Boost, Breakfast Essential, Ensure, Slimfast or others)?
   - ☐ NEVER (GO TO QUESTION 10)
   - ☐ 1 time in past month ☐ 2-3 times in past month ☐ 1-2 times per week ☐ 3-4 times per week ☐ 5-6 times per week
   - ☐ 1 time per day ☐ 2-3 times per day ☐ 4-5 times per day ☐ 6 or more times per day

   9a. Each time you drank **meal replacement** or **high-protein beverages**, how much did you usually drink?
   - ☐ Less than 1 cup (8 ounces)
   - ☐ 1 to 1½ cups (8 to 12 ounces)
   - ☐ More than 1½ cups (12 ounces)

10. How often did you drink **soft drinks** or **pop?**
   - ☐ NEVER (GO TO QUESTION 11)
   - ☐ 1 time in past month ☐ 2-3 times in past month ☐ 1-2 times per week ☐ 3-4 times per week ☐ 5-6 times per week
   - ☐ 1 time per day ☐ 2-3 times per day ☐ 4-5 times per day ☐ 6 or more times per day

   10a. Each time you drank **soft drinks** or **pop**, how much did you usually drink?
   - ☐ Less than 12 ounces or less than 1 regular size can or bottle (355 ml)
   - ☐ 12 to 16 ounces or 1 regular size can or bottle (355 ml)
   - ☐ More than 16 ounces or more than 1 regular size can or bottle (355 ml)

Question 11 appears on the next page

3 28295

Over the past month...

   10b. How often were your soft drinks or pop **diet** or **calorie-free**?
   - ☐ Almost never or never
   - ☐ About ¼ of the time
   - ☐ About ½ of the time
   - ☐ About ¾ of the time
   - ☐ Almost always or always

   10c. How often were your soft drinks or pop **caffeine-free**?
   - ☐ Almost never or never
   - ☐ About ¼ of the time
   - ☐ About ½ of the time
   - ☐ About ¾ of the time
   - ☐ Almost always or always

11. How often did you drink **sports drinks** (such as PowerAde or Gatorade)?
   - ☐ NEVER (GO TO QUESTION 12)
   - ☐ 1 time in past month ☐ 2-3 times in past month ☐ 1-2 times per week ☐ 3-4 times per week ☐ 5-6 times per week
   - ☐ 1 time per day ☐ 2-3 times per day ☐ 4-5 times per day ☐ 6 or more times per day

   11a. Each time you drank **sports drinks**, how much did you usually drink?
   - ☐ Less than 1½ cups (12 ounces)
   - ☐ 1½ to 3 cups (12 to 24 ounces)
   - ☐ More than 3 cups (24 ounces)

12. How often did you drink **energy drinks** (such as Red Bull, Rock Star, Full Throttle, or Monster)?
   - ☐ NEVER (GO TO QUESTION 13)
   - ☐ 1 time in past month ☐ 2-3 times in past month ☐ 1-2 times per week ☐ 3-4 times per week ☐ 5-6 times per week
   - ☐ 1 time per day ☐ 2-3 times per day ☐ 4-5 times per day ☐ 6 or more times per day

   12a. Each time you drank **energy drinks**, how much did you usually drink?
   - ☐ Less than 1 cup (8 ounces)
   - ☐ 1 to 2 cups (8 to 16 ounces)
   - ☐ More than 2 cups (16 ounces)

Question 13 appears in the next column

13. How often did you drink **beer**?
   - ☐ NEVER (GO TO QUESTION 14)
   - ☐ 1 time in past month ☐ 2-3 times in past month ☐ 1-2 times per week ☐ 3-4 times per week ☐ 5-6 times per week
   - ☐ 1 time per day ☐ 2-3 times per day ☐ 4-5 times per day ☐ 6 or more times per day

   13a. Each time you drank **beer**, how much did you usually drink?
   - ☐ Less than 1 regular size can or bottle (341 ml)
   - ☐ 1 to 3 regular size cans or bottles
   - ☐ More than 3 regular size cans or bottles

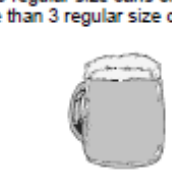

14. How often did you drink **water** (including tap, bottled, carbonated, flavoured, or vitamin added water)?
   - ☐ NEVER (GO TO QUESTION 15)
   - ☐ 1 time in past month ☐ 2-3 times in past month ☐ 1-2 times per week ☐ 3-4 times per week ☐ 5-6 times per week
   - ☐ 1 time per day ☐ 2-3 times per day ☐ 4-5 times per day ☐ 6 or more times per day

   14a. Each time you drank **water**, how much did you usually drink?
   - ☐ Less than 1 cup (8 ounces)
   - ☐ 1 to 4 cups (8 to 32 ounces)
   - ☐ More than 4 cups (32 ounces)

   14b. How often was the water you drank **tap water**?
   - ☐ Almost never or never
   - ☐ About ¼ of the time
   - ☐ About ½ of the time
   - ☐ About ¾ of the time
   - ☐ Almost always or always

   14c. How often was the water you drank **bottled, sweetened water** (with low or no-calorie sweetener, including carbonated water)?
   - ☐ Almost never or never
   - ☐ About ¼ of the time
   - ☐ About ½ of the time
   - ☐ About ¾ of the time
   - ☐ Almost always or always

Question 15 appears on the next page

4 28295

Over the past month...

14d. How often was the bottled sweetened water you drank **with added vitamins or minerals** (such as Aquafina Plus, Vitaminwater, or others)?

- ☐ Almost never or never
- ☐ About ¼ of the time
- ☐ About ½ of the time
- ☐ About ¾ of the time
- ☐ Almost always or always

15. How often did you drink **wine**?

- ☐ NEVER (GO TO QUESTION 16)
- ☐ 1 time in past month
- ☐ 2-3 times in past month
- ☐ 1-2 times per week
- ☐ 3-4 times per week
- ☐ 5-6 times per week
- ☐ 1 time per day
- ☐ 2-3 times per day
- ☐ 4-5 times per day
- ☐ 6 or more times per day

15a. Each time you drank **wine**, how much did you usually drink?

- ☐ Less than 1 glass (5 ounces)
- ☐ 1 to 2 glasses (5 to 12 ounces)
- ☐ More than 2 glasses (12 ounces)

15b. How often was the wine you drank **red wine**?

- ☐ Almost never or never
- ☐ About ¼ of the time
- ☐ About ½ of the time
- ☐ About ¾ of the time
- ☐ Almost always or always

16. How often did you drink **liquor** or **mixed drinks**?

- ☐ NEVER (GO TO QUESTION 17)
- ☐ 1 time in past month
- ☐ 2-3 times in past month
- ☐ 1-2 times per week
- ☐ 3-4 times per week
- ☐ 5-6 times per week
- ☐ 1 time per day
- ☐ 2-3 times per day
- ☐ 4-5 times per day
- ☐ 6 or more times per day

16a. Each time you drank **liquor** or **mixed drinks**, how much did you usually drink?

- ☐ Less than 1 shot of liquor
- ☐ 1 to 3 shots of liquor
- ☐ More than 3 shots of liquor

Question 17 appears in the next column

17. How often did you eat **oatmeal, Cream of Wheat, Red River,** or **other cooked cereal**?

- ☐ NEVER (GO TO QUESTION 18)
- ☐ 1 time in past month
- ☐ 2-3 times in past month
- ☐ 1 time per week
- ☐ 2 times per week
- ☐ 3-4 times per week
- ☐ 5-6 times per week
- ☐ 1 time per day
- ☐ 2 or more times per day

17a. Each time you ate **oatmeal, Cream of Wheat, Red River,** or **other cooked cereal**, how much did you usually eat?

- ☐ Less than ¾ cup
- ☐ ¾ to 1¼ cups
- ☐ More than 1¼ cups

17b. How often was **butter** or **margarine** added to your oatmeal, Cream of Wheat, Red River, or other cooked cereal?

- ☐ Almost never or never
- ☐ About ¼ of the time
- ☐ About ½ of the time
- ☐ About ¾ of the time
- ☐ Almost always or always

17c. Was **milk** added to your oatmeal, Cream of Wheat, Red River, or other cooked cereal?

- ☐ NO (GO TO QUESTION 18)
- ☐ YES

17d. What kind of **milk** was usually added?

- ☐ Whole milk
- ☐ 2% fat milk
- ☐ 1% fat milk
- ☐ Skim, nonfat, or 0.5% fat milk
- ☐ Soy milk
- ☐ Rice milk
- ☐ Almond milk
- ☐ Other

17e. Each time **milk** was added to your oatmeal, Cream of Wheat, Red River, or other cooked cereal, how much was usually added?

- ☐ Less than ⅓ cup
- ☐ ⅓ to 1 cup
- ☐ More than 1 cup

Question 18 appears on the next page

Over the past month...

18. How often did you eat **cold cereal**?

- ☐ NEVER (GO TO QUESTION 19)
- ☐ 1 time in past month
- ☐ 2-3 times in past month
- ☐ 1 time per week
- ☐ 2 times per week
- ☐ 3-4 times per week
- ☐ 5-6 times per week
- ☐ 1 time per day
- ☐ 2 or more times per day

18a. Each time you ate **cold cereal**, how much did you usually eat?

- ☐ Less than 1 cup
- ☐ 1 to 2½ cups
- ☐ More than 2½ cups

18b. How often was the cold cereal you ate **Vector** or **PC Force Active**?

- ☐ Almost never or never
- ☐ About ¼ of the time
- ☐ About ½ of the time
- ☐ About ¾ of the time
- ☐ Almost always or always

18c. How often was the cold cereal you ate **All Bran, Fibre 1, Fibre First, 100% Bran,** or **All-Bran Buds**?

- ☐ Almost never or never
- ☐ About ¼ of the time
- ☐ About ½ of the time
- ☐ About ¾ of the time
- ☐ Almost always or always

18d. How often was the cold cereal you ate **some other bran** or **fibre cereal** (such as Cheerios (regular), Shredded Wheat, Raisin Bran, Bran Flakes, Mini-Wheats, Shreddies, Honey Bunches of Oats, Oatmeal Crisp or others)?

- ☐ Almost never or never
- ☐ About ¼ of the time
- ☐ About ½ of the time
- ☐ About ¾ of the time
- ☐ Almost always or always

18e. How often was the cold cereal you ate any **other type of cold cereal** (such as Corn Flakes, Rice Krispies, Frosted Flakes, Special K, Froot Loops, Cap'n Crunch, Honey Nut Cheerios, Honeycomb, or others)?

- ☐ Almost never or never
- ☐ About ¼ of the time
- ☐ About ½ of the time
- ☐ About ¾ of the time
- ☐ Almost always or always

Question 19 appears in the next column

18f. Was **milk** added to your cold cereal?

- ☐ NO (GO TO QUESTION 19)
- ☐ YES

18g. What kind of **milk** was usually added?

- ☐ Whole milk
- ☐ 2% fat milk
- ☐ 1% fat milk
- ☐ Skim, nonfat, or 0.5% fat milk
- ☐ Soy milk
- ☐ Rice milk
- ☐ Almond milk
- ☐ Other

18h. Each time **milk** was added to your cold cereal, how much was usually added?

- ☐ Less than ½ cup
- ☐ ½ to 1 cup
- ☐ More than 1 cup

19. How often did you eat **applesauce**?

- ☐ NEVER (GO TO QUESTION 20)
- ☐ 1 time in past month
- ☐ 2-3 times in past month
- ☐ 1 time per week
- ☐ 2 times per week
- ☐ 3-4 times per week
- ☐ 5-6 times per week
- ☐ 1 time per day
- ☐ 2 or more times per day

19a. Each time you ate **applesauce**, how much did you usually eat?

- ☐ Less than ¼ cup
- ☐ ¼ to ¾ cup
- ☐ More than ¾ cup

20. How often did you eat **apples**?

- ☐ NEVER (GO TO QUESTION 21)
- ☐ 1 time in past month
- ☐ 2-3 times in past month
- ☐ 1 time per week
- ☐ 2 times per week
- ☐ 3-4 times per week
- ☐ 5-6 times per week
- ☐ 1 time per day
- ☐ 2 or more times per day

20a. Each time you ate **apples**, how many did you usually eat?

- ☐ Less than 1 apple
- ☐ 1 apple
- ☐ More than 1 apple

Question 21 appears on the next page

Over the past month...

21. How often did you eat **pears** (fresh, canned, or frozen)?

- ☐ NEVER (GO TO QUESTION 22)
- ☐ 1 time in past month
- ☐ 2-3 times in past month
- ☐ 1 time per week
- ☐ 2 times per week
- ☐ 3-4 times per week
- ☐ 5-6 times per week
- ☐ 1 time per day
- ☐ 2 or more times per day

21a. Each time you ate **pears**, how many did you usually eat?

- ☐ Less than 1 pear
- ☐ 1 pear
- ☐ More than 1 pear

22. How often did you eat **bananas**?

- ☐ NEVER (GO TO QUESTION 23)
- ☐ 1 time in past month
- ☐ 2-3 times in past month
- ☐ 1 time per week
- ☐ 2 times per week
- ☐ 3-4 times per week
- ☐ 5-6 times per week
- ☐ 1 time per day
- ☐ 2 or more times per day

22a. Each time you ate **bananas**, how many did you usually eat?

- ☐ Less than 1 banana
- ☐ 1 banana
- ☐ More than 1 banana

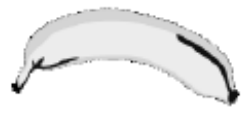

23. How often did you eat **dried fruit** (such as prunes or raisins)? *(Please do not include dried apricots.)*

- ☐ NEVER (GO TO QUESTION 24)
- ☐ 1 time in past month
- ☐ 2-3 times in past month
- ☐ 1 time per week
- ☐ 2 times per week
- ☐ 3-4 times per week
- ☐ 5-6 times per week
- ☐ 1 time per day
- ☐ 2 or more times per day

23a. Each time you ate **dried fruit**, how much did you usually eat?

- ☐ Less than 2 tablespoons
- ☐ 2 to 5 tablespoons
- ☐ More than 5 tablespoons

Question 24 appears in the next column

24. How often did you eat **peaches**, **nectarines**, or **plums** (fresh, canned or frozen)?

- ☐ NEVER (GO TO QUESTION 25)
- ☐ 1 time in past month
- ☐ 2-3 times in past month
- ☐ 1 time per week
- ☐ 2 times per week
- ☐ 3-4 times per week
- ☐ 5-6 times per week
- ☐ 1 time per day
- ☐ 2 or more times per day

24a. Each time you ate **peaches**, **nectarines**, or **plums**, how much did you usually eat?

- ☐ Less than 1 fruit or less than ½ cup
- ☐ 1 to 2 fruits or ½ to ¾ cup
- ☐ More than 2 fruits or more than ¾ cup

25. How often did you eat **grapes**?

- ☐ NEVER (GO TO QUESTION 26)
- ☐ 1 time in past month
- ☐ 2-3 times in past month
- ☐ 1 time per week
- ☐ 2 times per week
- ☐ 3-4 times per week
- ☐ 5-6 times per week
- ☐ 1 time per day
- ☐ 2 or more times per day

25a. Each time you ate **grapes**, how much did you usually eat?

- ☐ Less than ½ cup or less than 10 grapes
- ☐ ½ to 1 cup or 10 to 30 grapes
- ☐ More than 1 cup or more than 30 grapes

26. How often did you eat **cantaloupe**?

- ☐ NEVER (GO TO QUESTION 27)
- ☐ 1 time in past month
- ☐ 2-3 times in past month
- ☐ 1 time per week
- ☐ 2 times per week
- ☐ 3-4 times per week
- ☐ 5-6 times per week
- ☐ 1 time per day
- ☐ 2 or more times per day

26a. Each time you ate **cantaloupe**, how much did you usually eat?

- ☐ Less than ¼ melon or less than ½ cup
- ☐ ¼ melon or ½ to 1 cup
- ☐ More than ¼ melon or more than 1 cup

Question 27 appears on the next page

Over the past month...

27. How often did you eat **melon, other than cantaloupe** (such as watermelon or honeydew)?

- ☐ NEVER (GO TO QUESTION 28)
- ☐ 1 time in past month
- ☐ 2-3 times in past month
- ☐ 1 time per week
- ☐ 2 times per week
- ☐ 3-4 times per week
- ☐ 5-6 times per week
- ☐ 1 time per day
- ☐ 2 or more times per day

27a. Each time you ate **melon other than cantaloupe**, how much did you usually eat?

- ☐ Less than 1 cup or 2 small wedges
- ☐ 1 to 3 cups or 2 medium wedges
- ☐ More than 3 cups or 2 large wedges

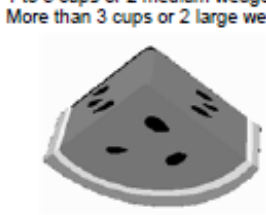

28. How often did you eat **strawberries** (fresh or frozen)?

- ☐ NEVER (GO TO QUESTION 29)
- ☐ 1 time in past month
- ☐ 2-3 times in past month
- ☐ 1 time per week
- ☐ 2 times per week
- ☐ 3-4 times per week
- ☐ 5-6 times per week
- ☐ 1 time per day
- ☐ 2 or more times per day

28a. Each time you ate **strawberries**, how much did you usually eat?

- ☐ Less than ¼ cup or less than 4 berries
- ☐ ¼ to ¾ cup or 4 to 10 berries
- ☐ More than ¾ cup or more than 10 berries

29. How often did you eat **blueberries, raspberries, saskatoon berries** or **blackberries** (fresh or frozen)?

- ☐ NEVER (GO TO QUESTION 30)
- ☐ 1 time in past month
- ☐ 2-3 times in past month
- ☐ 1 time per week
- ☐ 2 times per week
- ☐ 3-4 times per week
- ☐ 5-6 times per week
- ☐ 1 time per day
- ☐ 2 or more times per day

Question 30 appears in the next column

29a. Each time you ate **blueberries, raspberries, saskatoon berries** or **blackberries**, how much did you usually eat?

- ☐ Less than ¼ cup
- ☐ ¼ to ¾ cup
- ☐ More than ¾ cup

30. How often did you eat **oranges**, **tangelos**, **mandarins**, or **clementines**?

- ☐ NEVER (GO TO QUESTION 31)
- ☐ 1 time in past month
- ☐ 2-3 times in past month
- ☐ 1 time per week
- ☐ 2 times per week
- ☐ 3-4 times per week
- ☐ 5-6 times per week
- ☐ 1 time per day
- ☐ 2 or more times per day

30a. Each time you ate **oranges**, **tangelos**, **mandarins**, or **clementines**, how many did you usually eat?

- ☐ Less than 1 fruit
- ☐ 1 fruit
- ☐ More than 1 fruit

31. How often did you eat **grapefruit**?

- ☐ NEVER (GO TO QUESTION 32)
- ☐ 1 time in past month
- ☐ 2-3 times in past month
- ☐ 1 time per week
- ☐ 2 times per week
- ☐ 3-4 times per week
- ☐ 5-6 times per week
- ☐ 1 time per day
- ☐ 2 or more times per day

31a. Each time you ate **grapefruit**, how much did you usually eat?

- ☐ Less than ½ grapefruit
- ☐ ½ to 1 grapefruit
- ☐ More than 1 grapefruit

32. How often did you eat **pineapple**?

- ☐ NEVER (GO TO QUESTION 33)
- ☐ 1 time in past month
- ☐ 2-3 times in past month
- ☐ 1 time per week
- ☐ 2 times per week
- ☐ 3-4 times per week
- ☐ 5-6 times per week
- ☐ 1 time per day
- ☐ 2 or more times per day

32a. Each time you ate **pineapple**, how much did you usually eat?

- ☐ Less than ¼ cup or less than 1 medium slice
- ☐ ¼ to ¾ cup or 1 to 2 medium slices
- ☐ More than ¾ cup or more than 2 medium slices

Question 33 appears on the next page

Over the past month...

33. How often did you eat **other kinds of fruit**?

☐ NEVER (GO TO QUESTION 34)

☐ 1 time in past month
☐ 2-3 times in past month
☐ 1 time per week
☐ 2 times per week
☐ 3-4 times per week
☐ 5-6 times per week
☐ 1 time per day
☐ 2 or more times per day

33a. Each time you ate **other kinds of fruit**, how much did you usually eat?

☐ Less than ¼ cup
☐ ¼ to ¾ cup
☐ More than ¾ cup

34. How often did you eat **COOKED greens** (such as spinach, turnip greens, collard, mustard greens, chard, or kale)?

☐ NEVER (GO TO QUESTION 35)

☐ 1 time in past month
☐ 2-3 times in past month
☐ 1 time per week
☐ 2 times per week
☐ 3-4 times per week
☐ 5-6 times per week
☐ 1 time per day
☐ 2 or more times per day

34a. Each time you ate **COOKED greens**, how much did you usually eat?

☐ Less than ½ cup
☐ ½ to 1 cup
☐ More than 1 cup

35. How often did you eat **RAW greens** (such as spinach, chard, or kale)? *(We will ask about lettuce later.)*

☐ NEVER (GO TO QUESTION 36)

☐ 1 time in past month
☐ 2-3 times in past month
☐ 1 time per week
☐ 2 times per week
☐ 3-4 times per week
☐ 5-6 times per week
☐ 1 time per day
☐ 2 or more times per day

35a. Each time you ate **RAW greens**, how much did you usually eat?

☐ Less than 1½ cups
☐ 1½ to 3 cups
☐ More than 3 cups

Question 36 appears in the next column

36. How often did you eat **coleslaw**?

☐ NEVER (GO TO QUESTION 37)

☐ 1 time in past month
☐ 2-3 times in past month
☐ 1 time per week
☐ 2 times per week
☐ 3-4 times per week
☐ 5-6 times per week
☐ 1 time per day
☐ 2 or more times per day

36a. Each time you ate **coleslaw**, how much did you usually eat?

☐ Less than ¼ cup
☐ ¼ to ¾ cup
☐ More than ¾ cup

37. How often did you eat **sauerkraut** or **cabbage** (other than coleslaw)?

☐ NEVER (GO TO QUESTION 38)

☐ 1 time in past month
☐ 2-3 times in past month
☐ 1 time per week
☐ 2 times per week
☐ 3-4 times per week
☐ 5-6 times per week
☐ 1 time per day
☐ 2 or more times per day

37a. Each time you ate **sauerkraut** or **cabbage**, how much did you usually eat?

☐ Less than ¼ cup
☐ ¼ to 1 cup
☐ More than 1 cup

38. How often did you eat **carrots** (fresh, canned, or frozen)?

☐ NEVER (GO TO QUESTION 39)

☐ 1 time in past month
☐ 2-3 times in past month
☐ 1 time per week
☐ 2 times per week
☐ 3-4 times per week
☐ 5-6 times per week
☐ 1 time per day
☐ 2 or more times per day

38a. Each time you ate **carrots**, how much did you usually eat?

☐ Less than ¼ cup or less than 2 baby carrots
☐ ¼ to ¾ cup or 2 to 8 baby carrots
☐ More than ¾ cup or more than 8 baby carrots

Question 39 appears on the next page

Over the past month...

39. How often did you eat **string beans** or **green beans** (fresh, canned, or frozen)?

☐ NEVER (GO TO QUESTION 40)

☐ 1 time in past month
☐ 2-3 times in past month
☐ 1 time per week
☐ 2 times per week
☐ 3-4 times per week
☐ 5-6 times per week
☐ 1 time per day
☐ 2 or more times per day

39a. Each time you ate **string beans** or **green beans**, how much did you usually eat?

☐ Less than ½ cup
☐ ½ to 1 cup
☐ More than 1 cup

40. How often did you eat **peas** (fresh, canned, or frozen)?

☐ NEVER (GO TO QUESTION 41)

☐ 1 time in past month
☐ 2-3 times in past month
☐ 1 time per week
☐ 2 times per week
☐ 3-4 times per week
☐ 5-6 times per week
☐ 1 time per day
☐ 2 or more times per day

40a. Each time you ate **peas**, how much did you usually eat?

☐ Less than ¼ cup
☐ ¼ to ½ cup
☐ More than ½ cup

41. How often did you eat **corn** (fresh, canned or frozen)?

☐ NEVER (GO TO QUESTION 42)

☐ 1 time in past month
☐ 2-3 times in past month
☐ 1 time per week
☐ 2 times per week
☐ 3-4 times per week
☐ 5-6 times per week
☐ 1 time per day
☐ 2 or more times per day

41a. Each time you ate **corn**, how much did you usually eat?

☐ Less than 1 ear or less than ½ cup
☐ 1 ear or ½ to 1 cup
☐ More than 1 ear or more than 1 cup

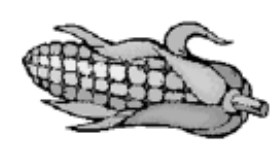

Question 42 appears in the next column

42. How often did you eat **broccoli** (fresh or frozen)?

☐ NEVER (GO TO QUESTION 43)

☐ 1 time in past month
☐ 2-3 times in past month
☐ 1 time per week
☐ 2 times per week
☐ 3-4 times per week
☐ 5-6 times per week
☐ 1 time per day
☐ 2 or more times per day

42a. Each time you ate **broccoli**, how much did you usually eat?

☐ Less than ¼ cup
☐ ¼ to 1 cup
☐ More than 1 cup

43. How often did you eat **cauliflower** (fresh or frozen)?

☐ NEVER (GO TO QUESTION 44)

☐ 1 time in past month
☐ 2-3 times in past month
☐ 1 time per week
☐ 2 times per week
☐ 3-4 times per week
☐ 5-6 times per week
☐ 1 time per day
☐ 2 or more times per day

43a. Each time you ate **cauliflower**, how much did you usually eat?

☐ Less than ¼ cup
☐ ¼ to 1 cup
☐ More than 1 cup

44. How often did you eat **Brussels sprouts** (fresh or frozen)?

☐ NEVER (GO TO QUESTION 45)

☐ 1 time in past month
☐ 2-3 times in past month
☐ 1 time per week
☐ 2 times per week
☐ 3-4 times per week
☐ 5-6 times per week
☐ 1 time per day
☐ 2 or more times per day

44a. Each time you ate **Brussels sprouts**, how much did you usually eat?

☐ Less than 4 Brussels sprouts or less than ½ cup
☐ 4 to 7 Brussels sprouts or ½ to 1 cup
☐ More than 7 Brussels sprouts or more than 1 cup

Question 45 appears on the next page

Over the past month...

45. How often did you eat **asparagus** (fresh or frozen)?

☐ NEVER (GO TO QUESTION 46)

☐ 1 time in past month
☐ 2-3 times in past month
☐ 1 time per week
☐ 2 times per week
☐ 3-4 times per week
☐ 5-6 times per week
☐ 1 time per day
☐ 2 or more times per day

45a. Each time you ate **asparagus**, how much did you usually eat?

☐ Less than 5 spears or less than ½ cup
☐ 5 to 9 spears or ½ to ¾ cup
☐ More than 9 spears or more than ¾ cup

46. How often did you eat **winter squash** (such as pumpkin, butternut, or acorn)?

☐ NEVER (GO TO QUESTION 47)

☐ 1 time in past month
☐ 2-3 times in past month
☐ 1 time per week
☐ 2 times per week
☐ 3-4 times per week
☐ 5-6 times per week
☐ 1 time per day
☐ 2 or more times per day

46a. Each time you ate **winter squash**, how much did you usually eat?

☐ Less than ⅓ cup
☐ ⅓ to 1 cup
☐ More than 1 cup

47. How often did you eat **mixed vegetables** (such as vegetable stir fry, frozen or canned mixed vegetables)?

☐ NEVER (GO TO QUESTION 48)

☐ 1 time in past month
☐ 2-3 times in past month
☐ 1 time per week
☐ 2 times per week
☐ 3-4 times per week
☐ 5-6 times per week
☐ 1 time per day
☐ 2 or more times per day

47a. Each time you ate **mixed vegetables**, how much did you usually eat?

☐ Less than ½ cup
☐ ½ to 1 cup
☐ More than 1 cup

Question 48 appears in the next column

48. How often did you eat **onions**?

☐ NEVER (GO TO QUESTION 49)

☐ 1 time in past month
☐ 2-3 times in past month
☐ 1 time per week
☐ 2 times per week
☐ 3-4 times per week
☐ 5-6 times per week
☐ 1 time per day
☐ 2 or more times per day

48a. Each time you ate **onions**, how much did you usually eat?

☐ Less than 1 slice or less than 1 tablespoon
☐ 1 to 5 slices or 1 to 4 tablespoons
☐ More than 5 slices or more than 4 tablespoons

49. Now think about all the **cooked vegetables** you ate in the past month and how they were prepared. How often were your vegetables **COOKED WITH** some sort of **fat**, including oil spray? *(Please do not include potatoes.)*

☐ NEVER (GO TO QUESTION 50)

☐ 1 time in past month
☐ 2-3 times in past month
☐ 1 time per week
☐ 2 times per week
☐ 3-4 times per week
☐ 5-6 times per week
☐ 1 time per day
☐ 2 or more times per day

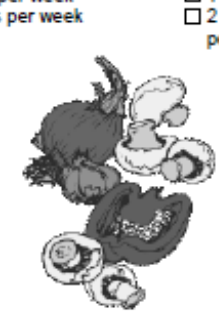

49a. Which fats were usually added to your vegetables **DURING COOKING**? *(Please do not include potatoes. Mark all that apply.)*

☐ Margarine (including light)
☐ Butter (including light)
☐ Lard, fatback, or bacon fat
☐ Olive oil
☐ Corn oil
☐ Canola or rapeseed oil
☐ Oil spray, such as Pam or others
☐ Other kinds of oil
☐ None of the above

Question 50 appears on the next page

Over the past month...

50. Now, thinking again about all the **cooked vegetables** you ate in the past month, how often was some sort of fat, sauce, or dressing added **AFTER COOKING OR AT THE TABLE**? *(Please do not include potatoes.)*

☐ NEVER (GO TO QUESTION 51)

☐ 1 time in past month
☐ 2-3 times in past month
☐ 1-2 times per week
☐ 3-4 times per week
☐ 5-6 times per week
☐ 1 time per day
☐ 2 times per day
☐ 3 or more times per day

50a. Which fats, sauces, or dressings were usually added **AFTER COOKING OR AT THE TABLE**? *(Please do not include potatoes. Mark all that apply.)*

☐ Margarine (including light)
☐ Butter (including light)
☐ Lard, fatback, or bacon fat
☐ Vegetable oil
☐ Salad dressing
☐ Cheese sauce
☐ White sauce
☐ Other

50b. If margarine, butter, vegetable oil, lard, fatback, or bacon fat was added to your cooked vegetables **AFTER COOKING OR AT THE TABLE**, how much did you usually add?

☐ Did not usually add these
☐ Less than 1 teaspoon
☐ 1 to 3 teaspoons
☐ More than 3 teaspoons

50c. If salad dressing, cheese sauce, or white sauce was added to your cooked vegetables **AFTER COOKING OR AT THE TABLE**, how much did you usually add?

☐ Did not usually add these
☐ Less than 1 tablespoon
☐ 1 to 3 tablespoons
☐ More than 3 tablespoons

51. How often did you eat **sweet peppers** (green, red, or yellow)?

☐ NEVER (GO TO QUESTION 52)

☐ 1 time in past month
☐ 2-3 times in past month
☐ 1 time per week
☐ 2 times per week
☐ 3-4 times per week
☐ 5-6 times per week
☐ 1 time per day
☐ 2 or more times per day

Question 52 appears in the next column

51a. Each time you ate **sweet peppers**, how much did you usually eat?

☐ Less than ⅛ pepper
☐ ⅛ to ½ pepper
☐ More than ½ pepper

52. How often did you eat **fresh tomatoes** (including those in salads)?

☐ NEVER (GO TO QUESTION 53)

☐ 1 time in past month
☐ 2-3 times in past month
☐ 1 time per week
☐ 2 times per week
☐ 3-4 times per week
☐ 5-6 times per week
☐ 1 time per day
☐ 2 or more times per day

52a. Each time you ate **fresh tomatoes**, how much did you usually eat?

☐ Less than ¼ tomato
☐ ¼ to 1 tomato
☐ More than 1 tomato

53. How often did you eat **lettuce salads** (with or without other vegetables)?

☐ NEVER (GO TO QUESTION 54)

☐ 1 time in past month
☐ 2-3 times in past month
☐ 1 time per week
☐ 2 times per week
☐ 3-4 times per week
☐ 5-6 times per week
☐ 1 time per day
☐ 2 or more times per day

53a. Each time you ate **lettuce salads**, how much did you usually eat?

☐ Less than ¼ cup
☐ ¼ to 1½ cups
☐ More than 1½ cups

53b. How often did the lettuce salads you ate include **dark green lettuce**?

☐ Almost never or never
☐ About ¼ of the time
☐ About ½ of the time
☐ About ¾ of the time
☐ Almost always or always

54. How often did you eat **salad dressing** (including low-fat) **on salads**?

☐ NEVER (GO TO QUESTION 55)

☐ 1 time in past month
☐ 2-3 times in past month
☐ 1 time per week
☐ 2 times per week
☐ 3-4 times per week
☐ 5-6 times per week
☐ 1 time per day
☐ 2 or more times per day

Question 55 appears on the next page

Over the <u>past month</u>...

54a. Each time you ate **salad dressing on salads**, how much did you usually eat?

- ☐ Less than 2 tablespoons
- ☐ 2 to 4 tablespoons
- ☐ More than 4 tablespoons

55. How often did you eat **sweet potatoes** or **yams**?

- ☐ NEVER (GO TO QUESTION 56)
- ☐ 1 time in past month
- ☐ 2-3 times in past month
- ☐ 1 time per week
- ☐ 2 times per week
- ☐ 3-4 times per week
- ☐ 5-6 times per week
- ☐ 1 time per day
- ☐ 2 or more times per day

55a. Each time you ate **sweet potatoes** or **yams**, how much did you usually eat?

- ☐ 1 small potato or less than ½ cup
- ☐ 1 medium potato or ½ to 1 cup
- ☐ 1 large potato or more than 1 cup

56. How often did you eat **French fries**, **home fries**, **hash browned potatoes**, or **tater tots**?

- ☐ NEVER (GO TO QUESTION 57)
- ☐ 1 time in past month
- ☐ 2-3 times in past month
- ☐ 1 time per week
- ☐ 2 times per week
- ☐ 3-4 times per week
- ☐ 5-6 times per week
- ☐ 1 time per day
- ☐ 2 or more times per day

56a. Each time you ate **French fries**, **home fries**, **hash browned potatoes**, or **tater tots** how much did you usually eat?

- ☐ Less than 10 fries or less than ½ cup
- ☐ 10 to 25 fries or ½ to 1 cup
- ☐ More than 25 fries or more than 1 cup

56b. How often did the French fries, home fries, hash browned potatoes, or tater tots you ate include **poutine** (**with gravy and cheese**)?

- ☐ Almost never or never
- ☐ About ¼ of the time
- ☐ About ½ of the time
- ☐ About ¾ of the time
- ☐ Almost always or always

Question 57 appears in the next column

57. How often did you eat **potato salad**?

- ☐ NEVER (GO TO QUESTION 58)
- ☐ 1 time in past month
- ☐ 2-3 times in past month
- ☐ 1 time per week
- ☐ 2 times per week
- ☐ 3-4 times per week
- ☐ 5-6 times per week
- ☐ 1 time per day
- ☐ 2 or more times per day

57a. Each time you ate **potato salad**, how much did you usually eat?

- ☐ Less than ½ cup
- ☐ ½ to 1 cup
- ☐ More than 1 cup

58. How often did you eat **baked**, **boiled**, or **mashed potatoes**?

- ☐ NEVER (GO TO QUESTION 59)
- ☐ 1 time in past month
- ☐ 2-3 times in past month
- ☐ 1 time per week
- ☐ 2 times per week
- ☐ 3-4 times per week
- ☐ 5-6 times per week
- ☐ 1 time per day
- ☐ 2 or more times per day

58a. Each time you ate **baked**, **boiled**, or **mashed potatoes**, how much did you usually eat?

- ☐ 1 small potato or less than ½ cup
- ☐ 1 medium potato or ½ to 1 cup
- ☐ 1 large potato or more than 1 cup

58b. How often was **sour cream** (including low-fat) added to your potatoes, **EITHER IN COOKING OR AT THE TABLE**?

- ☐ Almost never or never (GO TO QUESTION 58d)
- ☐ About ¼ of the time
- ☐ About ½ of the time
- ☐ About ¾ of the time
- ☐ Almost always or always

58c. Each time **sour cream** was added to your potatoes, how much was usually added?

- ☐ Less than 1 tablespoon
- ☐ 1 to 4 tablespoons
- ☐ More than 4 tablespoons

58d. How often was **margarine** (including light) added to your potatoes, **EITHER IN COOKING OR AT THE TABLE**?

- ☐ Almost never or never
- ☐ About ¼ of the time
- ☐ About ½ of the time
- ☐ About ¾ of the time
- ☐ Almost always or always

Question 59 appears on the next page

Over the <u>past month</u>...

58e. How often was **butter** (including light) added to your potatoes, **EITHER IN COOKING OR AT THE TABLE**?

- ☐ Almost never or never
- ☐ About ¼ of the time
- ☐ About ½ of the time
- ☐ About ¾ of the time
- ☐ Almost always or always

58f. Each time **margarine** or **butter** was added to your potatoes, how much was usually added?

- ☐ Never added
- ☐ Less than 1 teaspoon
- ☐ 1 to 3 teaspoons
- ☐ More than 3 teaspoons

58g. How often was **cheese** or **cheese sauce** added to your potatoes, **EITHER IN COOKING OR AT THE TABLE**?

- ☐ Almost never or never (GO TO QUESTION 59)
- ☐ About ¼ of the time
- ☐ About ½ of the time
- ☐ About ¾ of the time
- ☐ Almost always or always

58h. Each time **cheese** or **cheese sauce** was added to your potatoes, how much was usually added?

- ☐ Less than 1 tablespoon
- ☐ 1 to 3 tablespoons
- ☐ More than 3 tablespoons

59. How often did you eat **salsa**?

- ☐ NEVER (GO TO QUESTION 60)
- ☐ 1 time in past month
- ☐ 2-3 times in past month
- ☐ 1 time per week
- ☐ 2 times per week
- ☐ 3-4 times per week
- ☐ 5-6 times per week
- ☐ 1 time per day
- ☐ 2 or more times per day

59a. Each time you ate **salsa**, how much did you usually eat?

- ☐ Less than 2 tablespoons
- ☐ 2 tablespoons to ½ cup
- ☐ More than ½ cup

Question 60 appears in the next column

60. How often did you eat **ketchup**?

- ☐ NEVER (GO TO QUESTION 61)
- ☐ 1 time in past month
- ☐ 2-3 times in past month
- ☐ 1 time per week
- ☐ 2 times per week
- ☐ 3-4 times per week
- ☐ 5-6 times per week
- ☐ 1 time per day
- ☐ 2 or more times per day

60a. Each time you ate **ketchup**, how much did you usually eat?

- ☐ Less than 2 teaspoons
- ☐ 2 to 6 teaspoons
- ☐ More than 6 teaspoons

61. How often did you eat **stuffing**, **dressing**, or **dumplings**?

- ☐ NEVER (GO TO QUESTION 62)
- ☐ 1 time in past month
- ☐ 2-3 times in past month
- ☐ 1 time per week
- ☐ 2 times per week
- ☐ 3-4 times per week
- ☐ 5-6 times per week
- ☐ 1 time per day
- ☐ 2 or more times per day

61a. Each time you ate **stuffing**, **dressing**, or **dumplings**, how much did you usually eat?

- ☐ Less than ½ cup
- ☐ ½ to 1 cup
- ☐ More than 1 cup

62. How often did you eat **chili**?

- ☐ NEVER (GO TO QUESTION 63)
- ☐ 1 time in past month
- ☐ 2-3 times in past month
- ☐ 1 time per week
- ☐ 2 times per week
- ☐ 3-4 times per week
- ☐ 5-6 times per week
- ☐ 1 time per day
- ☐ 2 or more times per day

62a. Each time you ate **chili**, how much did you usually eat?

- ☐ Less than 1 cup
- ☐ 1 to 1¾ cups
- ☐ More than 1¾ cups

Question 63 appears on the next page

Over the <u>past month</u>...

63. How often did you eat **Mexican foods** (such as tacos, tostados, burritos, tamales, fajitas, enchiladas, quesadillas, or chimichangas)?

- ☐ NEVER (GO TO QUESTION 64)
- ☐ 1 time in past month
- ☐ 2-3 times in past month
- ☐ 1 time per week
- ☐ 2 times per week
- ☐ 3-4 times per week
- ☐ 5-6 times per week
- ☐ 1 time per day
- ☐ 2 or more times per day

63a. Each time you ate **Mexican foods**, how much did you usually eat?

- ☐ Less than 1 taco, burrito, etc.
- ☐ 1 to 2 tacos, burritos, etc.
- ☐ More than 2 tacos, burritos, etc.

64. How often did you eat **baked beans**? *(Please include canned, ready-made, or homemade.)*

- ☐ NEVER (GO TO QUESTION 65)
- ☐ 1 time in past month
- ☐ 2-3 times in past month
- ☐ 1 time per week
- ☐ 2 times per week
- ☐ 3-4 times per week
- ☐ 5-6 times per week
- ☐ 1 time per day
- ☐ 2 or more times per day

64a. Each time you ate **baked beans**, how much did you usually eat?

- ☐ Less than ½ cup
- ☐ ½ to 1 cup
- ☐ More than 1 cup

65. How often did you eat **other cooked dried beans** (such as pintos, kidney, black-eyed peas, lima, lentils, soybeans, or refried beans)? *(Please do not include bean, pea or lentil soups, or chili.)*

- ☐ NEVER (GO TO QUESTION 66)
- ☐ 1 time in past month
- ☐ 2-3 times in past month
- ☐ 1 time per week
- ☐ 2 times per week
- ☐ 3-4 times per week
- ☐ 5-6 times per week
- ☐ 1 time per day
- ☐ 2 or more times per day

65a. Each time you ate **other beans**, how much did you usually eat?

- ☐ Less than ¼ cup
- ☐ ¼ to ¾ cup
- ☐ More than ¾ cup

Question 66 appears in the next column

65b. How often were the other beans you ate **refried beans**, **beans prepared with any type of fat**, or **with meat added**?

- ☐ Almost never or never
- ☐ About ¼ of the time
- ☐ About ½ of the time
- ☐ About ¾ of the time
- ☐ Almost always or always

66. How often did you eat **other kinds of vegetables**?

- ☐ NEVER (GO TO QUESTION 67)
- ☐ 1 time in past month
- ☐ 2-3 times in past month
- ☐ 1 time per week
- ☐ 2 times per week
- ☐ 3-4 times per week
- ☐ 5-6 times per week
- ☐ 1 time per day
- ☐ 2 or more times per day

66a. Each time you ate **other kinds of vegetables**, how much did you usually eat?

- ☐ Less than ¼ cup
- ☐ ¼ to ½ cup
- ☐ More than ½ cup

67. How often did you eat **rice** or **other cooked grains** (such as bulgur, cracked wheat, or millet)?

- ☐ NEVER (GO TO QUESTION 68)
- ☐ 1 time in past month
- ☐ 2-3 times in past month
- ☐ 1 time per week
- ☐ 2 times per week
- ☐ 3-4 times per week
- ☐ 5-6 times per week
- ☐ 1 time per day
- ☐ 2 or more times per day

67a. Each time you ate **rice** or **other cooked grains**, how much did you usually eat?

- ☐ Less than ½ cup
- ☐ ½ to 1½ cups
- ☐ More than 1½ cups

67b. How often was **butter**, **margarine**, or **oil** added to your rice or other cooked grains **IN COOKING OR AT THE TABLE**?

- ☐ Almost never or never
- ☐ About ¼ of the time
- ☐ About ½ of the time
- ☐ About ¾ of the time
- ☐ Almost always or always

Question 68 appears on the next page

Over the <u>past month</u>...

68. How often did you eat **pancakes**, **waffles**, or **French toast**?

- ☐ NEVER (GO TO QUESTION 69)
- ☐ 1 time in past month
- ☐ 2-3 times in past month
- ☐ 1 time per week
- ☐ 2 times per week
- ☐ 3-4 times per week
- ☐ 5-6 times per week
- ☐ 1 time per day
- ☐ 2 or more times per day

68a. Each time you ate **pancakes**, **waffles**, or **French toast**, how much did you usually eat?

- ☐ Less than 1 medium piece
- ☐ 1 to 3 medium pieces
- ☐ More than 3 medium pieces

68b. How often was **margarine** (including light) added to your pancakes, waffles, or French toast **AFTER COOKING OR AT THE TABLE**?

- ☐ Almost never or never
- ☐ About ¼ of the time
- ☐ About ½ of the time
- ☐ About ¾ of the time
- ☐ Almost always or always

68c. How often was **butter** (including light) added to your pancakes, waffles, or French toast **AFTER COOKING OR AT THE TABLE**?

- ☐ Almost never or never
- ☐ About ¼ of the time
- ☐ About ½ of the time
- ☐ About ¾ of the time
- ☐ Almost always or always

68d. Each time **margarine** or **butter** was added to your pancakes, waffles, or French toast, how much was usually added?

- ☐ Never added
- ☐ Less than 1 teaspoon
- ☐ 1 to 3 teaspoons
- ☐ More than 3 teaspoons

68e. How often was **syrup** added to your pancakes, waffles, or French toast?

- ☐ Almost never or never (GO TO QUESTION 69)
- ☐ About ¼ of the time
- ☐ About ½ of the time
- ☐ About ¾ of the time
- ☐ Almost always or always

68f. Each time **syrup** was added to your pancakes, waffles, or French toast, how much was usually added?

- ☐ Less than 1 tablespoon
- ☐ 1 to 3 tablespoons
- ☐ More than 3 tablespoons

Question 69 appears in the next column

69. How often did you eat **lasagna**, **stuffed shells**, **stuffed manicotti**, **ravioli**, or **tortellini**? *(Please do not include spaghetti or other pasta.)*

- ☐ NEVER (GO TO QUESTION 70)
- ☐ 1 time in past month
- ☐ 2-3 times in past month
- ☐ 1 time per week
- ☐ 2 times per week
- ☐ 3-4 times per week
- ☐ 5-6 times per week
- ☐ 1 time per day
- ☐ 2 or more times per day

69a. Each time you ate **lasagna**, **stuffed shells**, **stuffed manicotti**, **ravioli**, or **tortellini**, how much did you usually eat?

- ☐ Less than 1 cup
- ☐ 1 to 2 cups
- ☐ More than 2 cups

70. How often did you eat **macaroni and cheese**?

- ☐ NEVER (GO TO QUESTION 71)
- ☐ 1 time in past month
- ☐ 2-3 times in past month
- ☐ 1 time per week
- ☐ 2 times per week
- ☐ 3-4 times per week
- ☐ 5-6 times per week
- ☐ 1 time per day
- ☐ 2 or more times per day

70a. Each time you ate **macaroni and cheese**, how much did you usually eat?

- ☐ Less than 1 cup
- ☐ 1 to 1½ cups
- ☐ More than 1½ cups

71. How often did you eat **pasta salad** or **macaroni salad**?

- ☐ NEVER (GO TO QUESTION 72)
- ☐ 1 time in past month
- ☐ 2-3 times in past month
- ☐ 1 time per week
- ☐ 2 times per week
- ☐ 3-4 times per week
- ☐ 5-6 times per week
- ☐ 1 time per day
- ☐ 2 or more times per day

71a. Each time you ate **pasta salad** or **macaroni salad**, how much did you usually eat?

- ☐ Less than ½ cup
- ☐ ½ to 1 cup
- ☐ More than 1 cup

Question 72 appears on the next page

Over the <u>past month</u>...

72. Other than the pastas listed in Questions 69, 70, and 71, how often did you eat **pasta**, **spaghetti**, or **other noodles**?

☐ NEVER (GO TO QUESTION 73)

☐ 1 time in past month
☐ 2-3 times in past month
☐ 1 time per week
☐ 2 times per week
☐ 3-4 times per week
☐ 5-6 times per week
☐ 1 time per day
☐ 2 or more times per day

72a. Each time you ate **pasta**, **spaghetti**, or **other noodles**, how much did you usually eat?

☐ Less than 1 cup
☐ 1 to 3 cups
☐ More than 3 cups

72b. How often did you eat your pasta, spaghetti, or other noodles with **tomato sauce** or **spaghetti sauce made WITH meat**?

☐ Almost never or never
☐ About ¼ of the time
☐ About ½ of the time
☐ About ¾ of the time
☐ Almost always or always

72c. How often did you eat your pasta, spaghetti, or other noodles with **tomato sauce** or **spaghetti sauce made WITHOUT meat**?

☐ Almost never or never
☐ About ¼ of the time
☐ About ½ of the time
☐ About ¾ of the time
☐ Almost always or always

72d. How often did you eat your pasta, spaghetti, or other noodles with **margarine**, **butter**, **oil**, or **cream sauce**?

☐ Almost never or never
☐ About ¼ of the time
☐ About ½ of the time
☐ About ¾ of the time
☐ Almost always or always

Question 73 appears in the next column

73. How often did you eat **bagels** or **English muffins**?

☐ NEVER (GO TO INTRODUCTION TO QUESTION 74)

☐ 1 time in past month
☐ 2-3 times in past month
☐ 1 time per week
☐ 2 times per week
☐ 3-4 times per week
☐ 5-6 times per week
☐ 1 time per day
☐ 2 or more times per day

73a. How often were the bagels or English muffins you ate **whole wheat**?

☐ Almost never or never
☐ About ¼ of the time
☐ About ½ of the time
☐ About ¾ of the time
☐ Almost always or always

73b. Each time you ate **bagels** or **English muffins**, how many did you usually eat?

☐ Less than 1 bagel or English muffin
☐ 1 bagel or English muffin
☐ More than 1 bagel or English muffin

73c. How often was **margarine** (including light) added to your bagels or English muffins?

☐ Almost never or never
☐ About ¼ of the time
☐ About ½ of the time
☐ About ¾ of the time
☐ Almost always or always

73d. How often was **butter** (including light) added to your bagels or English muffins?

☐ Almost never or never
☐ About ¼ of the time
☐ About ½ of the time
☐ About ¾ of the time
☐ Almost always or always

73e. Each time **margarine** or **butter** was added to your bagels or English muffins, how much was usually added?

☐ Never added
☐ Less than 1 teaspoon
☐ 1 to 2 teaspoons
☐ More than 2 teaspoons

Introduction to Question 74 appears on the next page

Over the <u>past month</u>...

73f. How often was **cream cheese** (including low-fat) spread on your bagels or English muffins?

☐ Almost never or never (GO TO INTRODUCTION TO QUESTION 74)
☐ About ¼ of the time
☐ About ½ of the time
☐ About ¾ of the time
☐ Almost always or always

73g. Each time **cream cheese** was added to your bagels or English muffins, how much was usually added?

☐ Less than 1 tablespoon
☐ 1 to 2 tablespoons
☐ More than 2 tablespoons

The next questions ask about your intake of breads other than bagels or English muffins. First, we will ask about bread you ate as part of sandwiches only. Then we will ask about all other bread you ate.

74. How often did you eat **breads, rolls** or **flatbreads** (such as pita, roti and tortillas) **AS PART OF SANDWICHES** (including burger and hot dog rolls)?

☐ NEVER (GO TO QUESTION 75)

☐ 1 time in past month
☐ 2-3 times in past month
☐ 1 time per week
☐ 2 times per week
☐ 3-4 times per week
☐ 5-6 times per week
☐ 1 time per day
☐ 2 or more times per day

74a. Each time you ate **breads, rolls** or **flatbreads AS PART OF SANDWICHES**, how many did you usually eat?

☐ 1 slice or ½ roll or flatbread
☐ 2 slices or 1 roll or flatbread
☐ More than 2 slices or more than 1 roll or flatbread

74b. How often were the breads, rolls or flatbreads that you used for your sandwiches **white** (including burger and hot dog rolls)?

☐ Almost never or never
☐ About ¼ of the time
☐ About ½ of the time
☐ About ¾ of the time
☐ Almost always or always

Question 75 appears in the next column

74c. How often was **mayonnaise** or **mayonnaise-type dressing** (including low-fat) added to the breads, rolls or flatbreads used for your sandwiches or wraps?

☐ Almost never or never (GO TO QUESTION 74e)
☐ About ¼ of the time
☐ About ½ of the time
☐ About ¾ of the time
☐ Almost always or always

74d. Each time **mayonnaise** or **mayonnaise-type dressing** was added to the breads, rolls or flatbreads used for your sandwiches or wraps, how much was usually added?

☐ Less than 1 teaspoon
☐ 1 to 3 teaspoons
☐ More than 3 teaspoons

74e. How often was **margarine** (including light) added to the breads, rolls or flatbreads used for your sandwiches or wraps?

☐ Almost never or never
☐ About ¼ of the time
☐ About ½ of the time
☐ About ¾ of the time
☐ Almost always or always

74f. How often was **butter** (including low-fat) added to the breads, rolls or flatbreads used for your sandwiches or wraps?

☐ Almost never or never
☐ About ¼ of the time
☐ About ½ of the time
☐ About ¾ of the time
☐ Almost always or always

74g. Each time **margarine** or **butter** was added to the breads, rolls or flatbreads used for your sandwiches or wraps, how much was usually added?

☐ Never added
☐ Less than 1 teaspoon
☐ 1 to 2 teaspoons
☐ More than 2 teaspoons

75. How often did you eat **breads, dinner rolls** or **flatbreads, NOT AS PART OF SANDWICHES**?

☐ NEVER (GO TO QUESTION 76)

☐ 1 time in past month
☐ 2-3 times in past month
☐ 1 time per week
☐ 2 times per week
☐ 3-4 times per week
☐ 5-6 times per week
☐ 1 time per day
☐ 2 or more times per day

Question 76 appears on the next page

Over the <u>past month</u>...

75a. Each time you ate **breads, dinner rolls** or **flatbreads, NOT AS PART OF SANDWICHES**, how much did you usually eat?

☐ 1 slice or 1 dinner roll or ½ flatbread
☐ 2 slices or 2 dinner rolls or 1 flatbread
☐ More than 2 slices or dinner rolls or more than 1 flatbread

75b. How often were the breads, rolls or flatbreads you ate **white**?

☐ Almost never or never
☐ About ¼ of the time
☐ About ½ of the time
☐ About ¾ of the time
☐ Almost always or always

75c. How often was **margarine** (including light) added to your breads, rolls or flatbreads?

☐ Almost never or never
☐ About ¼ of the time
☐ About ½ of the time
☐ About ¾ of the time
☐ Almost always or always

75d. How often was **butter** (including light) added to your breads, rolls or flatbreads?

☐ Almost never or never
☐ About ¼ of the time
☐ About ½ of the time
☐ About ¾ of the time
☐ Almost always or always

75e. Each time **margarine** or **butter** was added to your breads, rolls or flatbreads, how much was usually added?

☐ Never added
☐ Less than 1 teaspoon
☐ 1 to 2 teaspoons
☐ More than 2 teaspoons

75f. How often was **cream cheese** (including low-fat) added to your breads, rolls or flatbreads?

☐ Almost never or never (GO TO QUESTION 76)
☐ About ¼ of the time
☐ About ½ of the time
☐ About ¾ of the time
☐ Almost always or always

Question 76 appears in the next column

75g. Each time **cream cheese** was added to your breads, rolls or flatbreads, how much was usually added?

☐ Less than 1 tablespoon
☐ 1 to 2 tablespoons
☐ More than 2 tablespoons

76. How often did you eat **jam**, **jelly**, or **honey** on bagels, muffins, bread, rolls, or crackers?

☐ NEVER (GO TO QUESTION 77)

☐ 1 time in past month
☐ 2-3 times in past month
☐ 1 time per week
☐ 2 times per week
☐ 3-4 times per week
☐ 5-6 times per week
☐ 1 time per day
☐ 2 or more times per day

76a. Each time you ate **jam**, **jelly**, or **honey**, how much did you usually eat?

☐ Less than 2 teaspoons
☐ 2 to 4 teaspoons
☐ More than 4 teaspoons

77. How often did you eat **peanut butter** or **other nut butter**?

☐ NEVER (GO TO QUESTION 78)

☐ 1 time in past month
☐ 2-3 times in past month
☐ 1 time per week
☐ 2 times per week
☐ 3-4 times per week
☐ 5-6 times per week
☐ 1 time per day
☐ 2 or more times per day

77a. Each time you ate **peanut butter** or **other nut butter**, how much did you usually eat?

☐ Less than 1 tablespoon
☐ 1 to 2 tablespoons
☐ More than 2 tablespoons

78. How often did you eat **roast beef** or **steak IN SANDWICHES**?

☐ NEVER (GO TO QUESTION 79)

☐ 1 time in past month
☐ 2-3 times in past month
☐ 1 time per week
☐ 2 times per week
☐ 3-4 times per week
☐ 5-6 times per week
☐ 1 time per day
☐ 2 or more times per day

78a. Each time you ate **roast beef** or **steak IN SANDWICHES**, how much did you usually eat?

☐ Less than 1 slice or less than 2 ounces
☐ 1 to 2 slices or 2 to 4 ounces
☐ More than 2 slices or more than 4 ounces

Question 79 appears on the next page

Over the <u>past month</u>...

79. How often did you eat **turkey** or **chicken COLD CUTS** (such as loaf, luncheon meat, turkey ham, turkey salami, or turkey pastrami)? *(We will ask about other turkey or chicken later.)*

☐ NEVER (GO TO QUESTION 80)

☐ 1 time in past month
☐ 2-3 times in past month
☐ 1 time per week
☐ 2 times per week
☐ 3-4 times per week
☐ 5-6 times per week
☐ 1 time per day
☐ 2 or more times per day

79a. Each time you ate **turkey** or **chicken COLD CUTS**, how much did you usually eat?

☐ Less than 1 slice
☐ 1 to 3 slices
☐ More than 3 slices

80. How often did you eat **luncheon** or **deli-style ham**? *(We will ask about other ham later.)*

☐ NEVER (GO TO QUESTION 81)

☐ 1 time in past month
☐ 2-3 times in past month
☐ 1 time per week
☐ 2 times per week
☐ 3-4 times per week
☐ 5-6 times per week
☐ 1 time per day
☐ 2 or more times per day

80a. Each time you ate **luncheon** or **deli-style ham**, how much did you usually eat?

☐ Less than 1 slice
☐ 1 to 3 slices
☐ More than 3 slices

80b. How often was the luncheon or deli-style ham you ate **light**, **low-fat**, or **fat-free**?

☐ Almost never or never
☐ About ¼ of the time
☐ About ½ of the time
☐ About ¾ of the time
☐ Almost always or always

81. How often did you eat **other cold cuts** or **luncheon meats** (such as bologna, salami, corned beef, pastrami, or others, including low-fat)? *(Please do not include ham, turkey, or chicken cold cuts.)*

☐ NEVER (GO TO QUESTION 82)

☐ 1 time in past month
☐ 2-3 times in past month
☐ 1 time per week
☐ 2 times per week
☐ 3-4 times per week
☐ 5-6 times per week
☐ 1 time per day
☐ 2 or more times per day

Question 82 appears in the next column

81a. Each time you ate **other cold cuts** or **luncheon meats**, how much did you usually eat?

☐ Less than 1 slice
☐ 1 to 3 slices
☐ More than 3 slices

81b. How often were the other cold cuts or luncheon meats you ate **light**, **low-fat**, or **fat-free**? *(Please do not include ham, turkey, or chicken cold cuts.)*

☐ Almost never or never
☐ About ¼ of the time
☐ About ½ of the time
☐ About ¾ of the time
☐ Almost always or always

82. How often did you eat **canned tuna** (including in salads, sandwiches, or casseroles)?

☐ NEVER (GO TO QUESTION 83)

☐ 1 time in past month
☐ 2-3 times in past month
☐ 1 time per week
☐ 2 times per week
☐ 3-4 times per week
☐ 5-6 times per week
☐ 1 time per day
☐ 2 or more times per day

82a. Each time you ate **canned tuna**, how much did you usually eat?

☐ Less than ¼ cup or less than 2 ounces
☐ ¼ to ½ cup or 2 to 3 ounces
☐ More than ½ cup or more than 3 ounces

82b. How often was the canned tuna you ate **water-packed**?

☐ Almost never or never
☐ About ¼ of the time
☐ About ½ of the time
☐ About ¾ of the time
☐ Almost always or always

82c. How often was the canned tuna you ate **prepared with mayonnaise** or **other dressing** (including low-fat)?

☐ Almost never or never
☐ About ¼ of the time
☐ About ½ of the time
☐ About ¾ of the time
☐ Almost always or always

Question 83 appears on the next page

Over the past month...

83. How often did you eat **GROUND chicken** or **turkey**? *(We will ask about other chicken and turkey later.)*

☐ NEVER (GO TO QUESTION 84)

☐ 1 time in past month
☐ 2-3 times in past month
☐ 1 time per week
☐ 2 times per week
☐ 3-4 times per week
☐ 5-6 times per week
☐ 1 time per day
☐ 2 or more times per day

83a. Each time you ate **GROUND chicken** or **turkey**, how much did you usually eat?

☐ Less than 2 ounces or less than ½ cup
☐ 2 to 4 ounces or ½ to 1 cup
☐ More than 4 ounces or more than 1 cup

84. How often did you eat **beef hamburgers** or **cheeseburgers** from a **FAST FOOD** or **OTHER RESTAURANT**?

☐ NEVER (GO TO QUESTION 85)

☐ 1 time in past month
☐ 2-3 times in past month
☐ 1 time per week
☐ 2 times per week
☐ 3-4 times per week
☐ 5-6 times per week
☐ 1 time per day
☐ 2 or more times per day

84a. Each time you ate **beef hamburgers** or **cheeseburgers** from a **FAST FOOD** or **OTHER RESTAURANT**, what size did you usually eat?

☐ Small hamburger (such as a regular Burger King or McDonald's Hamburger)
☐ Medium (such as McDonald's or Burger King Double Burger or Cheeseburger)
☐ Large (such as Burger King Whopper or Double Whopper or a McDonald's Double Quarter Pounder)

84b. Each time you ate **beef hamburgers** or **cheeseburgers** from a **FAST FOOD** or **OTHER RESTAURANT**, how much did you usually eat?

☐ Less than 1 burger
☐ 1 burger
☐ More than 1 burger

Question 85 appears in the next column

84c. How often did you have **cheeseburgers** rather than **hamburgers** from a **FAST FOOD** or **OTHER RESTAURANT**?

☐ Almost never or never
☐ About ¼ of the time
☐ About ½ of the time
☐ About ¾ of the time
☐ Almost always or always

85. How often did you eat **beef hamburgers** or **cheeseburgers** that were **NOT** from a **FAST FOOD** or **OTHER RESTAURANT**?

☐ NEVER (GO TO QUESTION 86)

☐ 1 time in past month
☐ 2-3 times in past month
☐ 1 time per week
☐ 2 times per week
☐ 3-4 times per week
☐ 5-6 times per week
☐ 1 time per day
☐ 2 or more times per day

85a. Each time you ate **beef hamburgers** or **cheeseburgers** that were **NOT** from a **FAST FOOD** or **OTHER RESTAURANT**, how much did you usually eat?

☐ Less than 1 patty or less than 2 ounces
☐ 1 patty or 2 to 4 ounces
☐ More than 1 patty or more than 4 ounces

85b. How often were these beef hamburgers or cheeseburgers made with **lean** or **extra lean ground beef**?

☐ Almost never or never
☐ About ¼ of the time
☐ About ½ of the time
☐ About ¾ of the time
☐ Almost always or always

86. How often did you eat **ground beef in mixtures** (such as meatballs, casseroles, chili, or meatloaf)?

☐ NEVER (GO TO QUESTION 87)

☐ 1 time in past month
☐ 2-3 times in past month
☐ 1 time per week
☐ 2 times per week
☐ 3-4 times per week
☐ 5-6 times per week
☐ 1 time per day
☐ 2 or more times per day

86a. Each time you ate **ground beef in mixtures**, how much did you usually eat?

☐ Less than 3 ounces or less than ½ cup
☐ 3 to 8 ounces or ½ to 1 cup
☐ More than 8 ounces or more than 1 cup

Question 87 appears on the next page

Over the past month...

87. How often did you eat **hot dogs, wieners** or **frankfurters**? *(Please do not include sausages or vegetarian hot dogs.)*

☐ NEVER (GO TO QUESTION 88)

☐ 1 time in past month
☐ 2-3 times in past month
☐ 1 time per week
☐ 2 times per week
☐ 3-4 times per week
☐ 5-6 times per week
☐ 1 time per day
☐ 2 or more times per day

87a. Each time you ate **hot dogs, wieners,** or **frankfurters**, how many did you usually eat?

☐ Less than 1 hot dog
☐ 1 to 2 hot dogs
☐ More than 2 hot dogs

87b. How often were the hot dogs, wieners, or frankfurters you ate **light** or **low-fat**?

☐ Almost never or never
☐ About ¼ of the time
☐ About ½ of the time
☐ About ¾ of the time
☐ Almost always or always

88. How often did you eat **beef mixtures** (such as beef stew, beef curry, beef pot pie, beef and noodles, or beef and vegetables)?

☐ NEVER (GO TO QUESTION 89)

☐ 1 time in past month
☐ 2-3 times in past month
☐ 1 time per week
☐ 2 times per week
☐ 3-4 times per week
☐ 5-6 times per week
☐ 1 time per day
☐ 2 or more times per day

88a. Each time you ate **beef mixtures**, how much did you usually eat?

☐ Less than ½ cup
☐ ½ to 1½ cups
☐ More than 1½ cups

89. How often did you eat **roast beef** or **pot roast**? *(Please do not include roast beef or pot roast in sandwiches.)*

☐ NEVER (GO TO QUESTION 90)

☐ 1 time in past month
☐ 2-3 times in past month
☐ 1 time per week
☐ 2 times per week
☐ 3-4 times per week
☐ 5-6 times per week
☐ 1 time per day
☐ 2 or more times per day

Question 90 appears in the next column

89a. Each time you ate **roast beef** or **pot roast**, how much did you usually eat?

☐ Less than 2 ounces
☐ 2 to 6 ounces
☐ More than 6 ounces

90. How often did you eat **steak** (beef)? *(Please do not include steak in sandwiches)*

☐ NEVER (GO TO QUESTION 91)

☐ 1 time in past month
☐ 2-3 times in past month
☐ 1 time per week
☐ 2 times per week
☐ 3-4 times per week
☐ 5-6 times per week
☐ 1 time per day
☐ 2 or more times per day

90a. Each time you ate **steak** (beef), how much did you usually eat?

☐ Less than 3 ounces
☐ 3 to 7 ounces
☐ More than 7 ounces

90b. How often was the steak you ate **lean steak**?

☐ Almost never or never
☐ About ¼ of the time
☐ About ½ of the time
☐ About ¾ of the time
☐ Almost always or always

91. How often did you eat **pork** or **beef spareribs**?

☐ NEVER (GO TO QUESTION 92)

☐ 1 time in past month
☐ 2-3 times in past month
☐ 1 time per week
☐ 2 times per week
☐ 3-4 times per week
☐ 5-6 times per week
☐ 1 time per day
☐ 2 or more times per day

91a. Each time you ate **pork** or **beef spareribs**, how much did you usually eat?

☐ Less than 4 ribs
☐ 4 to 12 ribs
☐ More than 12 ribs

92. How often did you eat **roast turkey**, **turkey cutlets**, or **turkey nuggets** (including in sandwiches)?

☐ NEVER (GO TO QUESTION 93)

☐ 1 time in past month
☐ 2-3 times in past month
☐ 1 time per week
☐ 2 times per week
☐ 3-4 times per week
☐ 5-6 times per week
☐ 1 time per day
☐ 2 or more times per day

Question 93 appears on the next page

Over the past month...

92a. Each time you ate **roast turkey**, **turkey cutlets**, or **turkey nuggets**, how much did you usually eat? *(Please note: 4 to 8 turkey nuggets = 3 ounces.)*

☐ Less than 2 ounces
☐ 2 to 5 ounces
☐ More than 5 ounces

93. How often did you eat **chicken mixtures** (such as salads, sandwiches, casseroles, chicken curries, stews, or other mixtures)?

☐ NEVER (GO TO QUESTION 94)

☐ 1 time in past month
☐ 2-3 times in past month
☐ 1 time per week
☐ 2 times per week
☐ 3-4 times per week
☐ 5-6 times per week
☐ 1 time per day
☐ 2 or more times per day

93a. Each time you ate **chicken mixtures**, how much did you usually eat?

☐ Less than ½ cup
☐ ½ to 1½ cups
☐ More than 1½ cups

94. How often did you eat **baked**, **broiled**, **roasted**, **stewed**, or **fried chicken** (including nuggets)? *(Please do not include chicken in mixtures.)*

☐ NEVER (GO TO QUESTION 95)

☐ 1 time in past month
☐ 2-3 times in past month
☐ 1 time per week
☐ 2 times per week
☐ 3-4 times per week
☐ 5-6 times per week
☐ 1 time per day
☐ 2 or more times per day

94a. Each time you ate **baked**, **broiled**, **roasted**, **stewed**, or **fried chicken** (including nuggets), how much did you usually eat?

☐ Less than 2 drumsticks or wings, less than 1 breast or thigh, or less than 4 nuggets
☐ 2 drumsticks or wings, 1 breast or thigh, or 4 to 8 nuggets
☐ More than 2 drumsticks or wings, more than 1 breast or thigh, or more than 8 nuggets

Question 95 appears in the next column

94b. How often was the chicken you ate **fried chicken** (including deep fried) or **chicken nuggets**?

☐ Almost never or never
☐ About ¼ of the time
☐ About ½ of the time
☐ About ¾ of the time
☐ Almost always or always

94c. How often was the chicken you ate **WHITE meat**?

☐ Almost never or never
☐ About ¼ of the time
☐ About ½ of the time
☐ About ¾ of the time
☐ Almost always or always

94d. How often did you eat chicken **WITH skin**?

☐ Almost never or never
☐ About ¼ of the time
☐ About ½ of the time
☐ About ¾ of the time
☐ Almost always or always

95. How often did you eat **baked ham** or **ham steak**?

☐ NEVER (GO TO QUESTION 96)

☐ 1 time in past month
☐ 2-3 times in past month
☐ 1 time per week
☐ 2 times per week
☐ 3-4 times per week
☐ 5-6 times per week
☐ 1 time per day
☐ 2 or more times per day

95a. Each time you ate **baked ham** or **ham steak**, how much did you usually eat?

☐ Less than 1 ounce
☐ 1 to 3 ounces
☐ More than 3 ounces

96. How often did you eat **pork** (including chops, roasts, and in mixed dishes)? *(Please do not include ham, ham steak, or sausage.)*

☐ NEVER (GO TO QUESTION 97)

☐ 1 time in past month
☐ 2-3 times in past month
☐ 1 time per week
☐ 2 times per week
☐ 3-4 times per week
☐ 5-6 times per week
☐ 1 time per day
☐ 2 or more times per day

96a. Each time you ate **pork**, how much did you usually eat?

☐ Less than 2 ounces or less than 1 chop
☐ 2 to 5 ounces or 1 chop
☐ More than 5 ounces or more than 1 chop

Question 97 appears on the next page

Over the past month...

97. How often did you eat **gravy** on meat, chicken, potatoes (**NOT** including poutine), rice, etc.?

☐ NEVER (GO TO QUESTION 98)

☐ 1 time in past month
☐ 2-3 times in past month
☐ 1 time per week
☐ 2 times per week
☐ 3-4 times per week
☐ 5-6 times per week
☐ 1 time per day
☐ 2 or more times per day

97a. Each time you ate **gravy** on meat, chicken, potatoes, rice, etc., how much did you usually eat?

☐ Less than ⅛ cup
☐ ⅛ to ½ cup
☐ More than ½ cup

98. How often did you eat **liver** (all kinds) or **liverwurst**?

☐ NEVER (GO TO QUESTION 99)

☐ 1 time in past month
☐ 2-3 times in past month
☐ 1 time per week
☐ 2 times per week
☐ 3-4 times per week
☐ 5-6 times per week
☐ 1 time per day
☐ 2 or more times per day

98a. Each time you ate **liver** or **liverwurst**, how much did you usually eat?

☐ Less than ½ ounce
☐ ½ to 3 ounces
☐ More than 3 ounces

99. How often did you eat **bacon** (including low-fat)?

☐ NEVER (GO TO QUESTION 100)

☐ 1 time in past month
☐ 2-3 times in past month
☐ 1 time per week
☐ 2 times per week
☐ 3-4 times per week
☐ 5-6 times per week
☐ 1 time per day
☐ 2 or more times per day

99a. Each time you ate **bacon**, how much did you usually eat?

☐ Fewer than 2 slices
☐ 2 to 4 slices
☐ More than 4 slices

Question 100 appears in the next column

99b. How often was the bacon you ate **light**, **low-fat**, or **lean**?

☐ Almost never or never
☐ About ¼ of the time
☐ About ½ of the time
☐ About ¾ of the time
☐ Almost always or always

100. How often did you eat **sausage** (including low-fat)?

☐ NEVER (GO TO QUESTION 101)

☐ 1 time in past month
☐ 2-3 times in past month
☐ 1 time per week
☐ 2 times per week
☐ 3-4 times per week
☐ 5-6 times per week
☐ 1 time per day
☐ 2 or more times per day

100a. Each time you ate **sausage**, how much did you usually eat?

☐ Less than 2 small links or less than 1 large link
☐ 2 to 5 small links or 1 to 2 large links
☐ More than 5 small links or more than 2 large links

100b. How often was the sausage you ate **light**, **low-fat**, or **lean**?

☐ Almost never or never
☐ About ¼ of the time
☐ About ½ of the time
☐ About ¾ of the time
☐ Almost always or always

101. How often did you eat **fried shellfish** (such as crab, lobster, scallops, or shrimp)?

☐ NEVER (GO TO QUESTION 102)

☐ 1 time in past month
☐ 2-3 times in past month
☐ 1 time per week
☐ 2 times per week
☐ 3-4 times per week
☐ 5-6 times per week
☐ 1 time per day
☐ 2 or more times per day

101a. Each time you ate **fried shellfish**, how much did you usually eat?

☐ Less than 1 ounce
☐ 1 to 3 ounces
☐ More than 3 ounces

Question 102 appears on the next page

Over the <u>past month</u>...

102. How often did you eat **shellfish** (such as crab, lobster, scallops, or shrimp) **that was NOT FRIED**?

- ☐ NEVER (GO TO QUESTION 103)
- ☐ 1 time in past month
- ☐ 2-3 times in past month
- ☐ 1 time per week
- ☐ 2 times per week
- ☐ 3-4 times per week
- ☐ 5-6 times per week
- ☐ 1 time per day
- ☐ 2 or more times per day

102a. Each time you ate **shellfish that was NOT FRIED**, how much did you usually eat?

- ☐ Less than 1 ounce
- ☐ 1 to 4 ounces
- ☐ More than 4 ounces

103. How often did you eat **dark** or **oily fish** like **salmon, fresh tuna, trout,** or **mackerel**?

- ☐ NEVER (GO TO QUESTION 104)
- ☐ 1 time in past month
- ☐ 2-3 times in past month
- ☐ 1 time per week
- ☐ 2 times per week
- ☐ 3-4 times per week
- ☐ 5-6 times per week
- ☐ 1 time per day
- ☐ 2 or more times per day

103a. Each time you ate **dark** or **oily fish**, how much did you usually eat?

- ☐ Less than 2 ounces
- ☐ 2 to 6 ounces
- ☐ More than 6 ounces

104. How often did you eat **ready-to-eat battered fish** or **fish sticks**, including in fast food sandwiches (not including shellfish)?

- ☐ NEVER (GO TO QUESTION 105)
- ☐ 1 time in past month
- ☐ 2-3 times in past month
- ☐ 1 time per week
- ☐ 2 times per week
- ☐ 3-4 times per week
- ☐ 5-6 times per week
- ☐ 1 time per day
- ☐ 2 or more times per day

104a. Each time you ate **ready-to-eat battered fish** or **fish sticks,** how much did you usually eat?

- ☐ Less than 2 ounces or less than 1 fillet
- ☐ 2 to 6 ounces or 1 fillet
- ☐ More than 6 ounces or more than 1 fillet

Question 105 appears in the next column

105. How often did you eat **white** or **lean fish** like **cod, sole, perch,** or **pike** (do not include ready-to-eat battered fish or fish sticks)?

- ☐ NEVER (GO TO INTRODUCTION TO QUESTION 106)
- ☐ 1 time in past month
- ☐ 2-3 times in past month
- ☐ 1 time per week
- ☐ 2 times per week
- ☐ 3-4 times per week
- ☐ 5-6 times per week
- ☐ 1 time per day
- ☐ 2 or more times per day

105a. Each time you ate **white** or **lean fish**, how much did you usually eat?

- ☐ Less than 2 ounces or less than 1 fillet
- ☐ 2 to 5 ounces or 1 fillet
- ☐ More than 5 ounces or more than 1 fillet

Now think about all the meat, poultry, and fish you ate in the <u>past month</u> and how they were prepared.

106. How often was **oil**, **butter**, **margarine**, or **other fat** used to **FRY**, **SAUTE**, **BASTE**, OR **MARINATE** any meat, poultry, or fish you ate? *(Please do not include deep frying.)*

- ☐ NEVER (GO TO QUESTION 107)
- ☐ 1 time in past month
- ☐ 2-3 times in past month
- ☐ 1 time per week
- ☐ 2 times per week
- ☐ 3-4 times per week
- ☐ 5-6 times per week
- ☐ 1 time per day
- ☐ 2 or more times per day

106a. Which of the following **fats** were regularly used to prepare your meat, poultry, or fish? ***(Mark all that apply.)***

- ☐ Margarine (including light)
- ☐ Butter (including light)
- ☐ Lard, fatback, or bacon fat
- ☐ Olive oil
- ☐ Corn oil
- ☐ Canola or rapeseed oil
- ☐ Oil spray, such as Pam or others
- ☐ Other kinds of oils
- ☐ None of the above

Question 107 appears on the next page

Over the <u>past month</u>...

107. Thinking about the **MEAT** you ate, how often was it cooked by **broiling**, **grilling**, **barbecuing,** or **pan-frying**? *(Do not include poultry or fish).*

- ☐ NEVER (GO TO QUESTION 108)
- ☐ 1 time in past month
- ☐ 2-3 times in past month
- ☐ 1 time per week
- ☐ 2 times per week
- ☐ 3-4 times per week
- ☐ 5-6 times per week
- ☐ 1 time per day
- ☐ 2 or more times per day

107a. Each time you ate meat that was cooked by broiling, grilling, barbecuing, or pan-frying, what was the **outside appearance** of the meat?

- ☐ Light brown
- ☐ Medium brown
- ☐ Heavily browned or blackened

107b. Each time you ate meat that was cooked by broiling, grilling, barbecuing, or pan-frying, what was the **inside appearance** of the meat?

- ☐ Red (rare)
- ☐ Pink (medium)
- ☐ Brown (well-done)

108. How often did you eat **tofu**?

- ☐ NEVER (GO TO QUESTION 109)
- ☐ 1 time in past month
- ☐ 2-3 times in past month
- ☐ 1 time per week
- ☐ 2 times per week
- ☐ 3-4 times per week
- ☐ 5-6 times per week
- ☐ 1 time per day
- ☐ 2 or more times per day

108a. Each time you ate **tofu**, how much did you usually eat?

- ☐ Less than ¼ cup or less than 2 ounces
- ☐ ¼ to ¾ cup or 2 to 6 ounces
- ☐ More than ¾ cup or more than 6 ounces

Question 109 appears in the next column

109. How often did you eat **soy burgers** or **soy meat-substitutes**?

- ☐ NEVER (GO TO QUESTION 110)
- ☐ 1 time in past month
- ☐ 2-3 times in past month
- ☐ 1 time per week
- ☐ 2 times per week
- ☐ 3-4 times per week
- ☐ 5-6 times per week
- ☐ 1 time per day
- ☐ 2 or more times per day

109a. Each time you ate **soy burgers** or **soy meat-substitutes**, how much did you usually eat?

- ☐ Less than ¼ cup or less than 2 ounces
- ☐ ¼ to 1 cup or 2 to 8 ounces
- ☐ More than 1 cup or more than 8 ounces

110. How often did you eat **soup**?

- ☐ NEVER (GO TO QUESTION 111)
- ☐ 1 time in past month
- ☐ 2-3 times in past month
- ☐ 1 time per week
- ☐ 2 times per week
- ☐ 3-4 times per week
- ☐ 5-6 times per week
- ☐ 1 time per day
- ☐ 2 or more times per day

110a. Each time you ate **soup**, how much did you usually eat?

- ☐ Less than 1 cup
- ☐ 1 to 2 cups
- ☐ More than 2 cups

110b. How often were the soups you ate **bean, pea,** or **lentil soups**?

- ☐ Almost never or never
- ☐ About ¼ of the time
- ☐ About ½ of the time
- ☐ About ¾ of the time
- ☐ Almost always or always

110c. How often were the soups you ate **cream soups** (including chowders)?

- ☐ Almost never or never
- ☐ About ¼ of the time
- ☐ About ½ of the time
- ☐ About ¾ of the time
- ☐ Almost always or always

Question 111 appears on the next page

Over the <u>past month</u>...

110d. How often were the soups you ate **tomato** or **vegetable soups** (NOT cream soups)?

- ☐ Almost never or never
- ☐ About ¼ of the time
- ☐ About ½ of the time
- ☐ About ¾ of the time
- ☐ Almost always or always

110e. How often were the soups you ate **broth soups** (including chicken) **with** or **without noodles** or **rice**?

- ☐ Almost never or never
- ☐ About ¼ of the time
- ☐ About ½ of the time
- ☐ About ¾ of the time
- ☐ Almost always or always

111. How often did you eat **pizza**?

- ☐ NEVER (GO TO QUESTION 112)
- ☐ 1 time in past month
- ☐ 2-3 times in past month
- ☐ 1 time per week
- ☐ 2 times per week
- ☐ 3-4 times per week
- ☐ 5-6 times per week
- ☐ 1 time per day
- ☐ 2 or more times per day

111a. Each time you ate **pizza**, how much did you usually eat?

- ☐ Less than 1 slice or less than 1 mini pizza
- ☐ 1 to 3 slices or 1 to 2 mini pizzas
- ☐ More than 3 slices or more than 2 mini pizzas

111b. How often did you eat pizza with **pepperoni**, **sausage**, or **other meat**?

- ☐ Almost never or never
- ☐ About ¼ of the time
- ☐ About ½ of the time
- ☐ About ¾ of the time
- ☐ Almost always or always

112. How often did you eat **crackers**?

- ☐ NEVER (GO TO QUESTION 113)
- ☐ 1 time in past month
- ☐ 2-3 times in past month
- ☐ 1 time per week
- ☐ 2 times per week
- ☐ 3-4 times per week
- ☐ 5-6 times per week
- ☐ 1 time per day
- ☐ 2 or more times per day

112a. Each time you ate **crackers**, how many did you usually eat?

- ☐ Fewer than 4 crackers
- ☐ 4 to 8 crackers
- ☐ More than 8 crackers

Question 113 appears in the next column

113. How often did you eat **corn bread** or **corn muffins**?

- ☐ NEVER (GO TO QUESTION 114)
- ☐ 1 time in past month
- ☐ 2-3 times in past month
- ☐ 1 time per week
- ☐ 2 times per week
- ☐ 3-4 times per week
- ☐ 5-6 times per week
- ☐ 1 time per day
- ☐ 2 or more times per day

113a. Each time you ate **corn bread** or **corn muffins**, how much did you usually eat?

- ☐ Less than 1 piece or muffin
- ☐ 1 to 2 pieces or muffins
- ☐ More than 2 pieces or muffins

114. How often did you eat **baking powder biscuits,** including **scones** or **tea biscuits**?

- ☐ NEVER (GO TO QUESTION 115)
- ☐ 1 time in past month
- ☐ 2-3 times in past month
- ☐ 1 time per week
- ☐ 2 times per week
- ☐ 3-4 times per week
- ☐ 5-6 times per week
- ☐ 1 time per day
- ☐ 2 or more times per day

114a. Each time you ate **baking powder biscuits** including **scones** or **tea biscuits**, how many did you usually eat?

- ☐ Fewer than 1 biscuit
- ☐ 1 to 2 biscuits
- ☐ More than 2 biscuits

115. How often did you eat **potato chips** (including low-fat, baked, or low-salt)?

- ☐ NEVER (GO TO QUESTION 116)
- ☐ 1 time in past month
- ☐ 2-3 times in past month
- ☐ 1 time per week
- ☐ 2 times per week
- ☐ 3-4 times per week
- ☐ 5-6 times per week
- ☐ 1 time per day
- ☐ 2 or more times per day

115a. Each time you ate **potato chips**, how much did you usually eat?

- ☐ Fewer than 10 chips or less than 1 cup
- ☐ 10 to 25 chips or 1 to 2 cups
- ☐ More than 25 chips or more than 2 cups

Question 116 appears on the next page

Over the <u>past month</u>...

116. How often did you eat **corn chips** or **tortilla chips** (including low-fat, baked, or low-salt)?

- ☐ NEVER (GO TO QUESTION 117)
- ☐ 1 time in past month
- ☐ 2-3 times in past month
- ☐ 1 time per week
- ☐ 2 times per week
- ☐ 3-4 times per week
- ☐ 5-6 times per week
- ☐ 1 time per day
- ☐ 2 or more times per day

116a. Each time you ate **corn chips** or **tortilla chips**, how much did you usually eat?

- ☐ Fewer than 10 chips or less than 1 cup
- ☐ 10 to 25 chips or 1 to 2 cups
- ☐ More than 25 chips or more than 2 cups

117. How often did you eat **popcorn** (including low-fat)?

- ☐ NEVER (GO TO QUESTION 118)
- ☐ 1 time in past month
- ☐ 2-3 times in past month
- ☐ 1 time per week
- ☐ 2 times per week
- ☐ 3-4 times per week
- ☐ 5-6 times per week
- ☐ 1 time per day
- ☐ 2 or more times per day

117a. Each time you ate **popcorn**, how much did you usually eat?

- ☐ Less than 1½ cups, popped
- ☐ 1½ to 5 cups, popped
- ☐ More than 5 cups, popped

118. How often did you eat **pretzels**?

- ☐ NEVER (GO TO QUESTION 119)
- ☐ 1 time in past month
- ☐ 2-3 times in past month
- ☐ 1 time per week
- ☐ 2 times per week
- ☐ 3-4 times per week
- ☐ 5-6 times per week
- ☐ 1 time per day
- ☐ 2 or more times per day

118a. Each time you ate **pretzels**, how many did you usually eat?

- ☐ Fewer than 7 average twists
- ☐ 7 to 20 average twists
- ☐ More than 20 average twists

Question 119 appears in the next column

119. How often did you eat **peanuts**, **walnuts**, **almonds**, or **other nuts**?

- ☐ NEVER (GO TO QUESTION 120)
- ☐ 1 time in past month
- ☐ 2-3 times in past month
- ☐ 1 time per week
- ☐ 2 times per week
- ☐ 3-4 times per week
- ☐ 5-6 times per week
- ☐ 1 time per day
- ☐ 2 or more times per day

119a. Each time you ate **peanuts**, **walnuts**, **almonds,** or **other nuts**, how much did you usually eat?

- ☐ Less than ¼ cup
- ☐ ¼ to ½ cup
- ☐ More than ½ cup

119b. How often were the nuts you ate **peanuts**?

- ☐ Almost never or never
- ☐ About ¼ of the time
- ☐ About ½ of the time
- ☐ About ¾ of the time
- ☐ Almost always or always

120. How often did you eat **flaxseeds**?

- ☐ NEVER (GO TO QUESTION 121)
- ☐ 1 time in past month
- ☐ 2-3 times in past month
- ☐ 1 time per week
- ☐ 2 times per week
- ☐ 3-4 times per week
- ☐ 5-6 times per week
- ☐ 1 time per day
- ☐ 2 or more times per day

120a. Each time you ate **flaxseeds**, how much did you usually eat?

- ☐ Less than 1 tablespoon
- ☐ 1 to 2 tablespoons
- ☐ More than 2 tablespoons

121. How often did you eat **other seeds**, like **sunflower** or **pumpkin seeds**?

- ☐ NEVER (GO TO QUESTION 122)
- ☐ 1 time in past month
- ☐ 2-3 times in past month
- ☐ 1 time per week
- ☐ 2 times per week
- ☐ 3-4 times per week
- ☐ 5-6 times per week
- ☐ 1 time per day
- ☐ 2 or more times per day

121a. Each time you ate **other seeds**, how much did you usually eat?

- ☐ Less than 2 tablespoons
- ☐ 2 tablespoons to ½ cup
- ☐ More than ½ cup

Question 122 appears on the next page

Over the past month...

122. How often did you eat **energy** or **high-protein bars**, (such as Power Bars, Vector, Clif, Luna, Isoflex or others)?

☐ NEVER (GO TO QUESTION 123)

☐ 1 time in past month
☐ 2-3 times in past month
☐ 1 time per week
☐ 2 times per week
☐ 3-4 times per week
☐ 5-6 times per week
☐ 1 time per day
☐ 2 or more times per day

122a. Each time you ate **energy** or **high-protein bars**, how much did you usually eat?

☐ Less than 1 bar
☐ 1 bar
☐ More than 1 bar

123. How often did you eat **yogurt** (NOT including frozen yogurt)?

☐ NEVER (GO TO QUESTION 124)

☐ 1 time in past month
☐ 2-3 times in past month
☐ 1 time per week
☐ 2 times per week
☐ 3-4 times per week
☐ 5-6 times per week
☐ 1 time per day
☐ 2 or more times per day

123a. Each time you ate **yogurt**, how much did you usually eat?

☐ Less than ½ cup or less than 1 container
☐ ½ to ¾ cup or 1 container
☐ More than ¾ cup or more than 1 container

123b. How often was the **yogurt** you ate **low-fat** or **fat-free**?

☐ Almost never or never
☐ About ¼ of the time
☐ About ½ of the time
☐ About ¾ of the time
☐ Almost always or always

124. How often did you eat **cottage cheese** (including low-fat)?

☐ NEVER (GO TO QUESTION 125)

☐ 1 time in past month
☐ 2-3 times in past month
☐ 1 time per week
☐ 2 times per week
☐ 3-4 times per week
☐ 5-6 times per week
☐ 1 time per day
☐ 2 or more times per day

Question 125 appears in the next column

124a. Each time you ate **cottage cheese**, how much did you usually eat?

☐ Less than ¼ cup
☐ ¼ to ¾ cup
☐ More than ¾ cup

125. How often did you eat **cheese** (including low-fat; including on cheeseburgers or in sandwiches or subs, NOT including cream cheese)?

☐ NEVER (GO TO QUESTION 126)

☐ 1 time in past month
☐ 2-3 times in past month
☐ 1 time per week
☐ 2 times per week
☐ 3-4 times per week
☐ 5-6 times per week
☐ 1 time per day
☐ 2 or more times per day

125a. Each time you ate **cheese**, how much did you usually eat?

☐ Less than ½ ounce or less than 1 slice
☐ ½ to 2 ounces or 1 to 2 slices
☐ More than 2 ounces or more than 2 slices

125b. How often was the cheese you ate **low-fat**?

☐ Almost never or never
☐ About ¼ of the time
☐ About ½ of the time
☐ About ¾ of the time
☐ Almost always or always

126. How often did you eat **frozen yogurt**, **sorbet**, or **ices** (including low-fat or fat-free)?

☐ NEVER (GO TO QUESTION 127)

☐ 1 time in past month
☐ 2-3 times in past month
☐ 1 time per week
☐ 2 times per week
☐ 3-4 times per week
☐ 5-6 times per week
☐ 1 time per day
☐ 2 or more times per day

126a. Each time you ate **frozen yogurt**, **sorbet**, or **ices**, how much did you usually eat?

☐ Less than ½ cup or less than 1 scoop
☐ ½ to 1 cup or 1 to 2 scoops
☐ More than 1 cup or more than 2 scoops

Question 127 appears on the next page

Over the past month...

127. How often did you eat **ice cream** or **ice cream bars** (including low-fat or fat-free)?

☐ NEVER (GO TO QUESTION 128)

☐ 1 time in past month
☐ 2-3 times in past month
☐ 1 time per week
☐ 2 times per week
☐ 3-4 times per week
☐ 5-6 times per week
☐ 1 time per day
☐ 2 or more times per day

127a. Each time you ate **ice cream** or **ice cream bars**, how much did you usually eat?

☐ Less than ½ cup or less than 1 scoop
☐ ½ to 1 cup or 1 to 2 scoops
☐ More than 1 cup or more than 2 scoops

127b. How often was the ice cream you ate **light**, **low-fat**, or **fat-free**?

☐ Almost never or never
☐ About ¼ of the time
☐ About ½ of the time
☐ About ¾ of the time
☐ Almost always or always

128. How often did you eat **cake** (including low-fat or fat-free)?

☐ NEVER (GO TO QUESTION 129)

☐ 1 time in past month
☐ 2-3 times in past month
☐ 1 time per week
☐ 2 times per week
☐ 3-4 times per week
☐ 5-6 times per week
☐ 1 time per day
☐ 2 or more times per day

128a. Each time you ate **cake**, how much did you usually eat?

☐ Less than 1 medium piece
☐ 1 medium piece
☐ More than 1 medium piece

Question 129 appears in the next column

129. How often did you eat **cookies** or **brownies** (including low-fat or fat-free)?

☐ NEVER (GO TO QUESTION 130)

☐ 1 time in past month
☐ 2-3 times in past month
☐ 1 time per week
☐ 2 times per week
☐ 3-4 times per week
☐ 5-6 times per week
☐ 1 time per day
☐ 2 or more times per day

129a. Each time you ate **cookies** or **brownies**, how much did you usually eat?

☐ Less than 2 cookies or 1 small brownie
☐ 2 to 4 cookies or 1 medium brownie
☐ More than 4 cookies or 1 large brownie

130. How often did you eat **doughnuts**, **sweet rolls**, **Danish**, or **Pop-Tarts**?

☐ NEVER (GO TO QUESTION 131)

☐ 1 time in past month
☐ 2-3 times in past month
☐ 1 time per week
☐ 2 times per week
☐ 3-4 times per week
☐ 5-6 times per week
☐ 1 time per day
☐ 2 or more times per day

130a. Each time you ate **doughnuts**, **sweet rolls**, **Danish**, or **Pop-Tarts**, how much did you usually eat?

☐ Less than 1 piece
☐ 1 to 2 pieces
☐ More than 2 pieces

131. How often did you eat **sweet muffins** or **dessert breads** (such as banana bread, blueberry muffins, or lemon loaf, including low-fat or fat-free)?

☐ NEVER (GO TO QUESTION 132)

☐ 1 time in past month
☐ 2-3 times in past month
☐ 1 time per week
☐ 2 times per week
☐ 3-4 times per week
☐ 5-6 times per week
☐ 1 time per day
☐ 2 or more times per day

131a. Each time you ate **sweet muffins** or **dessert breads**, how much did you usually eat?

☐ Less than 1 medium piece
☐ 1 medium piece
☐ More than 1 medium piece

Question 132 appears on the next page

Over the past month...

132. How often did you eat **fruit crisp**, **cobbler**, or **strudel**?

☐ NEVER (GO TO QUESTION 133)

☐ 1 time in past month
☐ 2-3 times in past month
☐ 1 time per week
☐ 2 times per week
☐ 3-4 times per week
☐ 5-6 times per week
☐ 1 time per day
☐ 2 or more times per day

132a. Each time you ate **fruit crisp**, **cobbler**, or **strudel**, how much did you usually eat?

☐ Less than ½ cup
☐ ½ to 1 cup
☐ More than 1 cup

133. How often did you eat **pie**?

☐ NEVER (GO TO QUESTION 134)

☐ 1 time in past month
☐ 2-3 times in past month
☐ 1 time per week
☐ 2 times per week
☐ 3-4 times per week
☐ 5-6 times per week
☐ 1 time per day
☐ 2 or more times per day

133a. Each time you ate **pie**, how much did you usually eat?

☐ Less than ⅛ of a pie
☐ About ⅛ of a pie
☐ More than ⅛ of a pie

The next four questions ask about the kinds of pie you ate. Please read all four questions before answering.

133b. How often were the pies you ate **fruit pie** (such as apple, blueberry, others)?

☐ Almost never or never
☐ About ¼ of the time
☐ About ½ of the time
☐ About ¾ of the time
☐ Almost always or always

133c. How often were the pies you ate **cream**, **pudding**, **custard**, or **meringue pie**?

☐ Almost never or never
☐ About ¼ of the time
☐ About ½ of the time
☐ About ¾ of the time
☐ Almost always or always

Question 134 appears in the next column

133d. How often were the pies you ate **pumpkin** or **sweet potato pie**?

☐ Almost never or never
☐ About ¼ of the time
☐ About ½ of the time
☐ About ¾ of the time
☐ Almost always or always

133e. How often were the pies you ate **pecan pie**?

☐ Almost never or never
☐ About ¼ of the time
☐ About ½ of the time
☐ About ¾ of the time
☐ Almost always or always

134. How often did you eat **chocolate candy**?

☐ NEVER (GO TO QUESTION 135)

☐ 1 time in past month
☐ 2-3 times in past month
☐ 1 time per week
☐ 2 times per week
☐ 3-4 times per week
☐ 5-6 times per week
☐ 1 time per day
☐ 2 or more times per day

134a. Each time you ate **chocolate candy**, how much did you usually eat?

☐ Less than 1 average bar or less than 1 ounce
☐ 1 average bar or 1 to 2 ounces
☐ More than 1 average bar or more than 2 ounces

135. How often did you eat **other candy**?

☐ NEVER (GO TO QUESTION 136)

☐ 1 time in past month
☐ 2-3 times in past month
☐ 1 time per week
☐ 2 times per week
☐ 3-4 times per week
☐ 5-6 times per week
☐ 1 time per day
☐ 2 or more times per day

135a. Each time you ate **other candy**, how much did you usually eat?

☐ Fewer than 2 pieces
☐ 2 to 6 pieces
☐ More than 6 pieces

Question 136 appears on the next page

Over the past month...

136. How often did you eat **eggs**, **egg whites**, or **egg substitutes** (NOT counting eggs in baked goods or desserts)? *(Please include eggs in salads, quiche, soufflés, and sandwiches.)*

☐ NEVER (GO TO QUESTION 137)

☐ 1 time in past month
☐ 2-3 times in past month
☐ 1 time per week
☐ 2 times per week
☐ 3-4 times per week
☐ 5-6 times per week
☐ 1 time per day
☐ 2 or more times per day

136a. Each time you ate **eggs**, how many did you usually eat?

☐ 1 egg
☐ 2 eggs
☐ 3 or more eggs

136b. How often were the eggs you ate **egg substitutes** or **egg whites only**?

☐ Almost never or never
☐ About ¼ of the time
☐ About ½ of the time
☐ About ¾ of the time
☐ Almost always or always

136c. How often were the eggs you ate **regular whole eggs**?

☐ Almost never or never
☐ About ¼ of the time
☐ About ½ of the time
☐ About ¾ of the time
☐ Almost always or always

136d. How often were the eggs you ate **cooked in oil**, **butter**, or **margarine**?

☐ Almost never or never
☐ About ¼ of the time
☐ About ½ of the time
☐ About ¾ of the time
☐ Almost always or always

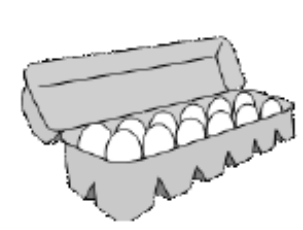

Question 137 appears in the next column

136e. How often were the eggs you ate part of **egg salad**?

☐ Almost never or never
☐ About ¼ of the time
☐ About ½ of the time
☐ About ¾ of the time
☐ Almost always or always

137. How many cups of **coffee**, caffeinated or decaffeinated, did you drink? *(Please do not include coffee drinks such as Latte, Mocha, Cappuccino, or Frappuccino.)*

☐ NONE (GO TO QUESTION 138)

☐ Less than 1 cup in past month
☐ 1-3 cups in past month
☐ 1 cup per week
☐ 2-4 cups per week
☐ 5-6 cups per week
☐ 1 cup per day
☐ 2-3 cups per day
☐ 4-5 cups per day
☐ 6 or more cups per day

137a. How often was the coffee you drank **decaffeinated**?

☐ Almost never or never
☐ About ¼ of the time
☐ About ½ of the time
☐ About ¾ of the time
☐ Almost always or always

138. How often did you drink **coffee drinks**, such as **Latte**, **Mocha**, **Cappuccino**, or **Frappuccino** (including caffeinated or decaffeinated, hot or cold)?

☐ NEVER (GO TO QUESTION 139)

☐ 1 time in past month
☐ 2-3 times in past month
☐ 1 time per week
☐ 2-4 times per week
☐ 5-6 times per week
☐ 1 time per day
☐ 2-3 times per day
☐ 4-5 times per day
☐ 6 or more times per day

138a. Each time you drank **coffee drinks**, how much did you usually drink?

☐ Less than 8 ounces (250 ml)
☐ 8 to 16 ounces (250 to 500 ml)
☐ More than 16 ounces (more than 500 ml)

138b. How often were the coffee drinks you drank **decaffeinated**?

☐ Almost never or never
☐ About ¼ of the time
☐ About ½ of the time
☐ About ¾ of the time
☐ Almost always or always

Question 139 appears on the next page

Over the past month...

139. How many glasses, cans, or bottles of **COLD** or **ICED tea**, caffeinated or decaffeinated, did you drink?

☐ NONE (GO TO QUESTION 140)

☐ Less than 1 glass, can or bottle in past month
☐ 1-3 glasses, cans or bottles in past month
☐ 1 glass, can or bottle per week
☐ 2-4 glasses, cans or bottles per week
☐ 5-6 glasses, cans or bottles per week
☐ 1 glass, can or bottle per day
☐ 2-3 glasses, cans or bottles per day
☐ 4-5 glasses, cans or bottles per day
☐ 6 or more glasses, cans or bottles per day

139a. How often was the cold or iced tea you drank **decaffeinated** or **herbal**?

☐ Almost never or never
☐ About ¼ of the time
☐ About ½ of the time
☐ About ¾ of the time
☐ Almost always or always

139b. How often was the cold or iced tea you drank **presweetened** with **sugar** or **artificial sweeteners** (such as Splenda, Equal, Sweet'N Low, or others)?

☐ Almost never or never (GO TO QUESTION 140)
☐ About ¼ of the time
☐ About ½ of the time
☐ About ¾ of the time
☐ Almost always or always

139c. What kind of **sweetener** was added to your presweetened cold or iced tea most of the time?

☐ Sugar or honey
☐ Artificial sweeteners (such as Splenda, Equal, Sweet'N Low, or others)

Question 140 appears in the next column

140. How many cups of **HOT tea**, caffeinated or decaffeinated (including herbal), did you drink?

☐ NONE (GO TO QUESTION 141)

☐ Less than 1 cup in past month
☐ 1-3 cups in past month
☐ 1 cup per week
☐ 2-4 cups per week
☐ 5-6 cups per week
☐ 1 cup per day
☐ 2-3 cups per day
☐ 4-5 cups per day
☐ 6 or more cups per day

140a. How often was the hot tea you drank **herbal**?

☐ Almost never or never
☐ About ¼ of the time
☐ About ½ of the time
☐ About ¾ of the time
☐ Almost always or always

140b. How often was the hot tea you drank **green tea**?

☐ Almost never or never
☐ About ¼ of the time
☐ About ½ of the time
☐ About ¾ of the time
☐ Almost always or always

140c. How often was the hot black tea and/or the green tea you drank **decaffeinated**?

☐ Almost never or never
☐ About ¼ of the time
☐ About ½ of the time
☐ About ¾ of the time
☐ Almost always or always

141. Over the past month, did you add **sugar**, **honey** or **other sweeteners** to your tea or coffee (hot or iced)?

☐ NO (GO TO QUESTION 142)
☐ YES

141a. How often did you add **sugar** or **honey** to your coffee or tea (hot or iced)?

☐ Almost never or never (GO TO QUESTION 141c)
☐ About ¼ of the time
☐ About ½ of the time
☐ About ¾ of the time
☐ Almost always or always

Question 141c appears on the next page
Question 142 appears on the next page

Over the past month...

141b. Each time **sugar** or **honey** was added to your coffee or tea, how much was usually added?

☐ Less than 1 teaspoon
☐ 1 to 3 teaspoons
☐ More than 3 teaspoons

141c. How often did you add **artificial sweetener** (such as Splenda, Equal, Sweet'N Low, or others) to your coffee or tea?

☐ Almost never or never (GO TO QUESTION 142)
☐ About ¼ of the time
☐ About ½ of the time
☐ About ¾ of the time
☐ Almost always or always

141d. What kind of **artificial sweetener** did you usually use?

☐ Equal, NutraSweet, or aspartame
☐ Sweet'N Low or cyclamate
☐ Splenda or sucralose
☐ Hermesetas or saccharin
☐ Herbal sweeteners like Stevia

141e. Each time **artificial sweetener** was added to your coffee or tea, how much was usually added?

☐ Less than 1 packet or less than 1 teaspoon
☐ 1 packet or 1 teaspoon
☐ More than 1 packet or more than 1 teaspoon

142. Over the past month, did you add **whiteners** (such as cream, milk, or non-dairy creamer) to your tea or coffee?

☐ NO (GO TO QUESTION 143)
☐ YES

142a. How often was **non-dairy creamer** added to your coffee or tea?

☐ Almost never or never (GO TO QUESTION 142d)
☐ About ¼ of the time
☐ About ½ of the time
☐ About ¾ of the time
☐ Almost always or always

Question 142d appears in the next column
Question 143 appears on the next page

142b. Each time **non-dairy creamer** was added to your coffee or tea, how much was usually used?

☐ Less than 1 teaspoon
☐ 1 to 3 teaspoons
☐ More than 3 teaspoons

142c. What kind of **non-dairy creamer** did you usually use?

☐ Regular powdered
☐ Low-fat or fat-free powdered
☐ Regular liquid
☐ Low-fat or fat-free liquid

142d. How often was **cream** or **half and half** added to your coffee or tea?

☐ Almost never or never (GO TO QUESTION 142f)
☐ About ¼ of the time
☐ About ½ of the time
☐ About ¾ of the time
☐ Almost always or always

142e. Each time **cream** or **half and half** was added to your coffee or tea, how much was usually added?

☐ Less than 1 tablespoon
☐ 1 to 2 tablespoons
☐ More than 2 tablespoons

142f. How often was **milk** added to your coffee or tea?

☐ Almost never or never (GO TO QUESTION 143)
☐ About ¼ of the time
☐ About ½ of the time
☐ About ¾ of the time
☐ Almost always or always

142g. Each time **milk** was added to your coffee or tea, how much was usually added?

☐ Less than 1 tablespoon
☐ 1 to 3 tablespoons
☐ More than 3 tablespoons

142h. What kind of **milk** was usually added to your coffee or tea?

☐ Whole milk
☐ 2% milk
☐ 1% milk
☐ Skim, nonfat, or 0.5% fat milk
☐ Evaporated or condensed (canned) milk
☐ Soy milk
☐ Rice milk
☐ Almond milk
☐ Other

Question 143 appears on the next page

Over the past month...

143. How often was **sugar** or **honey** added to foods you ate (such as on cereal, fruit, or yogurt)? (*Please do not include sugar in coffee, tea, other beverages, or baked goods.*)

☐ NEVER (GO TO INTRODUCTION TO QUESTION 144)

☐ 1 time in past month
☐ 2-3 times in past month
☐ 1 time per week
☐ 2 times per week
☐ 3-4 times per week
☐ 5-6 times per week
☐ 1 time per day
☐ 2 or more times per day

143a. Each time **sugar** or **honey** was added to foods you ate, how much was usually added?

☐ Less than 1 teaspoon
☐ 1 to 2 teaspoons
☐ More than 2 teaspoons

The following questions are about the kinds of margarine, mayonnaise, sour cream, cream cheese, and salad dressing that you ate. If possible, please check the labels of these foods to help you answer.

144. Over the past month, did you eat **margarine**?

☐ NO (GO TO QUESTION 145)
☐ YES

144a. How often was the margarine you ate **light** or **low-fat** (stick or tub)?

☐ Almost never or never
☐ About ¼ of the time
☐ About ½ of the time
☐ About ¾ of the time
☐ Almost always or always

145. Over the past month, did you eat **butter**?

☐ NO (GO TO QUESTION 146)
☐ YES

145a. How often was the butter you ate **light** or **low-fat**?

☐ Almost never or never
☐ About ¼ of the time
☐ About ½ of the time
☐ About ¾ of the time
☐ Almost always or always

Question 146 appears in the next column

146. Over the past month, did you eat **mayonnaise** or **mayonnaise-type dressing**?

☐ NO (GO TO QUESTION 147)
☐ YES

146a. How often was the mayonnaise you ate **light**, **low-fat** or **fat-free**?

☐ Almost never or never
☐ About ¼ of the time
☐ About ½ of the time
☐ About ¾ of the time
☐ Almost always or always

147. Over the past month, did you eat **sour cream**?

☐ NO (GO TO QUESTION 148)
☐ YES

147a. How often was the sour cream you ate **light**, **low-fat**, or **fat-free**?

☐ Almost never or never
☐ About ¼ of the time
☐ About ½ of the time
☐ About ¾ of the time
☐ Almost always or always

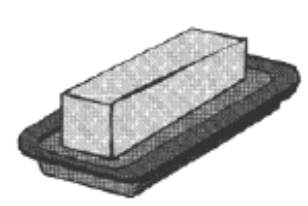

148. Over the past month, did you eat **cream cheese**?

☐ NO (GO TO QUESTION 149)
☐ YES

148a. How often was the cream cheese you ate **light**, **low-fat**, or **fat-free**?

☐ Almost never or never
☐ About ¼ of the time
☐ About ½ of the time
☐ About ¾ of the time
☐ Almost always or always

Question 149 appears on the next page

Over the past month...

149. Did you eat **salad dressing**?

☐ NO (GO TO INTRODUCTION TO QUESTION 150)
☐ YES

149a. How often was the salad dressing you ate **light**, **low-fat**, or **fat-free**?

☐ Almost never or never
☐ About ¼ of the time
☐ About ½ of the time
☐ About ¾ of the time
☐ Almost always or always

The following two questions ask you to summarize your usual intake of vegetables and fruits. Please do not include salads, potatoes, or juices.

150. Over the past month, how many servings of **vegetables** (not including salad or potatoes) did you eat per week or per day?

☐ Less than 1 per week
☐ 1-2 per week
☐ 3-4 per week
☐ 5-6 per week
☐ 1 per day
☐ 2 per day
☐ 3 per day
☐ 4 per day
☐ 5 or more per day

151. Over the past month, how many servings of **fruit** (not including juices) did you eat per week or per day?

☐ Less than 1 per week
☐ 1-2 per week
☐ 3-4 per week
☐ 5-6 per week
☐ 1 per day
☐ 2 per day
☐ 3 per day
☐ 4 per day
☐ 5 or more per day

152. Over the past month, which of the following foods did you eat **AT LEAST THREE TIMES**? ***(Mark all that apply.)***

☐ Avocado, guacamole
☐ Beef jerky
☐ Cheesecake
☐ Chocolate, fudge, or butterscotch toppings or syrups
☐ Chow mein noodles
☐ Croissants
☐ Dark chocolate
☐ Dried apricots
☐ Dried curcumin, turmeric
☐ Dried oregano, rosemary, thyme
☐ Egg rolls
☐ Fresh basil, cilantro, or parsley
☐ Game Meat (such as bison, wild goose, venison)
☐ Granola bars
☐ Hot peppers
☐ Jell-O, gelatin
☐ Mangoes
☐ Olives
☐ Pickles or pickled vegetables or fruit
☐ Plantains
☐ Pork neck bones, hock, head, feet
☐ Pudding or custard
☐ Sushi
☐ Veal, lamb
☐ Whipped cream, regular
☐ Whipped cream, substitute

☐ NONE

153. For **ALL** of the past month, have you followed any type of **vegetarian diet**?

☐ NO (GO TO INTRODUCTION TO QUESTION 154)
☐ YES

153a. Which of the following foods did you **TOTALLY EXCLUDE** from your diet? ***(Mark all that apply.)***

☐ Meat (beef, pork, lamb, etc.)
☐ Poultry (chicken, turkey, duck, etc.)
☐ Fish and shellfish
☐ Eggs
☐ Dairy products (milk, cheese, etc.)

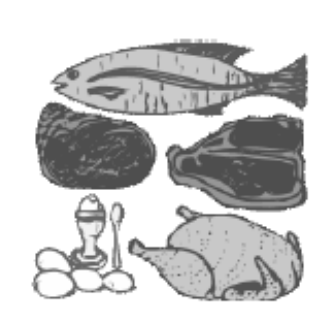

Introduction to Question 154 appears on the next page

The next questions are about your use of vitamin pills or other supplements.

154. Over the past month, did you take any **multivitamins**, such as One-a-Day-, Centrum-, or Prenatal-type multivitamins (as pills, liquids, or packets)?

- ☐ NO (GO TO INTRODUCTION TO QUESTION 156)
- ☐ YES

155. How often did you take **One-a-Day-, Centrum-,** or **Prenatal-type** multivitamins?

- ☐ 1-3 days in past month
- ☐ 1-3 days per week
- ☐ 4-6 days per week
- ☐ Every day

155a. Did your **multivitamin** usually contain **minerals** (such as iron, zinc, etc.)?

- ☐ NO
- ☐ YES
- ☐ Don't know

155b. Was your **multivitamin** usually a **Prenatal-type?**

- ☐ NO
- ☐ YES
- ☐ Don't know

155c. Over the past month, did you take any **vitamins, minerals**, or **herbal supplements** other than your multivitamin?

- ☐ NO (GO TO QUESTION 165)
- ☐ YES (GO TO INTRODUCTION TO QUESTION 156)

Question 165 appears on page 39

Introduction to Question 156 appears in the next column

These last questions are about the vitamins, minerals, or herbal supplements you took that are NOT part of a One-a-Day-, Centrum-, or Prenatal-type of multivitamin.

Over the past month...

156. How often did you take **Antacids that contain Calcium such as Tums or Rolaids**?

- ☐ NEVER (GO TO QUESTION 157)
- ☐ 1-3 days in past month
- ☐ 1-3 days per week
- ☐ 4-6 days per week
- ☐ Every day

156a. When you took **Antacids that contain Calcium such as Tums or Rolaids**, about how many tablets or lozenges did you take in one day?

- ☐ Less than 1
- ☐ 1
- ☐ 2
- ☐ 3
- ☐ 4 or more
- ☐ Don't know

156b. Was your Antacid usually **extra strength**?

- ☐ NO
- ☐ YES
- ☐ Don't know

157. How often did you take **Calcium** (with or without Vitamin D) (**NOT** as part of a multivitamin in Question 155 or antacid in Question 156)?

- ☐ NEVER (GO TO QUESTION 158)
- ☐ 1-3 days in past month
- ☐ 1-3 days per week
- ☐ 4-6 days per week
- ☐ Every day

157a. When you took **Calcium**, about how much elemental calcium did you take in one day? *(If possible, please check the label for elemental calcium.)*

- ☐ Less than 500 mg
- ☐ 500-599 mg
- ☐ 600-999 mg
- ☐ 1,000 mg or more
- ☐ Don't know

Question 158 appears on the next page

Over the past month...

157b. Did your **Calcium** usually contain **Vitamin D**?

- ☐ NO
- ☐ YES
- ☐ Don't know

157c. Did your **Calcium** usually contain **Magnesium**?

- ☐ NO
- ☐ YES
- ☐ Don't know

157d. Did your **Calcium** usually contain **Zinc**?

- ☐ NO
- ☐ YES
- ☐ Don't know

158. How often did you take **Vitamin D** on its own or as part of a calcium supplement (**NOT** as part of a multivitamin in Question 155)?

- ☐ NEVER (GO TO QUESTION 159)
- ☐ 1-3 days in past month
- ☐ 1-3 days per week
- ☐ 4-6 days per week
- ☐ Every day

158a. When you took **Vitamin D**, about how much did you take in one day?

- ☐ Less than 400 IU
- ☐ 400-799 IU
- ☐ 800-999 IU
- ☐ 1,000 IU or more
- ☐ Don't know

159. How often did you take **Iron** (**NOT** as part of a multivitamin in Question 155)?

- ☐ NEVER (GO TO QUESTION 160)
- ☐ 1-3 days in past month
- ☐ 1-3 days per week
- ☐ 4-6 days per week
- ☐ Every day

Question 160 appears in the next column

160. How often did you take **Folic Acid** (**NOT** as part of a multivitamin in Question 155)?

- ☐ NEVER(GO TO QUESTION 161)
- ☐ 1-3 days in past month
- ☐ 1-3 days per week
- ☐ 4-6 days per week
- ☐ Every day

160a. When you took **Folic Acid**, about how much did you take in one day?

- ☐ Less than 0.4 mg
- ☐ 0.4-0.9 mg
- ☐ 1.0 mg
- ☐ More than 1.0 mg
- ☐ Don't know

161. How often did you take **Vitamin C** (**NOT** as part of a multivitamin in Question 155)?

- ☐ NEVER (GO TO QUESTION 162)
- ☐ 1-3 days in past month
- ☐ 1-3 days per week
- ☐ 4-6 days per week
- ☐ Every day

161a. When you took **Vitamin C**, about how much did you take in one day?

- ☐ Less than 500 mg
- ☐ 500-999 mg
- ☐ 1,000-1,499 mg
- ☐ 1,500-1,999 mg
- ☐ 2,000 mg or more
- ☐ Don't know

162. How often did you take **Vitamin E** (**NOT** as part of a multivitamin in Question 155)?

- ☐ NEVER (GO TO INTRODUCTION TO QUESTION 163)
- ☐ 1-3 days in past month
- ☐ 1-3 days per week
- ☐ 4-6 days per week
- ☐ Every day

162a. When you took **Vitamin E**, about how much did you take in one day?

- ☐ Less than 400 IU
- ☐ 400-799 IU
- ☐ 800-999 IU
- ☐ 1,000 IU or more
- ☐ Don't know

Introduction to Question 163 appears on the next page

The next two questions ask you about other supplements you took more than once per week.

163. Please mark any of the following **single supplements** you took more than once per week (**NOT** as part of a multivitamin in Question 155):

- ☐ B-6
- ☐ B-complex
- ☐ B-12
- ☐ Beta-carotene
- ☐ Magnesium
- ☐ Occu-vite/Eye health
- ☐ Potassium
- ☐ Selenium
- ☐ Vitamin A
- ☐ Zinc

164. Please mark any of the following **herbal, botanical, or other supplements** you took more than once per week.

- ☐ Chondroitin
- ☐ Coenzyme Q-10
- ☐ Echinacea
- ☐ Energy supplements
- ☐ Fish oil/Omega-3's
- ☐ Flaxseed oil
- ☐ Garlic
- ☐ Ginger
- ☐ Ginkgo biloba
- ☐ Ginseng
- ☐ Glucosamine
- ☐ Peppermint
- ☐ Probiotics
- ☐ Red clover
- ☐ Saw palmetto
- ☐ Soy supplement
- ☐ Sports supplements
- ☐ St. John's wort
- ☐ Other

165. Is there anything else you ate in the past month? Please write name of food, frequency and amount (optional).

____

____

____

____

____

____

____

____

Thank you *very much* for completing this questionnaire! Because we want to be able to use all the information you have provided, we would greatly appreciate it if you would please take a moment to review each page making sure that you:

- **Did not skip any pages and**
- **Crossed out the incorrect answer and circled the correct answer if you made any changes.**

14.11 Bristol Stool Chart

ID #: ____________________

Date of fecal sample: ____________________

Please circle the fecal matter type that is most representative of your sample:

Bristol Stool Form Scale (English for Canada)

Type 1	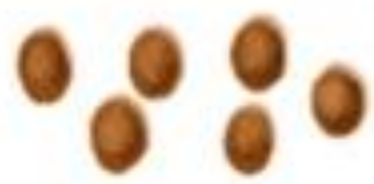	Separate hard lumps, like nuts
Type 2	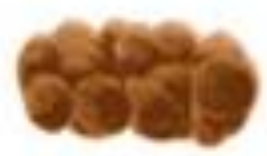	Sausage-shaped but lumpy
Type 3	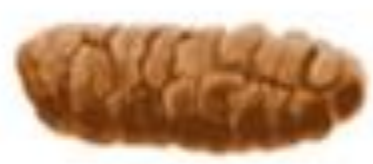	Like a sausage but with cracks on the surface
Type 4	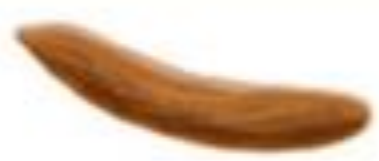	Like a sausage or snake, smooth and soft
Type 5	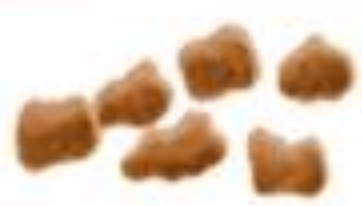	Soft blobs with clear-cut edges
Type 6	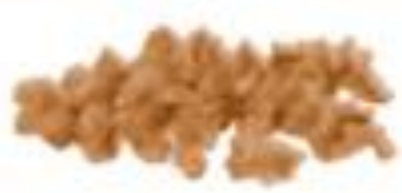	Fluffy pieces with ragged edges, a mushy stool
Type 7	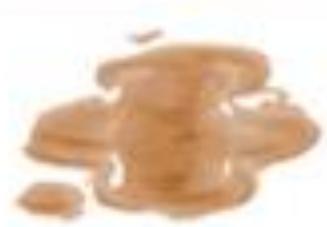	Watery, no solid pieces.

Copyright 2000 © by Rome Foundation. All Rights Reserved.
Updated by RF July 2019

14.12 MIND Diet Score

Diet component	0	0.5	1
Green leafy[*] vegetables	≤2 servings/wk	>2 to <6/wk	≥6 servings/wk
Other vegetables[†]	<5 serving/wk	5 to <7 wk	≥1 serving/d
Berries[‡]	<1 serving/wk	1/wk	≥2 servings/wk
Nuts	<1/mo	1/mo to <5/wk	≥5 servings/wk
Olive oil	Not primary oil		Primary oil used
Butter, margarine	>2 T/d	1–2/d	<1 T/d
Cheese	7 + servings/wk	1–6/wk	<1 serving/wk
Whole grains	<1 serving/d	1–2/d	≥3 servings/d
Fish (not fried)[‖]	Rarely	1–3/mo	≥1 meals/wk
Beans[§]	<1 meal/wk	1–3/wk	>3 meals/wk
Poultry (not fried)[¶]	<1 meal/wk	1/wk	≥2 meals/wk
Red meat and products[#]	7 + meals/wk	4–6/wk	<4 meals/wk
Fast fried foods[**]	4 + times/wk	1–3/wk	<1 time/wk
Pastries and sweets[††]	7 + servings/wk	5–6/wk	<5 servings/wk
Wine	>1 glass/d or never	1/mo–6/wk	1 glass/d
Total score			15

Abbreviation: MIND, Mediterranean-DASH diet intervention for neurodegenerative delay.

*Kale, collards, greens; spinach; lettuce/tossed salad.

†Green/red peppers, squash, cooked carrots, raw carrots, broccoli, celery, potatoes, peas or lima beans, tomatoes, tomato sauce, string beans, beets, corn, zucchini/summer squash/eggplant, coleslaw, potato salad.

‡Strawberries.

§Beans, lentils, soybeans.

‖Tuna sandwich, fresh fish as main dish; not fried fish cakes, sticks, or sandwiches.

¶Chicken or turkey sandwich, chicken or turkey as main dish, and never eat fried at home or away from home.

#Cheeseburger, hamburger, beef tacos/burritos, hot dogs/sausages, roast beef or ham sandwich, salami, bologna, or other deli meat sandwich, beef (steak, roast) or lamb as main dish, pork or ham as main dish, meatballs or meatloaf.

**How often do you eat fried food away from home (like French fries, chicken nuggets)?

††Biscuit/roll, poptarts, cake, snack cakes/twinkies, Danish/sweet rolls/pastry, donuts, cookies, brownies, pie, candy bars, other candy, ice cream, pudding, and milkshakes/frappes.

14.13 Mediterranean Diet Adherence Screener

	Foods and frequency of consumption	Criteria for 1 point*
1	Do you use olive oil as main culinary fat?	Yes
2	How much olive oil do you consume in a given day (including oil used for frying, salads, out of house meals, etc.)?	4 or more tablespoons
3	How many vegetable servings do you consume per day? *(1 serving = 200g - consider side dishes as 1/2 serving)*	2 or more *(at least 1 portion raw or as salad)*
4	How many fruit units (including natural fruit juices) do you consume per day?	3 or more
5	How many servings of red meat, hamburger, or meat products (ham, sausage, etc.) do you consume per day? *(1 serving = 100-150 g)*	Less than 1
6	How many servings of butter, margarine, or cream do you consume per day? *(1 serving = 12 g)*	Less than 1
7	How many sweet/carbonated beverages do you drink per day?	Less than 1
8	How much wine do you drink per week?	7 or more glasses
9	How many servings of legumes do you consume per week? *(1 serving = 150 g)*	3 or more
10	How many servings of fish or shellfish do you consume per week? (1 serving: 100-150 g fish, or 4-5 units or 200 g shellfish)	3 or more
11	How many times per week do you consume commercial sweets or pastries (not homemade), such as cakes, cookies, biscuits, or custard?	Less than 3
12	How many servings of nuts (including peanuts) do you consume per week? *(1 serving = 30 g)*	3 or more
13	Do you preferentially consume chicken, turkey or rabbit meat instead of veal, pork, hamburger or sausage?**	Yes
14	How many times per week do you consume vegetables, pasta, rice, or other dishes seasoned with *sofrito* (sauce made with tomato and onion, leek, or garlic, simmered with olive oil)?	2 or more

* 0 points if these criteria are not met.

** 1 point for vegetarians.

14.14 Nutrition Information Form

Nutrition Information Form

Adapted from Charlie Foundation's Diet & Nutrition History Form

Participant ID: ______________________

Height: __________ **Weight:** ___________

Has there been any recent change in your weight? If yes, please explain:

____

Male: _____ **Female:** _____

Medicines you are currently taking:

Name	Dose	Dose time	Manufacturer

Vitamins or other supplements you are currently taking:

Name	Dose	Dose time	Manufacturer

GI issues: _____Nausea _____Vomit _____Reflux

Bowel Habits: ___ Normal ______ Constipation ______ Loose Stools

Types of Work: ____

Physical activities each week: ______ minutes light; _____ minutes moderate; _____ minutes vigorous

Food Allergies: ____

Food Intolerances: ____

Chewing Difficulties: ________________ **Swallowing Difficulties:** _____________

Dietary Pattern:

Please record a typical day's diet

Specific amounts of food are very helpful for our assessment:

Meal	Time	Food and Beverages
Breakfast		
Snacks		
Lunch		
Snacks		
Supper		
Snacks		

Indicate your food preferences below by checking the boxes to the left of the foods:

Beverages

□ Water	□ Herbal Tea	□ Diet Soda	□ Coffee	□ Decaf Coffee

Fruit

□ Raspberries	□ Strawberries	□ Blueberries	□ Blackberries	

Vegetables

□ Asparagus	□ Radishes	□ Beets	□ Kohlrabi	□ Beet Greens
□ Rhubarb	□ Broccoli	□ Mushroom	□ Cabbage	□ Sauerkraut
□ Brussels Sprouts	□ Mustard Greens	□ Celery	□ Okra	□ Chicory
□ Summer Squash	□ Swiss Chard	□ Carrots	□ Onions	□ Cucumbers
□ Tomatoes	□ Cauliflower	□ Rutabaga	□ Eggplant	□ Tomato Juice
□ Endive	□ Turnips	□ Green Pepper	□ Turnip Greens	□ Winter Squash
□ Dandelion Greens	□ Poke	□ Watercress	□ Kale	□ Romaine
□ Lettuce	□ Spinach	□ Avocado		

Protein

□ Hard Boiled Eggs	□ Scrambled Eggs	□ Egg Salad	□ Pork Chop	□ Ground Beef
□ Chicken	□ Turkey	□ Rabbit	□ Lamb	□ Veal
□ Cheddar Cheese	□ Swiss Cheese	□ Mozzarella Cheese	□ Cottage cheese	□ Greek Yogurt
□ Chickpea	□ Black beans	□ Edamame	□ Lentils	□ Kidney beans
□ Tofu	□ Tempeh	□ Peanuts	□ Almond	□ Hazelnut
□ Pecans	□ Macadamia	□ Sesame seeds	□ Sunflower Seeds	□ Pumpkin Seeds
□ Salmon	□ Mackerel	□ Tuna	□ Anchovies	□ Herring
□ Black Cod	□ Crab	□ Lobster	□ Scallops	□ Shrimp

Fats

□ Olive Oil	□ Butter	□ Ghee	□ Mayonnaise	□ Grapeseed Oil
□ Walnut Oil	□ Avocado oil	□ Avocado		

Other favorite foods:

14.15 Parkinson's Disease Cognitive Rating Scale (PD-CRS)

1. IMMEDIATE FREE RECALL VERBAL MEMORY

Words	1	Words	2	Words	3
LIGHT		STAR		LION	
SILK		TIE		STAR	
SAND		FRAGRANCE		BICYCLE	
EYELASH		LIGHT		RICE	
RICE		RING		BLACKBOARD	
TIE		RICE		LIGHT	
BLACKBOARD		EYELASH		SAND	
BICYCLE		SILK		TIE	
STAR		BLACKBOARD		RING	
LION		SAND		FRAGRANCE	
RING		BICYCLE		EYELASH	
FRAGRANCE		LION		SILK	
Total		Total		Total	

2. CONFRONTATION NAMING

	1		1
BIB		BUCKLE	
CANDLE		MANE	
CHERRY		HOOK	
STOOL		SCREWDRIVER	
ANCHOR		PANEL SCREEN	
TURTLE		SAFETY PIN	
KITE		JINGLE BELL	
FISHBOWL		HOOF	
BULB		EXTINGUISHER	
GUITAR		DOOR BOLT	
		Total	

3. SUSTAINED ATTENTION

2 L T (Example)	2 letters	
8 A 9 (Example)	1 letter	
2 P 6 5 4	1 letter	
3 A 6 K L	3 letters	
B 9 0 4 L T	3 letters	
3 C P 5 7 3	2 letters	
3 9 5 L 4 Z A	3 letters	
i 1 A S Q 4 1	4 letters	
7 5 D A 4 T B 2	4 letters	
9 6 8 4 3 7 L C	2 letters	
Z 4 9 A T D 3 8 4	4 letters	
9 5 M D 4 S C 3 E	5 letters	
	Total	

4. WORKING MEMORY

L 2 T (Example)	2 L T	
8 A 9 (Example)	8 9 A	
M 3	3 M	
7 P	7 P	
G 8 M	8 G M	
9 i 6	6 9 i	
T 0 4 A	0 4 A T	
7 V 6 J	6 7 J V	
M 6 4 N i	4 6 i M N	
3 5 S G C	3 5 C G S	
1 R 9 V B 3	1 3 9 B R V	
M 2 7 4 Z 9	2 4 7 9 M Z	
	Total	

5. UNPROMPTED CLOCK

7. DELAYED V. M.

Words	
LIGHT	
SILK	
SAND	
EYELASH	
RICE	
TIE	
BLACKBOARD	
BICYCLE	
STAR	
LION	
RING	
FRAGRANCE	
Total	

8. ALTERNATING VERBAL FLUENCY

1		2	
3		4	
5		6	
7		8	
9		10	
11		12	
13		14	
15		16	
17		18	
19		20	
S			

9. ACTION VERBAL FLUENCY

1		16	
2		17	
3		18	
4		19	
5		20	
6		21	
7		22	
8		23	
9		24	
10		25	
11		26	
12		27	
13		28	
14		29	
15		30	

Name: *Date:*

PD-CRS

6. CLOCK COPY

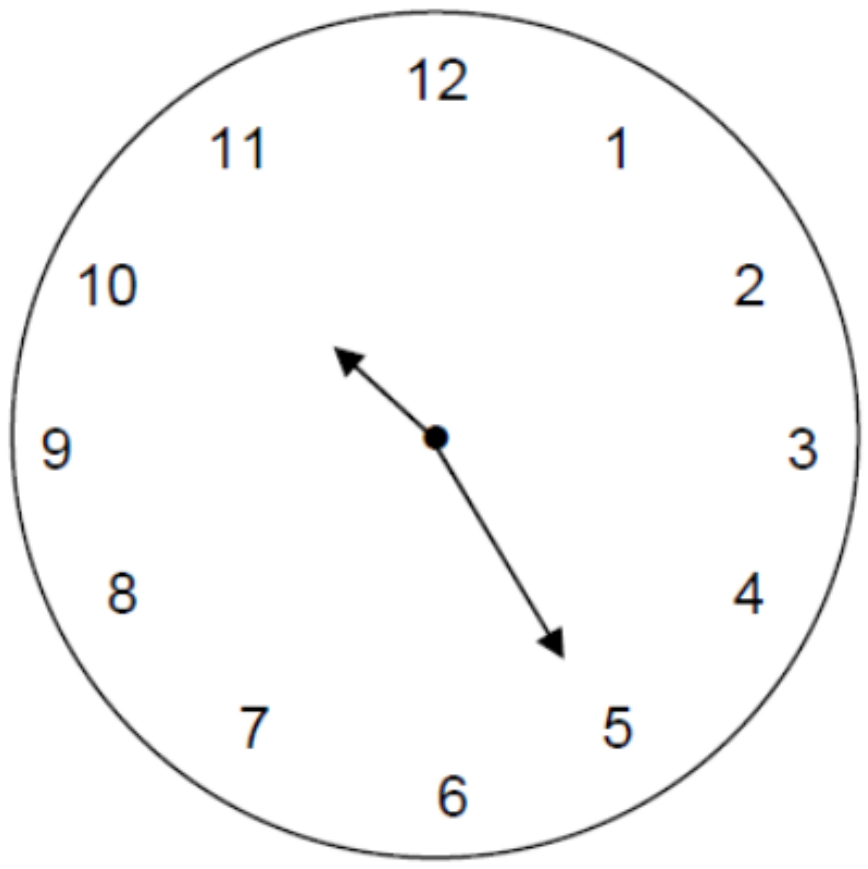

	Unprom		Copy	
	Y	N	Y	N
The figure looks like a clock				
The clock is not divided by lines/sectors				
Symmetric disposition of numbers				
Only 1 to 12 numbers are drawn				
Hours numbers correctly sequenced				
Only two hands are drawn				
Clock hands are represented as arrows				
Hour hand shorter. Correct time				
No words have been written.				
The number “25” has not been drawn				
**Score (0-10)**				

Item	*Score*
1. Immediate free recall	/12
2. Confrontation naming	/20
3. Sustained Attention	/10
4. Working memory	/10
5. Unprompted drawing of a clock	/10
6. Copy drawing of a clock	/10
7. Delayed free recall	/12
8. Alternating verbal fluency	/20
9. Action verbal fluency	/30
**FRONTAL-SUBCORTICAL SCORE**	/104
**POSTERIOR-CORTICAL SCORE**	/30
**TOTAL SCORE**	/134

PD-CRS

14.16 Parkinson’s Disease Cognitive Functional Rating Scale (PD-CFRS)

PARKINSON'S DISEASE – COGNITIVE FUNCTIONAL RATING SCALE (PD-CFRS)

At the beginning of the questionnaire the explorer should introduce the meaning of the questions with the following sentence: ***"Now we are going to avoid the motor problems and focus on how cognitive difficulties (lack of attention, memory, mental slowness…) have hindered your daily life activities over the past two weeks".***

In the questionnaire posed to a knowledgeable informant, when appropriate "you" should be replaced by the patient's name, and "your" by "her/his".

Answered by: □ Patient

□ Informant ___________________ (relationship)

Items	*Score**			
**1. Have you had any trouble handling your own money? For example: check the change, calculate the necessary money for going shopping for groceries, etc.**	0	1	2	8
**2. Have you had trouble handling the domestic economy? For example: control finances, bank accounts, the saving money, etc.**	0	1	2	8
**3. Have you had trouble arranging your holidays or meeting with family or friends?**	0	1	2	8
**4. Have you had trouble handling the personal mail, doctor appointments or bills?**	0	1	2	8
**5. Have you had trouble controlling doses and the time you have to take your own medicines?**	0	1	2	8
**6. Have you had trouble in organizing your daily activities?**	0	1	2	8
**7. Have you had any difficulty in understanding how to handle the home electrical appliances?**	0	1	2	8
**8. Have you had trouble knowing how to use public transport to get to a specific place?**	0	1	2	8
**9. Have you had problems solving unforeseen events?**	0	1	2	8
**10. Have you had trouble to explain the things you want to say?**	0	1	2	8
**11. Have you had trouble in understanding the things you read: books, magazines or the newspaper?**	0	1	2	8
**12. Have you had trouble to understand how to handle the cell phone?**	0	1	2	8
0: No.<br>*1: Some of the time.*<br>*2: Most of the time.*<br>*8: The subject has never done the activity in the past.*	Total:____ (0-24)			

** To obtain the final score, the average of all the items rated from 0 to 2 should be calculated, and turn to the nearest integer value (0-1-2) (eg. 1.52 = 2). The value obtained must replace all those items answered with 8. The total score is the sum of the 12 items once every 8 was replaced.*

© J. Kulisevsky
